# Cooperative Ligand-Mediated Transitions in Simple Macromolecules

**DOI:** 10.1021/acs.jpcb.5c05386

**Published:** 2025-10-28

**Authors:** James L. Martin Robinson, Neshat Moslehi, Nikolaos Dramountanis, Lennart van den Hoven, Alexander M. van Silfhout, Kanvaly S. Lacina, Mies van Steenbergen, Wessel Custers, Bas G. P. van Ravensteijn, Willem K. Kegel

**Affiliations:** † Van’t Hoff Laboratory for Physical and Colloid Chemistry, Debye Institute for Nanomaterials Science, 8125 Utrecht University, 3584 CH Utrecht, The Netherlands; ‡ Department of Pharmaceutics, Utrecht Institute for Pharmaceutical Sciences (UIPS), 8125 Utrecht University, 3584 CG Utrecht, The Netherlands

## Abstract

In biology, ligand-mediated transitions (LMT), where the binding of a molecular ligand onto the binding site of a receptor molecule leads to a well-defined change in the conformation of the receptor, are often referred to as “the second secret of life.” Sharp, cooperative transitions arise in many biological cases, while examples of synthetic cooperative systems are rare. This is because well-defined conformational states are hard to “program” into a molecular design. Here, we impose an external constraint in the form of two immiscible liquids that effectively define and limit the available conformational states of two different synthetic and relatively simple macromolecules. We show that the mechanism of the observed cooperative transitions with ligand concentration is the coupling of ligand binding and conformation, similar to more complex biological systems. The systems studied are (1) hydrophobic polyelectrolytes (HPE) which are (bio) polymers that consist of hydrophobic as well as ionizable (proton and hydroxyl ligand binding) functional groups. (2) Oligomeric metal chelators (OMC), which are oligomers composed of metal ion chelating repeating groups that are able to bind metal ions (considered as the “ligands”), resulting in gel-like networks of oligomers cross-linked by coordinated metal ions. We find that in HPE, interactions between ligands and individual macromolecules explain the observed cooperative transitions. For OMC, coordinated bonds significantly enhance the degree of cooperativity, compared to HPE.

## Graphical abstract

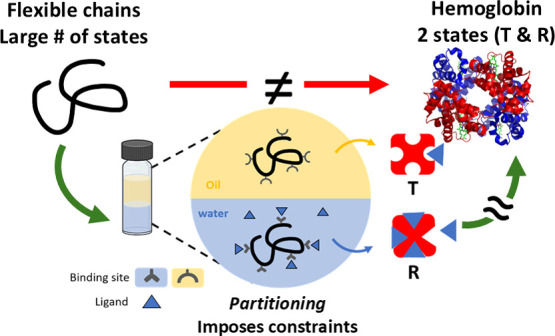

## Introduction

Many of the sharp, switch-like transitions seen in biological macromolecules can be attributed to the ubiquitous Monod–Wyman–Changeaux (MWC) mechanism.^1,2^ The central concept of MWC is the competition between conformational states of the protein and the strength of the ligand binding interaction. In general, the ground-state conformation has a relatively low affinity for ligands, while a conformationally unfavorable state has a high­(er) affinity. Upon increasing the concentration of the ligands, a sharp transition can be observed from the ground state to the unfavorable conformation, which is stabilized by ligand binding. This relies on highly ordered macromolecular protein structures, which constrain the number of thermally accessible conformational states. In that way, transitions only occur between these well-defined conformations.

The archetypal cooperative ligand-mediated transition (LMT) is the binding of oxygen to hemoglobin. This protein, found in most vertebrates, is composed of four individual globin protein units. Each of these contains a heme group, which acts as an oxygen-binding site. The explanation for the cooperativity hinges on the notion of a competition between the conformational state of the protein and the binding energy of oxygen. The T (Tense) state is the conformational ground state, but presents weaker binding; conversely, the R (Relaxed) state is an unfavorable conformation for the protein but presents a higher affinity for oxygen. The situation is schematically summarized in Figure 1a. More details can be found in a previous work.^3^ At this point, we emphasize that the only requirements for MWC-like transitions are (1) at least two conformational states, where each state has a characteristic binding affinity to the ligands, and (2) the conformationally unfavorable state has a stronger affinity for the ligands.^2,3^

**Figure 1:**
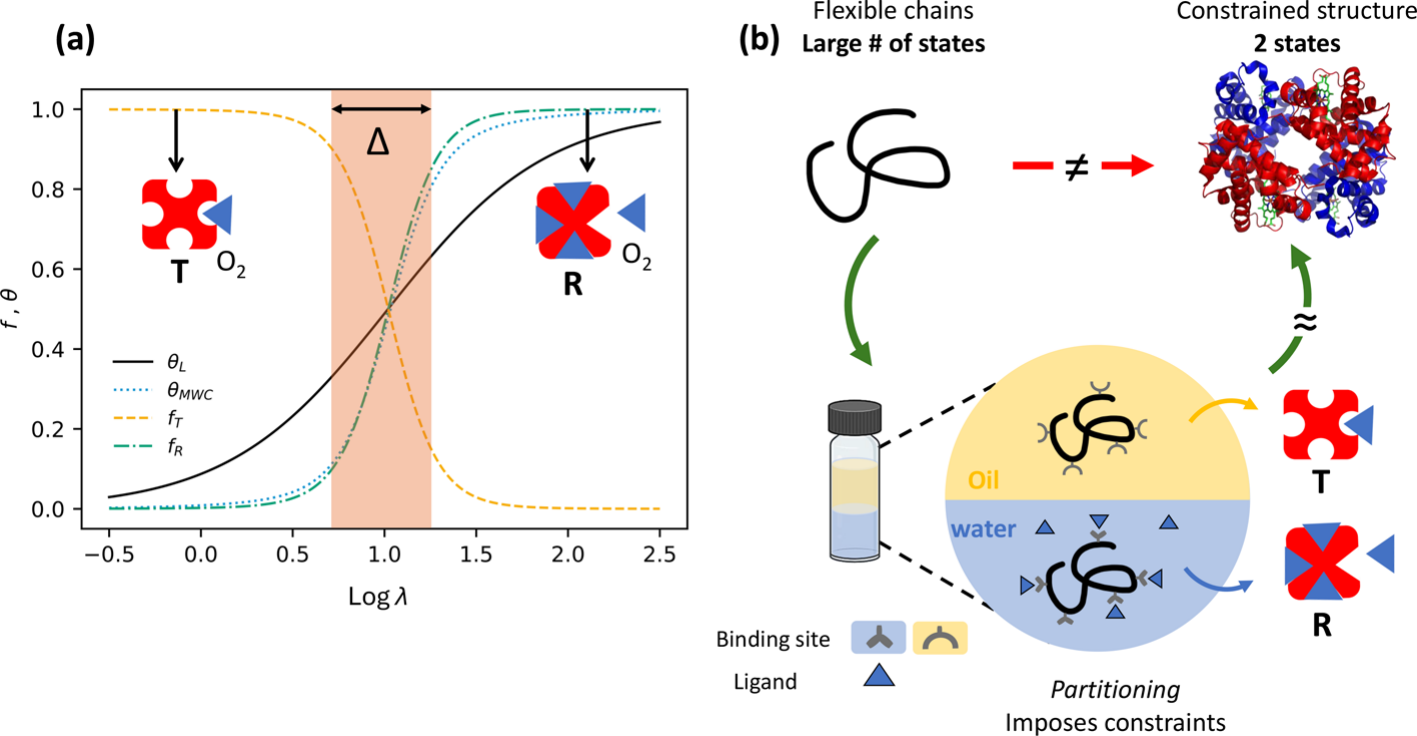
Sharp, cooperative, ligand-mediated transitions are predicted for simple oligomeric species. (a) Plot of the fraction of hemoglobin in the Relaxed (*f*_R_) and Tense (*f*_T_) states, and the fraction of bound oxygen onto hemoglobin, θ_MWC_, as a function of λ. λ is the oxygen fugacity and is proportional to the partial oxygen pressure. For details, see our previous work.^3^ The Langmuir isotherm θ_L_ is eq 2, with β*g* = −6. Δ is a measure for the width, or sharpness, of the oxygen-ligand-mediated transition (LMT). (b) Schematic of the analogy between a conformationally constrained oligomer and hemoglobin. The oligomer in isolation presents many thermally accessible conformations compared to the two main conformations for hemoglobin. However, when placed in a two-state oil and water system, the oligomer is funneled into a reduced set of accessible conformations, which, coupled to the preferential ion binding in the aqueous phase, leads to sharp, cooperative transitions.

Several classes of cooperative transitions can be distinguished, for example, phase transitions, superconductivity, magnetization, and several more in more complex molecular systems.^2,4,5^ Here we focus on LMT. Observations of significant cooperativity in LMT in nonbiological systems are rare. Exceptions we are aware of are (1) the binding of oxygen and other small molecules onto solid iron-porphyrin derivatives^6^ and onto helical poly-l-lysine heme complex,^7^ and (2) synthetic, low-molecular-weight ligands onto aggregates of modified cyclodextrins have been observed to bind cooperatively, possibly by an MWC mechanism.^8^ In these examples, the observed degree of cooperativity (defined in the Theory) is usually less than that observed in oxygen binding onto hemoglobin.

We have recently shown that hydrophobic polyelectrolytes (HPE) that are able to solubilize bilayer membranes or undergo micellization as a function of pH operate by a competition between two or more conformational states and their degree of ionization. These systems fit within the framework of MWC theory, showcasing the requirement of a limited number of conformational states to realize cooperative transitions.^3^ The key idea is that ionization (deprotonation of acid groups or protonation of basic groups) can be seen as the binding of ligands in the form of hydroxyl ions or protons. The previously mentioned requirement for the number of conformational states of the macromolecules to be constrained is, in the case of HPE, provided by external conditions or conditions inherent to the macromolecular architecture. In the micellization systems,^9–12^ the polymer conformations are constrained by the hydrophilic blocks attached to them, which drives self-assembly into micelles. The core of these micelles now becomes a hydrophobic reservoir for the HPE and a homogeneous environment allowing for a two-state description for the conformational state of the polymer: freely dissolved and in the micelle core. In the case of membrane solubilization, the hydrophobic reservoir that will constrain the conformations of the HPE is the core of the lipid membranes. For these systems, the presence of the lipid vesicles funnels the many potential conformations of the HPE into three dominant ones: freely dissolved, around the edge of a lipid nanodisk, and fully immersed in the hydrophobic core of the lipid membranes.^13,14^

While coupling between conformational states and ligand binding explains the observations, the analysis in reference^3^ also points to an important difference with respect to similar transitions in biological macromolecules. In hemoglobin, for example, the conformational states are single molecular states that are available irrespective of the larger environment of hemoglobin. In HPE, on the other hand, hydrophobic and aqueous reservoirs are required, which are realized either by local phase separation of HPE-containing diblocks in the form of micellar cores^11^ or by the availability of lipid bilayer vesicles.^13,14^ In this work, we avoid the complexity inherent in micelle formation and solubilization of bilayer vesicles, and pin down the mechanism of LMT in well-defined hydrophobic and aqueous reservoirs. We design a two-phase water/oil experimental setup in which the partitioning of a weakly acidic HPE and an oligomeric metal chelator (OMC) between oil and water is studied, see Figure 1b, for a schematic representation of the experimental setup. The partitioning of the macromolecules between oil and water, as a function of the relevant ligand concentration (to be varied by pH or concentration of the metal ions), is analogous to the LMT between the conformational states of more complex macromolecules, such as the T and R states in hemoglobin.

In the following, we experimentally study two chemically distinct polymers and ligands, and also address the influence of composition dispersity, specifically the dispersity in the monomer ratio of a binary copolymer. The results will be discussed within a generalization of MWC^1^ and our recent extension for HPE.^3^

We show that these two quite different systems present cooperative partitioning transitions. Upon increasing ligand concentration (hydroxide ions via pH in HPE, and iron ions in the OMC) in the water phase, the binding energy of the ligands overcomes the unfavorable interaction between hydrophobic moieties on the polymers with water, with the effect that the polymer migrates from the oil phase (hydrophobic state) to the water phase (aqueous state). Note that we consider the metal ions as ligands. The competition between these two driving forces, analogous to the hemoglobin-oxygen system, leads to a sharp transition from the oil to the water phase over a narrow range of free ligand concentration. We emphasize here that the experimental setup, with well-defined hydrophobic (oil) and aqueous regions, has been chosen as a simplification and generalization of the more complex two-states systems mentioned above,^9–14^ and effectively selects two distinct conformational states: a hydrophobic and an aqueous state for the polymers. Thus, oil–water partitioning reflects conformational change in this context. With the experiment design mentioned above, we show that our two constructed synthetic polymers show cooperative transitions and that this cooperative behavior fits well within our generalization of the MWC model.

## Theory

The theoretical framework used in this paper to explain the cooperative binding of ionic ligands onto macromolecular templates is based on the MWC model^1,2^ mentioned in the Introduction, and which has been applied recently to HPE.^3^ In this work, the model has been extended to include OMC as well as composition dispersity. Detailed derivations are written in the SI (Section 1). Here we briefly summarize the predictions of the model.

We consider partitioning of oligomers with *M* effective binding sites for ligands over equal volumes of aqueous (aq) and oily (hydrophobic, *H*) liquid. The particular liquid phase in which the oligomer is dissolved defines the conformational state of the oligomer. The binding affinity for ligands in the hydrophobic state is assumed to be negligibly small compared to the situation in the aqueous state. The ground state of the oligomers is the hydrophobic state. This assumes oligomers with a fairly hydrophobic composition, which will preferably partition into an oily phase over an aqueous phase when the ligand concentration is low in the aqueous phase. Oligomers pay a hydrophobic penalty, *G*, analogous to a conformational penalty, when dissolved in the aqueous state. At the same time, in the aqueous state, ligand binding is favorable with a binding free energy of *g* per ligand. As derived in the SI, the fraction of oligomers in the aqueous state is given by $f_{\mathrm{aq}}=\frac{\exp(-\beta G)(1+\lambda \exp(-\beta g){)}^{M}}{1+\exp(-\beta G)(1+\lambda \exp(-\beta g){)}^{M}}$ . The value of *M* determines the sharpness of the transition from *f*_aq_ = 0 to *f*_aq_ = 1 as a function of λ and is defined as the degree of cooperativity. The fugacity or activity of the ligand, λ = exp­(βμ), with β = 1/*k*_B_ *T*. *k*_B_ is Boltzmann’s constant and *T* is the absolute temperature. μ is the chemical potential of the ligand that adsorbs (or binds) onto the macromolecular template. μ is related to the (free) ligand concentration or partial pressure of the ligand.

Further, we show that the fraction of bound ligands is given by $\theta =\langle N\rangle /M=\frac{\lambda \exp(-\beta g)}{1+\lambda \exp(-\beta g)}f_{\mathrm{aq}}$ , with ⟨*N*⟩ the average number of occupied binding sites. This implies a strong correlation between *f*_aq_ and θ, as around the transition we have λ exp­(−β*g*) ≫ 1 and *f*_aq_ ≈ θ, similar to the situation with *f*_R_ and θ for hemoglobin, see Figure 1a. Under the same condition λ exp­(−β*g*) ≫ 1, we find that $\theta \approx f_{\mathrm{aq}}\approx \frac{(\lambda \exp(-\beta (g+g_{H})){)}^{M}}{1+(\lambda \exp(-\beta (g+g_{H})){)}^{M}}$ . Here, we defined the conformational penalty per ligand binding site *g*_H_ via *G* = *Mg*_H_. This result is isomorphic to the Hill equation^15^

$$\theta_{H}=\frac{(K[L]{)}^{n_{H}}}{1+(K[L]{)}^{n_{H}}}\;(\mathrm{Hill})$$ 1

with *K* the ligand binding constant, [L] the free ligand concentration, and *n*_H_ the Hill exponent. If only a single state is available, the fraction of bound ligands reduces to the Langmuir equation

$$\theta_{L}=\frac{\lambda \exp(-\beta g)}{1+\lambda \exp(-\beta g)}$$ 2

which has been plotted in Figure 1a.

Similarly to the Hill exponent, we consider a transition with a value of *M* = 1, which is isomorphic to a Langmuir isotherm, to correspond to a noncooperative transition. Values of *M* higher than unity correspond to cooperative behavior. Compared to a, in general, strictly empirical value of the Hill exponent, we note that the value of *M* in the equations described above is directly linked to the number of binding sites on the template as well as to other properties such as the presence of intermediate states. Moreover, the theory allows for the effects of polymer (template) length (via the value of *M*) and length (size) dispersity, as well as chemical dispersity, to be predicted.

## Materials and Methods

In order to study the cooperative partitioning transitions between oil and water, two case studies were designed; the first uses weakly acidic HPE, and the second uses an iron-binding OMC. For each case study, the macromolecules were designed and synthesized, followed by thorough polymer characterization. For the HPE systems, poly­(6-(acryloyl)­aminohexanoic acid) (PAHA, DP= 18, *Đ* = 1.05) and poly­(*n*-butyl acrylate-*r*-acrylic acid) (PBA-AA, DP= 22, *Đ* = 1.1) were synthesized using reversible addition–fragmentation chain transfer (RAFT) and single electron transfer living radical polymerization (SET-LRP) techniques, respectively. In the OMC case, a terpyridine-functionalized oligomer (abbreviated as PT, DP = 16, *Đ* = 1.04) was synthesized using the SET-LRP technique. For the details of synthesis and characterization of the HPE and OMC, see Sections 2.4 and 3.2 of the SI.

Next, the transition of the synthesized macromolecules between the hydrophobic (oil; pentanol for HPE, and dichloromethane (DCM) for OMC) and aqueous phase was monitored as a function of the relevant ligand concentration in the aqueous phase (pH for HPE and iron ion concentration for OMC), and compared to that of a monoprotic carboxylic acid (MPA) for the HPE or monomeric terpyridine (*T*) for the OMC. The partitioning of PAHA, PBA-AA, PT, and *T* between the oil and water phase was quantified using UV–vis absorption, as described in detail in the SI for HPE: Sections 2.3.1 and 2.5.1, and for OMC: Sections 3.5 and 3.6. For HPE, the ionization fraction was measured via potentiometric titrations (Sections 2.3.2 and 2.5.2 of the SI), and for OMC, the free iron concentration was measured via UV–vis absorption and atomic emission spectroscopy (Section 3.3.1 of the SI). Full description of the experiments, quantification, and data treatment are listed in the SI.

## Results and Discussion

### Experimental Observation of Cooperative LMT

In this section, we show experimentally in two chemically quite different oligomers, HPE and OMC, that the fundamental signatures of LMT that occur in oxygen binding onto hemoglobin, see Figure 1, are reproduced in partitioning experiments. These features are (1) the transition is cooperative, that is, it occurs over a narrow range of ligand concentration and is quantified by a Hill exponent *n*_H_ > 1; (2) a strong correlation between the fraction of the macromolecule in the conformational state with the strongest binding affinity for the ligand and the fraction of bound ligand. The last feature can be verified by comparing the correlated *f*_R_ and θ in Figure 1 for hemoglobin with *f*_aq_ and θ, see the Theory, for HPE, as well as for OMC.

Next, we present our observation that compositionally disperse HPE, specifically copolymers with disperse comonomer ratios, display significant broadening of the LMT in HPE. This effect is quantitatively explained in our extension of the MWC model.

#### Partitioning Behavior of a Homopolymer HPE

A simple, qualitative confirmation that HPE presents cooperative partitioning behavior would be a marked increase in the sharpness of such a transition compared to a simple monoprotic acid or base. To investigate this, we synthesized a homopolymeric acidic HPE, poly­(6-(acryloyl)­aminohexanoic acid) (PAHA), of 18 units in length (DP= 18). 6-(acryloyl)­aminohexanoic acid was synthesized and then polymerized using a trithiocarbonate RAFT agent to yield an oligomer with a low polydispersity index, *Đ* = 1.05 (see SI Section 2.4.1 for details). We then performed titration experiments in a two-phase pentanol-water system, with equal volumes of the phases (1.5–2 mL) and over a wide range of pH values (3–12) with a stock concentration of 1 mg/mL for PAHA in pentanol. We measured the fraction of polymer chains in the pentanol phase via UV–vis absorption and the ionization fraction via potentiometric titration as fully described in the SI (Section 2.3).

The experiment is illustrated in Figure 2a, where a schematic of the partitioning of a monoprotic carboxylic acid (MPA) and PAHA is shown. Note that upon the same increase in the free ligand concentration of the aqueous phase (which is equivalent to increasing the pH of the aqueous phase), PAHA is expected to predominantly shift from the hydrophobic to the aqueous phase, while MPA will show a broad transition. The partitioning behavior of some simple monoprotic carboxylic acids is presented in SI Figure S3. The adapted literature data show that the main feature of the pH-dependent partitioning of simple acids is a wide transition of around 3 pH units.

**Figure 2:**
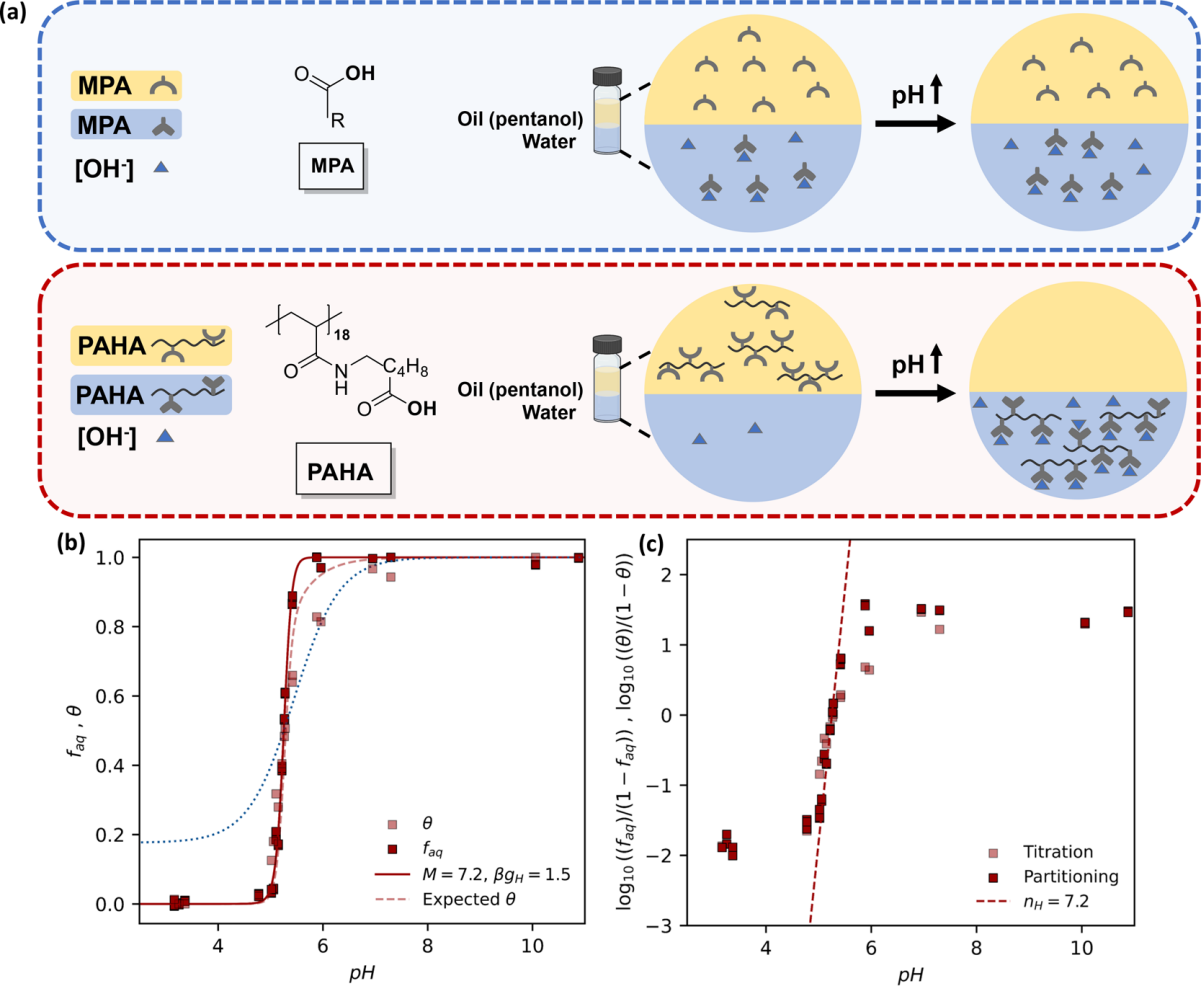
Homopolymeric HPE presents sharp, cooperative partitioning transitions. (a) Schematic of the comparison of the pH-dependent oil and water partitioning between a monoprotic acid (MPA) and the HPE PAHA. During a small change in pH (Δ), there is a complete transfer of HPE between phases, while there is only a small change in partitioning for the MPA. (b) Plot of *f*_aq_ (1 – *f*_H_) (red squares) and θ (light red squares) for PAHA (DP = 18). The data are a combination of two individual runs, where all repeat measurements are plotted. The *f*_aq_ curve is fit to eq 3 (red line) and using the p*K*_a_ value for propanoic acid: 4.69.^16^ Fit parameters are shown in the legend. As a comparison, a (calculated) noncooperative transition with *M* = 1 is plotted as a dotted blue line. Predicted θ curve (light red dashed line) using the fit parameters for eq 3 of the *f*_H_ data into eq 4. (c) Hill plots for the fraction of ionized sites and the fraction of chains in the oil for PAHA. A straight line with a Hill parameter (*n*_H_) of 7.2 is consistent with the straight line section of this graph. See SI (Section 2.5) for details on data treatment and data scaling.

For an acidic HPE partitioning system, our general ligand-mediated cooperative transition equations are applied to an HPE with weak acid groups (SI Section 1.1):

$$f_{\mathrm{aq}}=\frac{[\exp(-\beta g_{H})(1+10^{\mathrm{pH}-pK_{a}}){]}^{M}}{1+[\exp(-\beta g_{H})(1+10^{\mathrm{pH}-pK_{a}}){]}^{M}}(\mathrm{HPE})$$ 3

The fraction of occupied binding sites is then:

$$\theta =\frac{10^{\mathrm{pH}-pK_{a}}}{1+10^{\mathrm{pH}-pK_{a}}}f_{\mathrm{aq}}(\mathrm{HPE})$$ 4

In eqs 3 and 4, p*K*_a_ = −log_10_ *K*_a_, with *K*_a_ the dissociation constant of carboxylic acid groups. Ionization is considered as binding of hydroxyl ion ligands, hence the pH*-*dependent term in eq 3.^3^ The correlation between the ionized fraction and fraction of HPE in the aqueous state is strongest when pH – p*K*_a_ ≫ 0. The ionization state of the chains was determined from a potentiometric titration of the aqueous phase, while the fraction of chains in the oil phase was followed via UV–vis absorption of the UV-active end group (see SI Section 2.4.1 for details).

The number of ionizable groups per oligomer serves as an upper bound to the value of *M*. A value of *M* lower than the number of ionizable groups can be due to Coulomb interactions between ionized groups, partially ionized intermediate HPE states between the aqueous and hydrophobic states,^3^ and composition dispersity, to be addressed shortly.

Figure 2b presents in red the fraction of the polymer in the aqueous phase (1 – *f*_H_ = *f*_aq_) of the two-phase system. The curve was derived from the UV–vis absorbance (308 nm) of the RAFT agent in each pentanol solution compared to the stock solution. Figure S10 and SI Section 2.5.1.1 show the UV–vis absorption curves and explain the data treatment employed to extract the data. The main feature, we may initially point out, is the sharp transition occurring over around 0.5 pH units, leading to a complete transfer from one phase to the other. As a comparison to a noncooperative transition, a curve (blue dotted) with an *M* = 1 value has been plotted with the same p*K*_a_ and hydrophobic penalty, β*g*_H_, as was found for the polymer.

Fitting the curve for the HPE partitioning with eq 3 leads to a value of *M* of 7. Although clearly higher than for a noncooperative transition, this value is far from the expected 18 derived from the length of the polymer. As discussed further in the SI (Section 1), this effect is most likely due to the presence of intermediate conformational states of the polymer, which broaden the transition significantly. For this system in particular, we might expect that if the polymer is not fully ionized when it transitions to the aqueous phase, the Coulombic repulsions between the different charged groups will change as a function of the ionization fraction, leading to changes in the conformations of the polymer. Our experimental procedure does not allow for the elucidation of the particular conformational state of the polymer in either of the phases; however, the titration data derived from this experiment, in parallel to the hydrophobic fraction data, gives us more insight into the nature of this transition.

The light red squares in Figure 2b show the curve of the fraction of ionized states, θ, derived from potentiometric titration data and a repeat experiment for the same PAHA polymer. See Figure S11 and SI Section 2.5.2.1 for the titration data analysis. The transition is markedly sharper than would be expected for a simple monoprotic acid. This is most apparent during the initial onset of the transition from low to high pH, as there is a clear tail to the curve in the latter half of the transition.

An important result derived from the model in the theoretical description of this system is that cooperative transitions for HPE present correlated conformational states and ionization transitions. It is evident from the curves that for our two-phase system, this is indeed the case. As the pH increases, we see a close correlation between the fraction of the polymer in the hydrophobic state and the ionization fraction of the polymer. Considering we do not expect significant ionization of the polymer in the pentanol phase, due to its much lower dielectric constant (∼15), a joint onset in both transitions is consistent with expectations. There are, however, clear deviations between the ionization and the hydrophobic fraction of the polymer at the higher pH side of the transition. A certain degree of difference between the curves is to be expected, as is shown in the graph through the predicted θ curve (light red dashed line), where we have input the fit parameters for eq 3 of the *f*_aq_ data into eq 4. The origin of this effect is that the p*K*_a_ of the acid groups (4.69 is estimated in this case) is fairly close to the transition-pH of the polymer, and therefore, the pH is too low to lead to full ionization when the polymer transitions. We hypothesize that the deviation between the number of ionizable groups and the measured degree of cooperativity *M* is mainly due to the presence of intermediate conformational states in the water phase that occur when we have intermediate ionization states. Similar deviations between the number of ionizable groups and *M* in transitions coupled to HPE ionization have been observed in micelle formation by diblocks containing an HPE block,^3,11^ and in the solubilization of bilayer vesicles.^3,13^ We therefore conclude that our simple oil–water partitioning setup reflects the behavior of (much) more complex systems where the HPE conformational (or ionization) state couples to structure and function.

As a comparison to our MWC approach, we also present a Hill plot for the partitioning transition (Figure 2c). We can transform the *f*_aq_ and θ data into Hill plot form, which gives rise to clear and aligned straight line sections. It is clear that these transitions present cooperativity, due to the large straight line section and larger than unity gradient (Hill parameter, *n*_H_). We have overlaid an *n*_H_ = 7.2 gradient line to show the consistency between the MWC fit and the Hill treatment. We find that the Hill plot, a commonly used “test” for cooperativity,^6,8,17–19^ does not afford as much further information as the MWC-like model we present here. Specifically, the merging of the free energy variables for the hydrophobicity and ionization within the Hill equation does not reflect the importance of the hydrophobic penalty in HPE transitions as a distinct variable to the ionization of the acid groups. Moreover, it does not allow for rationalizations on the difference between the fraction of polymer in the hydrophobic phase and the ionization state of the polymer, which occurs when the transition-pH is close to the p*K*_a_ of the acid groups.

#### Degree of Cooperativity in Binding of Iron Ligand onto Terpyridine Monomers and Terpyridine-Functionalized Polymers (OMC)

To investigate the degree of cooperativity in the binding of iron ligand onto Terpyridine oligomers, we again make use of a two-phase (with distinct hydrophobicities) experimental setup. Here we compare the partitioning of Terpyridine (abbreviated as T, which should not be confused with the Tense state) in a two-phase water/oil (dichloromethane, DCM) system, to that of a Terpyridine-functionalized oligomer with 16 units in length and a narrow polydispersity (DP = 16 and *Đ* ≈ 1.04, abbreviated as PT16). To synthesize PT16, first, poly­(*tert*-butyl acrylate) (P*t*BA) was synthesized by the SET-LRP method, then deprotected to poly­(acrylic acid) (PAA), and finally functionalized by Terpyridine groups. The two-phase setup was designed with equal volumes of the phases, over a wide range of initial concentration of iron ions in the aqueous phase (10–200 μM), and with a fixed concentration of Terpyridine groups (200 μM) in DCM (oil phase). Figure 3a shows an illustrative summary of the experiments. For the detailed description of synthesis steps and characterization of PT16, as well as the experimental details of the partitioning in the two-phase system, see Sections 3.2 and 3.3 of the SI, respectively.

**Figure 3:**
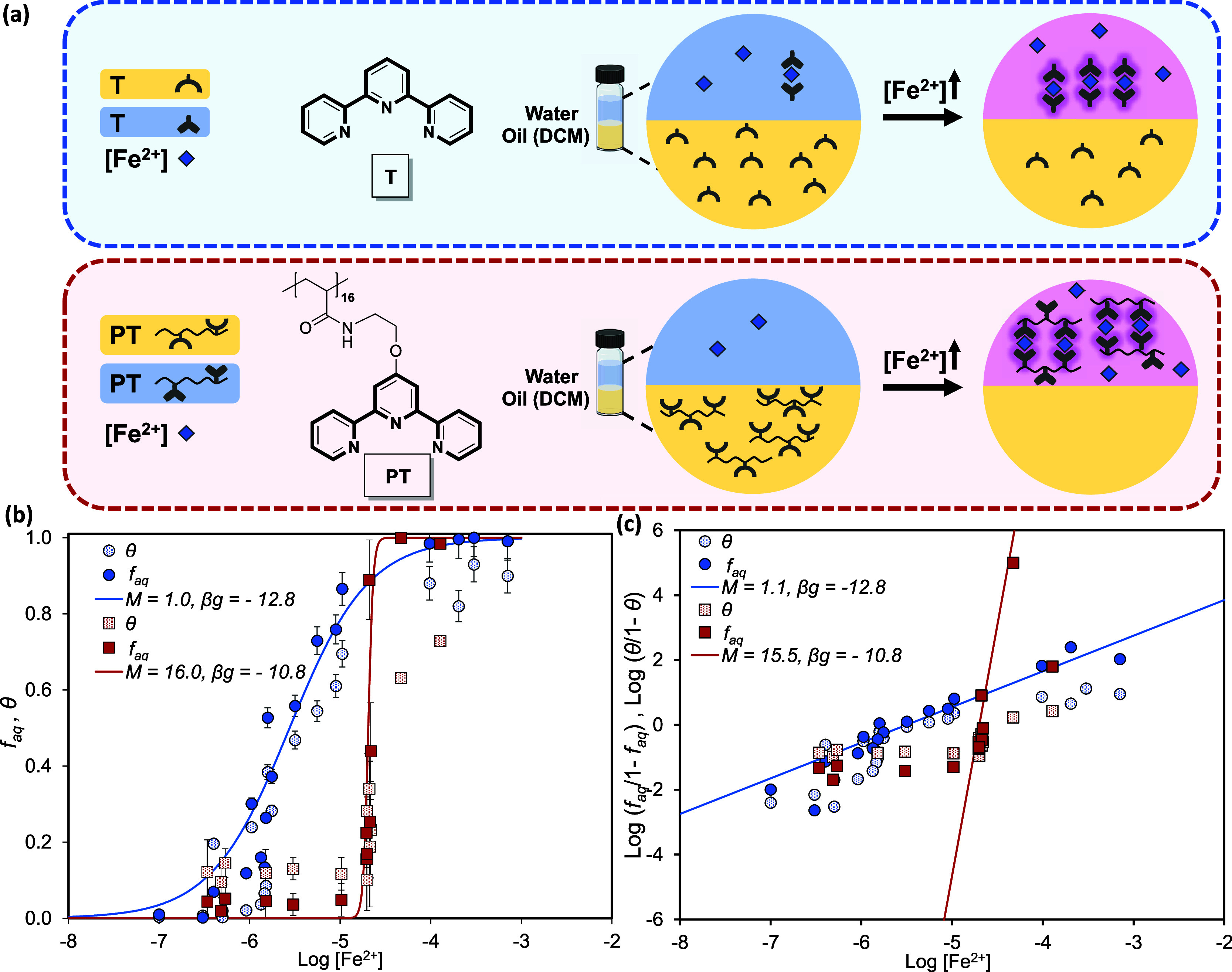
Terpyridine oligomers show much sharper, cooperative transitions than the corresponding monomer. (a) Schematic of the comparison of the partition experiments of monomeric (T) and oligomeric (PT) Terpyridine in oil (DCM) and water, with iron­(II) ions in the aqueous phase as “ligands.” (b) Fraction of Terpyridine monomers (filled blue circles) and oligomeric Terpyridine (filled red squares) in the aqueous state (*f*_aq_) as a function of the free iron concentration in the water phase. The blue solid line represents the best fit to Terpyridine to eqs 5 and 21 with β*g* = β­(2*g*_H_ + *g*_2_) = −12.8 and *M* = 1. The red line is the same equation with β*g* = β­(2*g*_H_ + *g*_2_) = −10.8 and *M* = 16, which describes well the experimental data on poly­(Terpyridine). The independently measured fraction of occupied Terpyridine sites (by iron­(II) ions) in the water phase, θ, is indicated by light blue circles for monomeric Terpyridine and light red squares for PT16. (c) The corresponding Hill plots for Terpyridine monomer and poly­(Terpyridine), where the blue and red lines represent Hill coefficients *M* equal to 1 and 16, respectively.

With a reported binding strength *g*_2_ ≈ −23 *k*_B_ *T* between iron­(II) ions and two Terpyridine binding sites^20^ (1:1 complexes of terpyridine and iron have not been observed^20,21^ ), and *K* = exp­(−β*g*_2_), for OMC with ligand concentration [Fe^2+^] ≳ 10^–9^ M, the general theoretical equations in the SI eqs 23 and 24 become, to an excellent approximation

$$f_{\mathrm{aq}}=\frac{(K_{p}K[{\mathrm{Fe}}^{2+}]{)}^{M}}{1+(K_{p}K[{\mathrm{Fe}}^{2+}]{)}^{M}}(\mathrm{OMC})$$ 5

and the total fraction of the occupied binding sites

$$\theta =\frac{K[{\mathrm{Fe}}^{2+}]}{1+K[{\mathrm{Fe}}^{2+}]}f_{\mathrm{aq}}\approx f_{\mathrm{aq}}(\mathrm{OMC})$$ 6

In these equations, *K*_p_ = exp­(−2β*g*_H_) with *g*_H_ the reversible work to transfer a Terpyridine moiety from the oil phase to the aqueous phase, analogous to a conformational penalty in the aqueous phase. Unlike for the HPE case, it is nontrivial to link the precise value of *M* to the number of Terpyridine groups per oligomer, as the aqueous state of the OMC is in fact a cross-linked (by iron ions) gel of oligomers, see Figure S24 in the SI. Since iron ions are bound by two Terpyridine residues, formation of each Terpyridine–iron complex leads to a binding free energy *g*_2,_ accompanied by a hydrophobic penalty 2*g*_H_. We note that eqs 5 and 6 are manifestations of the Hill equation, eq 1, reflecting a limiting case where oligomers are either fully bound or unbound to iron­(II) ions. Eqs 5 and 6 depend on the combined quantity *K*_p_ *K* = exp­(−β­(2*g*_H_ + *g*_2_)). We fit our experimental data to eqs 5 and 6 for both Terpyridine monomers as well as for oligomers. The value of *g*_H_ for Terpyridine monomers has been determined by the solubility in water and in DCM, see SI Section 3.4.

The binding behavior of iron ligands onto Terpyridine groups was investigated by quantification of free iron and Terpyridine concentrations in the water and oil phases using UV–vis and ICP-AES, to obtain the free ligand (iron) concentration, fraction of occupied binding sites, and fraction of Terpyridine groups transitioned from DCM to the water phase. The details of the quantification procedure are described in Sections 3.3.1 and 3.3.2 of the SI, with the associated data shown in Figures S23 and S24 for Terpyridine monomers and the Terpyridine-functionalized oligomer, respectively.

The fraction in the aqueous phase and fraction of bound (to iron­(II) ions) Terpyridine monomers and oligomers are shown in the form of *f*_aq_ and θ as a function of free iron concentration in Figure 3b. A traditional Hill plot is shown in Figure 3c as a comparison. The experimental data were fitted to eqs 5 and 6, shown by solid lines in Figure 3b. Compared to the monomers, the Terpyridine-functionalized oligomers show (1) a significantly sharper transition (over only a narrow range of free iron concentrations) and (2) at higher free ligand (iron) concentration, where the transition occurs, see the squares versus circles, respectively, in Figure 3b. The first observation is consistent with the results for the HPE in the previous section, with a significantly larger degree of cooperativity for the oligomers *M* ≈ 16 compared to the monomers with *M* ≈ 1. The second observation points to a smaller negative value of 2*g*_H_ + *g*_2_ for the oligomers compared to the monomers: eqs 5 and 6 predict equal values for the transition defined by *f*_aq_ = θ = 1/2 at equal 2*g*_H_ + *g*_2_, independent of the value of *M*. From the fits we obtain β­(2*g*_H_ + *g*_2_) ≈ −12.8 for the monomers, and β­(2*g*_H_ + *g*_2_) ≈ −10.8 for the oligomers. From the solubilities of Terpyridine monomers in (acidified) water and in DCM, see SI Section 3.4, we find β*g*_H_ ≈ 6.8, which leads to β*g*_2_ ≈ −26.4, for the monomers, being comparable to the value of β*g*_2_ = −23 reported previously^20^ for monomeric Terpyridine in (neutral) water. The slightly lower negative value of β­(2*g*_H_ + *g*_2_) ≈ −10.8 for the oligomers could be caused by an additional hydrophobic contribution to *g*_H_ by the oligomeric backbone, or by a decreased (less negative) iron-Terpyridine binding strength *g*_2_ due to Coulomb repulsion of neighboring bound iron­(II) ions, or both. We conclude that the value of 2*g*_H_ + *g*_2_ extracted by comparing the experimental data to eqs 5 and 6 is consistent with independently obtained values of *g*_H_ (by solubility measurements) and of *g*_2_ (in ref 20).

As pointed out in Section 3.7 in the SI, for the binding of two Terpyridine monomers onto iron­(II) ions, it is expected that *M* ≈ 1/2, see eq 15 in the SI. However, the experimental data are not consistent with that scenario, see Figure S25 in Section 3.7 of the SI. Eqs 5 and 6 for *M* = 1 is analogous to iron­(II) ion binding units in the form of Terpyridine dimers, which is consistent with the experimental data as well as the value of 2*g*_H_ + *g*_2_ extracted from independent sources. This observation implies that Terpyridine forms dimers in DCM, even in the absence of iron ions. If this behavior is general and also occurs in other types of oils it is unclear at this point.

For the oligomers, as pointed out in the introduction of this section, the value of *M* cannot trivially be linked to the number of Terpyridine residues per oligomer. There, cross-linked, gel-like networks form in the aqueous phase where bonds between two Terpyridine sites onto different oligomers and iron ions form. Similar to the situation for the Terpyridine monomers, we expect small clusters of oligomers due to Terpyridine dimerization to form in the oil phase. The value of *M* ≈ 16 that we find for PT16 points to clusters of, on average, two oligomers. Obviously, further network formation mediated by iron­(II) ions occurs in the aqueous phase.

We observed that the gels in the aqueous phase (see Figure S24) can easily be ripped apart with tweezers. Upon gentle shaking, the gel fragments rapidly ‘heal’ to a single blob. In contrast, upon diluting the aqueous phase below the iron­(II) concentration where the gels are stable, slow (over the course of weeks) disappearance of the gels is observed. These findings indicate that the preferred conformational state of the oligomers as a function of ligand concentration can translate into functional behavior: the aqueous gel state can be sharply switched between “self-healing” and “self-destructing” as a function of the ligand concentration.

#### Partitioning Behavior of a Compositionally Disperse Copolymer

We now present the partitioning results for a copolymer HPE, poly­(*n*-butyl acrylate-*r*-acrylic acid) (PBA-AA). As detailed in the SI Section 2.4.5, this polymer is synthesized from a 1:1 ratio of *n*-butyl acrylate and *t*-butyl acrylate. These two monomers present very similar reactivity in a (controlled) radical polymerization; therefore, we can assume that their distribution within a polymer chain is random.^22^ The selective removal of the *t*-butyl group leads to a random distribution in the ratio of hydrophobic (*n*-butyl acrylate) and ionizable (acrylic acid) groups per chain throughout the HPE sample. This will lead to an ensemble of polymers with spread values of ionizable groups (*M*), which will vary the total ionization energy per chain and, likewise, a spread of hydrophobic penalty values (*g*_H_).

In the SI (Section 1.3), we derive the analogs for the fraction of HPE in the aqueous state (*f*_aq_, eq 3) as well as the fraction of ionized carboxyl groups θ, eq 4, for HPE with a binomial distribution of ionizable groups and hydrophobic groups. The analysis includes composition dispersity as well as length dispersity, in the form of a log-normal distribution. In short, we consider the transitions for each set of values of ionizable groups and total length, and then apply the weights from the corresponding distributions to them. The inclusion of composition dispersity is an important test case for the theory that we developed. Moreover, controlled composition dispersity in combination with fractionation opens up a new methodology to tune the pH at which the hydrophobic–aqueous transition occurs, as well as the width of the transition. We will follow up on this in the next section.

The data were acquired through a buffered partitioning experiment (pH 3–11) using the coumarin-modified poly­(*n*-butyl acrylate-*r*-acrylic acid) copolymer PBA-AA^*c*^, of around 20 units in length (DP= 22, *Đ* = 1.1), in a two-phase pentanol–water system as described in the SI (Section 2.3.1). The coumarin dye is added to the chain-end of these polymers to allow for UV–vis spectrometry to be used to track their concentration, as was done earlier for the PAHA system. The *f*_aq_ data were derived from the UV–vis absorbance (320 nm) of the coumarin dye in each pentanol solution, for two individual runs, compared to the stock solution (0.5 mg/mL). Figure 4a presents the fraction of the polymer in the aqueous phase, *f*_aq_, of the two-phase system. Figure S12 and SI Section 2.5.1.2 show the UV–vis absorption curves and explain the data treatment employed to extract the curve. In stark contrast to the results in Figure 2 for the PAHA HPE, the transition is spread out over a wide pH range, around 4 units. We may refer to this, from a purely phenomenological perspective, as noncooperative behavior. However, we expect each individual polymer chain to behave cooperatively, but the chemical dispersity between the different chains leads to a spread in transition-pH values across the polymer sample and therefore leads to an overall broad transition.

**Figure 4:**
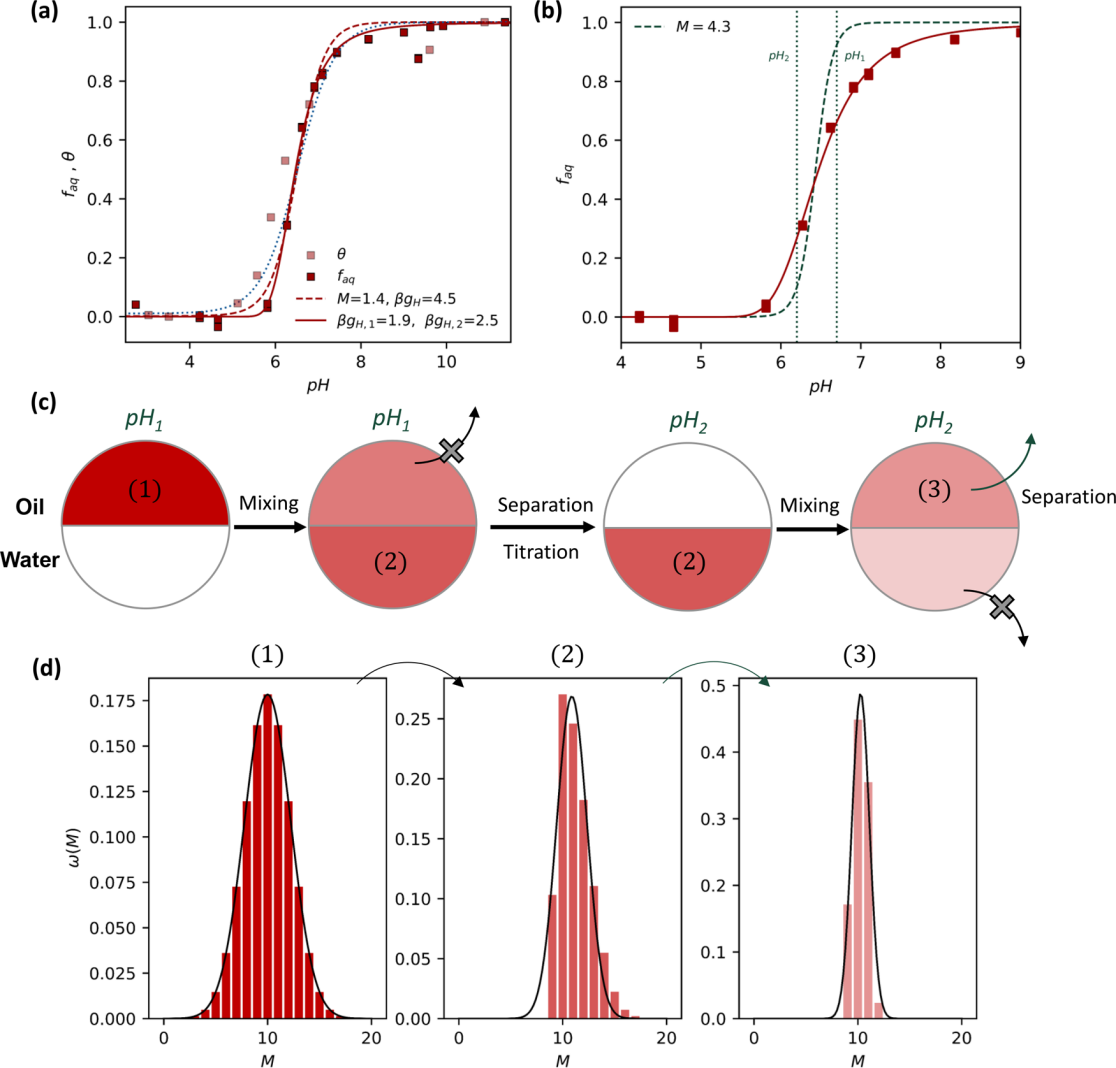
Chemically disperse copolymeric HPE presents broad, noncooperative partitioning transitions. Fractionation can return sharp transitions. (a) Plot of *f*_aq_ (red squares) and θ (light red squares) for PBA-AA. The data for each variable were independently collected from separate experimental systems. Repeat measurements of *f*_aq_ are individually plotted to illustrate the experimental spread of the data. The curves are a fit using eq 3 (dashed red) and SI eq 28 (solid red), using the p*K*_a_ values for acetic acid: 4.56.^16^ Fit parameters are shown in the legend. As a comparison, a (calculated) noncooperative transition with *M* = 1 is plotted as a dotted blue line. (b) *f*_aq_ for the PBA-AA system and its corresponding fit (SI eq 28) are shown as red squares and a red line. Note the asymmetric shape of the curve around the transition point (*f*_aq_ = 0.5). The green dashed line shows a calculated *f*_aq_ transition from the resulting HPE sample after the fractionation procedure (shown in (c)). (c) Schematic for a potential fractionation procedure using a two-phase oil and water system. After equilibration at each fractionation pH point, one of the phases is discarded (marked as a gray cross beside an arrow). Shades of red within a phase reflect polymer concentration. pH_1_ > pH_2_ . (d) Distribution of the number of ionizable sites (*M*) on the polymer chains at different stages of the fractionation procedure. Only the distribution for the chains with 20 total monomers (*M*_t_) is shown. Gaussian curves are plotted to guide the eye. See SI Sections 2.5 and 1.4 for details on data treatment and scaling, and fractionation calculations, respectively.

Using eq 3, we can fit the transition with our nondisperse model as a comparison and extract a value for the effective value of *M*, which yields 1.40. Therefore, at least phenomenologically, the ensemble of chains behaves fairly noncooperatively. This symmetric fitted curve (around *f*_H_ = 0.5) does not match the asymmetric experimental transition well. The transition flattens out at the higher *f*_aq_ values, confirming the expected asymmetry found in the numerical predictions in the SI (Section 1.3).

Using SI eq 28, we can fit the transition, taking into account the known chemically dispersed nature of the polymer. We assume a binomially distributed pair of monomers, use the expected value of the dispersity (1.04) (see SI Section 2.4 for a discussion on the dispersity value used), and set an average total length of 20. We then fit the distribution with two free parameters, namely the hydrophobic penalty for each of the monomers. With values of *g*_H,1_ = 1.94, *g*_H,2_ = 2.48 for the acrylic acid (*g*_H,1_) and the *n*-butyl acrylate (*g*_H,2_) groups, a good match between the expected transition predicted by the numerical model and the experimental system is obtained. We would indeed expect the *n*-butyl acrylate groups to have a larger hydrophobic penalty than the acrylic acid groups.

We, again, extract the fraction of ionized groups on the polymer from a potentiometric titration experiment of the two-phase system and the similar polymer PBA-AA^0^ (DP = 21, *Đ* = 1.1) (see SI Section 2 for synthesis details). This polymer sample was not modified with coumarin. The resulting data (see SI Section 2.5.2.1 and Figure S12) is plotted in light red in Figure 4a. Compared to what was seen for the homopolymer HPE, the degree of correlation between these two transitions is not as high. A slight offset between them is predicted and explained in the theoretical section, but the experimental results do not match this behavior. It is possible that the addition of the coumarin dye for the partitioning data (*f*_aq_) increases the hydrophobicity of the polymer, shifting the transition to a higher pH value. Moreover, the ionization fraction and hydrophobic fraction data were acquired from independent unbuffered and buffered experiments, respectively. It is possible that there is an offset in the pH calibration between the experiments. There is also a lack of data points in the titration data at higher pH values, which hinders any detailed discussion about the difference between the curves.

Overall, there is good agreement between the predictions for a binomially distributed binary copolymer and the experimental data presented here. The broadening effect is substantial, and therefore, the monomer composition dispersity of copolymers is an important design aspect to be considered when designing polymers to present a particular pH response.

Due to the direct relationship between the comonomer dispersity in the polymer and the theoretical framework, we can predict the transitions for a variety of different copolymers with different monomer distributions. This finding is potentially relevant to applications involving (random) copolymers of styrene and maleic acid (SMA), broadly used in isolating membrane proteins by bilayer disk formation.^23–26^ There, a broad transition may be beneficial, with applications over a relatively wide (several units) pH range. Moreover, we may be able to leverage the partitioning of the different chains to create polymer samples that transition at a particular pH value and sharpness.

#### Fractionation of Compositionally Disperse Copolymers

As detailed above, polymers presenting chemical dispersity between the chains in a single polymer sample present broader transitions than homopolymers. However, the transitions of each individual chain are still expected to be as sharp as a similar homopolymer. If we were able to selectively fractionate only a specific subset of the chains in a composition-dispersed polymer sample, this subset may present sharp transitions.

Such an approach may also provide a solution for the difficulty of targeting a specific transition-pH using homopolymers. If restricted to only commercially available monomers, it can be challenging to synthesize an HPE with a specific hydrophobic penalty value. The fractionation approach would start with a polymer sample with a broad comonomer distribution, leading to a broad transition, and from it extract a fraction of chains with the desired transition-pH. This would be much less atom-efficient, but may give access to both tailored transition-pH values and transition broadness that may be harder to access from direct polymer synthesis. In this section, we will focus on the fractionation of an HPE with a disperse ratio of ionizable groups (*M*) to total monomers (*M*_t_). An example of this class of polymer is the poly­(*n*-butyl acrylate-*r*-acrylic acid) (PBA-AA) HPE examined above, whose broad oil–water partitioning transition is the basis of the following discussion.

A schematic illustrating a simplified experimental procedure to potentially achieve this is shown in Figure 4c. We postulate the use of a two-phase oil and water system to carry out this fractionation. By carrying out two separate partitioning steps of the polymer sample at different pH values, we can isolate a fraction of the polymer that transitions between the two chosen pH values. Figure 4d graphically describes the changes to the width of the chemical dispersity distribution during the fractionation procedure. After the first separation step, one of the edges of the distribution is removed; in this case, the most hydrophobic chains remain in the oil phase, which is then discarded. The second step at a lower pH value removes the most hydrophilic chains from the other edge of the distribution. The final sample remains in the oil phase of the second step and presents a much narrower chemical dispersity.

The specific transition that the fractionated sample will now exhibit depends on the specific shape of the length and comonomer ratio distribution of the initial sample. If the average value of *M* for the fractionated chains is large (*M* > 10), then the bulk of the transition for the fractionated polymer will occur within the two fractionation pH values chosen.

Assuming the polymerization of a particular polymer sample is well understood and its length dispersity is measured, a good estimation of the relative weights for the different chain configurations (number of repeating units of each type) can be made. A measurement of the transition of the whole polymer sample between the phases of the oil–water system (shown in Figure 4a) allows us to fit the hydrophobic penalty parameter for the different (*M*, *M*_t_) configurations. This then allows for a prediction of exactly what chains transition at which pH.

As shown in Figure 4b, it is possible to extract a copolymer sample from an initial disperse copolymer sample that transitions over 0.5 pH units. In the case of the fractionation procedure leading to the blue line in this figure, the predicted ratio of chains that remain from the initial sample is 39%. Details on the numerical procedure to predict the transition curve of the fractionated polymer are presented in the SI Section 1.4.

## Conclusions

In the present work, we pinned down the conditions for cooperative LMT and their underlying mechanism. We showed that in two very different and relatively simple macromolecules, coupling between conformational states and ligand binding leads to strongly cooperative transitions in oligomers with 16–20 ligand binding sites. As a comparison, no cooperativity has been observed in monomeric analogs under the same conditions. The key is to select two well-defined conformational states: a ground state with low ligand affinity, and a conformationally unfavorable state, yet with relatively high ligand affinity. These two states have been realized by demixed oil–water systems, where, in both studies, the state with oligomers dissolved in oil represents the ground state. Because of the hydrophobic nature of the oligomers, dissolution in water constitutes an unfavorable state quantified by a conformational penalty. However, the aqueous state can be stabilized by ligand binding. If the binding of several ligands is required to overcome the unfavorable interactions with water, the transition is cooperative and presents a sharp response to the ligand concentration in the system.

We have proved this scenario for HPE and OMC. Several earlier observations of cooperative transitions exist where HPE are involved. In these studies, the HPE is either one of the blocks in diblock copolymers,^11,18^ is part of a cross-linked network,^27,28^ or the transition is coupled to lipid bilayer solubilization.^14,29^ In these systems, the conformational states of the HPE are well-defined, and couple to structure and function: the formation or dissolution of pH-dependent micelles in HPE containing diblocks,^18^ pH-dependent solubilization of lipid bilayer membranes,^29^ and swollen/collapsed states of cross-linked HPE.^27,28^ In our work, we reduced complexity to the essentials: single-chain HPE with conformational states defined by a hydrophobic and an aqueous reservoir. In that way, we have been able to prove directly the coupling between conformational states and ligand binding, an essential feature predicted by MWC theory,^1,3^ see Figure 1. Moreover, we showed that monomer compositional dispersity dramatically influences the width of the conformational transition for binary copolymers, which is potentially relevant in their applications.^14^ Fractionation of compositional disperse HPE in principle provides a way to select desired pH ranges where the transition occurs “to order.”

As far as we are aware, we provide the first observation of a cooperative transition in OMC. The essential difference with HPE is that here, iron­(II) ions form bonds with two terpyridine residues on the oligomers. We find a strongly cooperative transition as a function of free iron­(II) ion concentration, where the OMC moves from a dispersed state in oil to a gel in the aqueous phase. Even in this simple setup, coupling of the conformational state to structure and function is apparent: gels form and self-heal (after being ripped apart) rapidly beyond a critical iron­(II) ion concentration, and “self-destroy” below that concentration. The gels will only slowly disappear upon diluting the water phase below the critical iron ion concentration. These findings open up potential for “self-healing” and slow-release systems triggered by very small variations in ligand concentration.

The general framework of LMT can, in principle, be applied to a wide range of ligand-polymer systems, as long as the systems present a constrained number of conformations for the polymer chains. Through the use of a simple two-phase oil and water system, we have shown that MWC-like cooperative transitions can occur in relatively simple “nonbiological” macromolecules with a variety of ligands.

## Supplementary Material



## Notes

General theoretical background (SI-1); hydrophobic polyelectrolytes (HPE, SI-2); oligomeric metal chelator (OMC, SI-3) (PDF)

## §

University Medical Centre Utrecht, Utrecht 3584 CT, The Netherlands

## ∥

Eindhoven University of Technology, Eindhoven 5600 MB, The Netherlands.

## ⊥

Allnex Netherlands BV, Synthesebaan 1, Bergen op Zoom 4612 RB, The Netherlands.

## #

Utrecht University, Utrecht 3584 CG, The Netherlands.

## ∇

TNO Environmental Modeling, Princetonlaan 6, Utrecht 3584 CB, The Netherlands.
