# Supplementary material for: Cooperative Ligand-Mediated Transitions in Simple Macromolecules
Source: J Phys Chem B. 2025 Oct 28;129(44):11594–604. doi: 10.1021/acs.jpcb.5c05386 (PMC12598847; doi:10.1021/acs.jpcb.5c05386)
Supplement: Supplementary file 1 [file jp5c05386_si_001.pdf]

Supporting Information: Cooperative Ligand-Mediated Transitions in Simple Macromolecules

James L. Martin Robinson*,[a,†] Neshat Moslehi*,[b,†] Nikolaos Dramountanis ,[c,†] Lennart van den Hoven,[d,†] Alexander M. van Silfhout,[†] Kanvaly S. Lacina,[†] Mies van Steenbergen,[‡] Wessel Custers,[†] Bas G. P. van Ravensteijn,[‡] and Willem K. Kegel[*,†]

† *Van't Hoff Laboratory for Physical and Colloid Chemistry, Debye Institute for Nanomaterials Science, Utrecht University, 3584 CH Utrecht, The Netherlands*

‡ *Department of Pharmaceutics, Utrecht Institute for Pharmaceutical Sciences (UIPS), Utrecht University, Universiteitsweg 99, 3584 CG, Utrecht, the Netherlands*

E-mail: w.k.kegel@uu.nl

*J.L.M.R. and N.M. contributed equally to this work.

[a]Present address: Department of Orthopedics, University Medical Centre Utrecht, 3584 CT Utrecht, The Netherlands

[b]Present address: Laboratory of Self-Organizing SoftMatter, Department of Chemical Engineering and Chemistry, Eindhoven University of Technology, 5612AZ Eindhoven, The Netherlands

[c]Present address: Allnex Netherlands BV, 4600 AB, Bergen op Zoom, The Netherlands

[d]Present address: Department of Pharmaceutics, Utrecht Institute for Pharmaceutical Sciences (UIPS), Utrecht University, Universiteitsweg 99, 3584 CG, Utrecht, the Netherlands

Contents

1 General theoretical background **S5**

1.1 Hydrophobic polyelectrolytes (HPE) S9

1.2 Terpyridine monomers and terpyridine-functionalized polymers (OMC) . . S10

1.2.1 Terpyridine monomer S11

1.2.2 Terpyridine-functionalized oligomers (OMC). S16

1.3 Composition dispersity in HPE copolymers S17

1.4 Fractionation calculation S22

2 Hydrophobic polyelectrolytes (HPE) **S24**

2.1 Monoprotic acids S24

2.2 Materials and instrumentation S24

2.2.1 Materials S24

2.2.2 Instrumentation S26

2.3 Experimental procedure S27

2.3.1 Buffered system S29

2.3.2 Unbuffered system (titration) S30

2.4 Polymer synthesis and characterization S31

2.4.1 poly(6-(acryloyl)aminohexanoic acid) (PAHA) S31

2.4.2 Synthesis of 6-(acryloyl)aminohexanoic acid S31

2.4.3 RAFT polymerization of 6-(acryloyl)aminohexanoic acid S32

2.4.4 PAHA polymer characterization S34

2.4.5 poly(n-butyl acrylate-*s*-acrylic acid) (PBA-AA) S38

2.4.6 Synthesis of a *clickable* 4MU dye S39

2.4.7 SET-LRP synthesis of poly(n-butyl acrylate-*s*-t-butyl acrylate) . . . S39

2.4.8 Azidification of poly(n-butyl acrylate-*s*-t-butyl acrylate) S41

2.4.9 Coumarin dye - polymer chain end *click* reaction S42
2.4.10 poly(n-butyl acrylate-*s*-t-butyl acrylate) deprotection S43
2.4.11 PBA-AA polymer characterization S45
2.5 Measurement of HPE partitioning and ionization behavior S46
2.5.1 Measuring the fraction of chains in the hydrophobic phase S47
2.5.1.1 PAHA (DP=18): f_H from titration experiment S47
2.5.1.2 PBA-AA^c: f_H from buffered experiment S49
2.5.2 Measuring the HPE ionization fraction from titration experiments . S49
2.5.2.1 PAHA (DP= 18): θ from titration data S50
2.5.2.2 PBA-AA^0: titration and buffered experiments S51

3 Oligomeric metal chelates (OMC) S53
3.1 Materials S53
3.2 Synthesis of the terpyridine-functionalized polymer (PT) S54
3.2.1 Synthesis of 2-(2,2':6',2"-terpyridine-4'-yloxy) ethylamine (ET) (1) . S55
3.2.2 SET-LRP Synthesis of poly(*tert*-butyl acrylate) (P*t*BA) (2) S57
3.2.3 Deprotection of (2) into poly(acrylic acid) (PAA) (3) S59
3.2.4 EDC/NHS Activation of (3) into PNHS (4) S59
3.2.5 Functionalization of PNHS (4) with ET (1) to get PT16 (5) S60
3.3 Monitoring the binding behavior of iron onto terpyridine in a two-phase water and oil set-up S61
3.3.1 Quantification of the free iron concentration in the water phase by inductively coupled plasma - atomic emission spectroscopy (ICP-AES) and ultraviolet-visible light spectroscopy (UV-Vis) S62
3.3.2 Quantification of terpyridine/terpyridine-functionalized polymers in the oil phase by ultraviolet-visible light spectroscopy (UV-Vis) . . . S63

3.4 Determination of the hydrophobic contribution g_H of terpyridine by its solubility in DCM and water S64
3.5 Partitioning of terpyridine monomer in the two-phase system S65
3.6 Partitioning of the terpyridine-functionalized polymer in the two-phase system S66
3.7 Terpyridine monomer partitioning between DCM and water S67

References **S68**

1 General theoretical background

Here we summarize the model used in the main paper and in the next section we generalize it to include multivalent ligands and composition dispersity.

We use a grand canonical ensemble treatment to describe our systems, that is, we take the system as open to the exchange of ligands. The grand canonical statistical weights of a polymeric template with M binding sites in a hydrophobic (or 'oily') (H) and an aqueous (aq) environment, respectively, are:

$$\Xi_H = (1 + \lambda \exp(-\beta g'))^M \approx 1. \tag{1}$$

$$\Xi_{aq} = \exp(-\beta G)(1 + \lambda \exp(-\beta g))^M . \tag{2}$$

Where the Ξ_i are the coarse-grand partition functions of the $i = H, aq$ state of a template, λ is the fugacity of the ligand, $\lambda = \exp(\beta\mu)$, with $\beta = 1/k_B T$. k_B is Boltzmann's constant and T the absolute temperature. μ is the chemical potential of the ligand that adsorbs (or binds) onto the macromolecular template. μ is related to the ligand concentration or partial pressure of the ligand. g is the binding free energy of the ligand to a binding site on a chain within the aqueous phase, and g' the binding free energy of a ligand onto the template in the hydrophobic state. G represents the reversible work required to transfer a chain from its hydrophobic state in the oil phase to the aqueous phase, and takes into account the (unfavorable) interactions between hydrophobic moieties on the macromolecules and water. This term is analogous to the self-energy term of the hemoglobin molecule when changing conformations upon adsorption of oxygen ligand. We have set $\Xi_H \approx 1$ as binding of the ligands to the templates in the hydrophobic state is unfavorable and therefore $\lambda \exp(-\beta g') << 1$. In the case of hemoglobin, with $M = 4$, the analogues of the hydrophobic and aqueous states are the T and the R states, respectively, and both

states have finite affinity for the oxygen ligands.

The expression for the grand partition functions can be derived in the usual way by writing $\Xi = \sum_{N=0}^{M} Z(N,M,T)\ \lambda^N$. Here, subscripts have been dropped. The (coarse-grained) canonical partition function is given by $Z(N,M,T) = \exp(-\beta G)\ \Omega(N,M)\ \exp(-\beta N g)$ with $G = 0$ for a template in the hydrophobic state. Under the assumption that binding sites are uncorrelated, the number of ways N ligands can be distributed over M binding sites is $\Omega = \binom{M}{N}$, which has been used to derive Eqs. (1, 2) by making use of the binomial theorem $\sum_{N=0}^{M} \binom{M}{N} x^N = (1+x)^M$. For the terpyridine oligomers with iron ions as ligands, the situation is more complicated as will be discussed in the section on terpyridine-functionalized templates below.

From Eqs. (1, 2) the fraction of occupied binding sites (by the ligand), θ, and the fraction of polymer chains in each of the phases can be derived. For the polymer fractions (when considering equal volumes), we have $f_i = \Xi_i/\Xi$, where the subscript i can be hydrophobic (H) or aqueous (aq), and Ξ is the total partition function , $\Xi = \Xi_{aq} + \Xi_H$. For our two-phase oil and water system we have

$$f_H = \frac{1}{1 + \exp(-\beta G)(1 + \lambda \exp(-\beta g))^M} \tag{3}$$

$$f_{aq} = 1 - f_H. \tag{4}$$

Figure S1(a) shows the effect of the value of M on the sharpness of the transition, and (b) illustrates the effect of the hydrophobic penalty G, for a fixed binding energy g. Theoretical framework therefore predicts that oligomeric species are long enough to present sharp, cooperative transitions. As the hydrophobic penalty increases the transition is shifted towards higher fugacity values, as more favorable binding in the aqueous must counteract an increase in the stability of the template in the hydrophobic phase.

The fraction of occupied binding sites is given by,

$$\theta = \frac{\langle N \rangle}{M} = \frac{1}{M}\frac{\lambda}{\Xi}\frac{\partial \Xi}{\partial \lambda} = \frac{\lambda \exp(-\beta g)}{1 + \lambda \exp(-\beta g)} f_{aq}, \tag{5}$$

Note the correlation between the fraction of occupied binding sites θ and the fraction of chains in the aqueous phase, f_{aq} being analogous to the situation for hemoglobin in Fig. 1(a) where θ is strongly correlated with f_R. This general model can then be applied to a particular ligand-polymer system by finding a suitable expression for the fugacity (λ).

In the situation that we have only a single conformational state, so that one of the partition functions in Eqs. (1, 2) equals zero, we immediately recover the Langmuir adsorption equation. For example if we take $\Xi_H = 0$, we find by using the left-hand side of Eq (5)

$$\theta_L = \frac{\lambda \exp(-\beta g)}{1 + \lambda \exp(-\beta g)}, \tag{6}$$

which has been plotted in Fig. 1.

It is important to note that the equations above represent an idealized case of a co-operative binding-conformation transition for a template with multiple (M) binding sites for ligands. The model assumes that the template or receptor only has two stable conformational states throughout the transition. While this is plausible for a highly constrained biological molecule such as hemoglobin, it may be unrealistic for relatively simple macromolecules. The presence of intermediate states between the two dominant conformational states will lead to a broadening of the curve for the fraction of occupied sites and the transition overall. Due to the predicted correlation between the value of M and the sharpness of the transition this can be considered as a reduction in the effective value of M. Therefore, M, although directly correlated to the number of binding sites, should be seen as a cooperativity parameter. The closer the M value derived from an experimental transition is to the physical number of binding sites on a template, the closer the system abides to a

purely two-state transition.

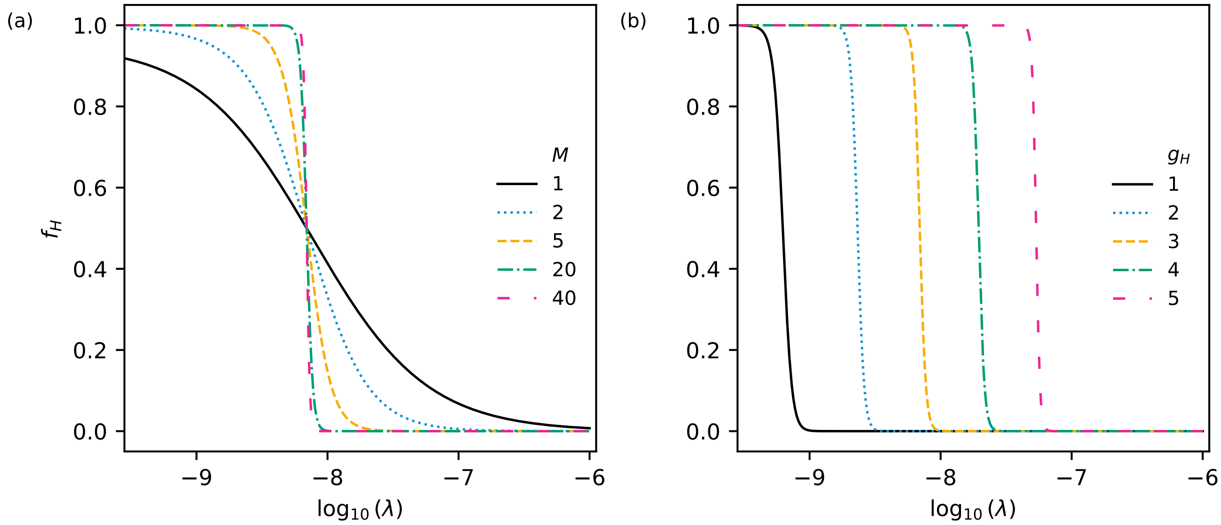

Figure S1: **General LMT two-state transition behavior.** **(a)** Increasing the value of M in Eq. (3) leads to an increase in sharpness of the transition. M can be considered a cooperativity parameter. **(b)** The parameter g_H, defined as $\beta G = M g_H$ (Eq. (3)) , affects the value of the fugacity at which the transition occurs.

Additionally, in this analysis, we have assumed that the binding energy for every binding site is equal and that there are no interactions between the binding sites. Considering that the binding of charged ligands will lead to the ionization of the site and electrostatic repulsion with neighboring ionized binding sites, this cannot be fully neglected. It is not within the scope of this work to detail the effects of intra-template interactions, but we note that these interactions will lead to a spread in the binding site energy and therefore a broadening of the transition, in fact negative cooperativity. This effect is reflected in a smaller value of M as expected from the number of ligand binding sites.

There may also be interactions that lead to a larger value of M than expected from the architecture of the polymer template, as will be seen in the situation for the terpyridine OMC system. There, a large value of M can a priori be attributed to the high configurational entropy of the ligands and the templates as reflected in the value of the multiplicity $\Omega(N, M)$ as discussed above. Overall the value of M gives us information about the be-

havior of the system compared to an ideal two-state, uncorrelated reference situation.

In ending this section, we point out the condition where the so-called Hill equation[S1–S3] is recovered.

Taking $\lambda \exp(-\beta g) >> 1$ and writing $G = M g_H$, where g_H should not be confused with g'(binding free energy of a ligand onto the template in the hydrophobic state) in Eq. (1), we obtain

$$\theta = \frac{(\lambda \exp(-\beta(g+g_H)))^M}{1+(\lambda \exp(-\beta(g+g_H)))^M} = \frac{(K_a[L])^{n_H}}{1+(K_a[L])^{n_H}} \quad (= f_{aq}). \tag{7}$$

In these conditions the variable M is the 'Hill exponent' (n_H). In general, $\lambda \exp(-\beta(g+g_H)) = [L]K_a$ with $[L]$ the unbound ligand concentration and K_a the (effective) association constant. This is commonly used to extract the value of the Hill exponent (in this case M) by plotting $\log \frac{\theta}{1-\theta}$ versus $\log [L]$.

Compared to a strictly empirical value such as the Hill constant, we note that the value of M is directly linked to the number of ionizable groups of the template and therefore the theory allows for the effects of polymer length and length (size) dispersity, as well as chemical dispersity to be predicted. The number of ionizable sites serves as an upper limit to the sharpness of a transition, and the deviation from this upper limit of M also holds information about the system.

1.1 Hydrophobic polyelectrolytes (HPE)

In this section we extend the general theoretical description to HPEs. As shown in a previous publication[S4], for HPE with M weakly acidic ionizable groups, we have $\lambda \exp(-\beta g) = 10^{pH-pKa}$ so that Eq. (2) can be written as

$$\Xi_{aq} = \exp(-\beta G)\left(1+10^{pH-pK_a}\right)^M. \tag{8}$$

Where the pK_a is the negative logarithm of the acid dissociation constant of the ionizable groups on the HPE and the pH is the negative logarithm of the proton concentration in the aqueous phase of the system. Here we have assumed all structural ionizable sites (M), that is the physical number of ionizable sites on the chain, have the same ionization constant. Similarly we may express f_H and f_{aq} as

$$f_H = \frac{1}{1 + \left(\exp\left(-\beta G\right)\left(1 + 10^{pH - pK_a}\right)^M\right)} \tag{9}$$

$$f_{aq} = 1 - f_H. \tag{10}$$

The fraction of occupied binding sites is then:

$$\theta = \frac{10^{pH - pK_a}}{1 + 10^{pH - pK_a}} f_{aq}. \tag{11}$$

The correlation between the ionized fraction and fraction of HPE in the aqueous state is strongest when the exponent $pH - pK_a$ is large compared to unity. This corresponds to a situation where $pH_{trans} > pK_a$ by around a couple of pH units. The transition-pH, pH_{trans}, is defined here as the midpoint of the transition, where $\Xi_H = \Xi_{aq} = 1$.

1.2 Terpyridine monomers and terpyridine-functionalized polymers (OMC)

Terpyridine is an extensively-used chelating group, well-known for its high affinity towards complexation with many transition metal ions including iron. While terpyridine can form both mono and bis complexes with iron(II), $(FeT)^{2+}$ and $(FeT_2)^{2+}$, respectively, the formation of bis complexes is thermodynamically favorable[S5]. Therefore, the mono complexes are neglected and we assume that iron ions always bind two terpyridine binding sites, where the

binding sites can either be two terpyridine monomers located on different oligomeric templates (inter-template bond) or onto the same oligomeric template (intra-template bond).

1.2.1 Terpyridine monomer

Here we first apply chemical equilibrium conditions to the partitioning of terpyridine over an oil and aqueous phase, where iron ions are present in the aqueous phase. We investigate two scenarios. In the first and initially anticipated scenario, terpyridine is overwhelmingly molecularly dissolved in the oil phase in the absence of iron ions in the aqueous phase. In the second scenario, we assume that terpyridine forms dimers in oil. These scenarios lead to fundamentally different partitioning behavior and will be compared to experiments. To make the connection to HPE and OMC, it is shown that the chemical equilibrium approach is consistent with the statistical thermodynamic treatment.

Chemical equilibrium. Based on our experimental set-up, we consider demixed oil and water that are in contact via an interface. Initially, terpyridine molecules dissolve in the oil phase. However, upon addition of iron (II) ions in the water phase (beyond a certain concentration of iron ions) in the water phase, terpyridine molecules partition in the aqueous (aq) phase:

$$\mathrm{T_{(oil)}} \rightleftharpoons \mathrm{T_{(aq)}}$$

in which the terpyridine molecule (monomer) is abbreviated as T. Since the concentration of terpyridine in the oil phase is more preferred as a variable due to its low solubility in water, we define the partition coefficient of terpyridine between water and oil (in the

absence of iron or any other metal ions) as:

$$K_p = \frac{[\mathrm{T}_{(aq)}]}{[\mathrm{T}_{(oil)}]} = \exp(-\beta g_H). \tag{12}$$

Here, g_H is the free energy difference between terpyridine in water and terpyridine in oil (more specifically: the difference in standard chemical potential of terpyridine in oil and in water based on 1 M reference concentrations). The subscript H stands for "hydrophobic". Equation 12 is expected to be correct up to the water-solubility of terpyridine which is reported to be approximately 6 mM[S6,S7]. Additionally, bis (terpyridine) iron (II) complexes, $(\mathrm{FeT_2})^{2+}$, are formed in the aqueous phase via the following reaction:

$$2\,\mathrm{T_{(aq)}} + \mathrm{Fe^{2+}} \rightleftharpoons (\mathrm{FeT_2})^{2+}$$

It should be noted that the formation of mono (terpyridine) iron(II) complex, $(\mathrm{FeT})^{2+}$, is neglected based on the equilibrium constants reported previously[S5]. The equilibrium constant for formation of a bis (terpyridine) iron(II) complex in the aqueous phase is given by:

$$K = \frac{[(\mathrm{FeT_2})^{2+}]}{[\mathrm{T}_{(aq)}]^2[\mathrm{Fe^{2+}}]} = \exp(-\beta g_2). \tag{13}$$

Here g_2 is the reversible work of formation of a bis (terpyridine) iron(II) complex, $(\mathrm{FeT_2})^{2+}$, in water. Thus, we write the equilibrium concentration of $(\mathrm{FeT_2})^{2+}$ as

$$[(\mathrm{FeT_2})^{2+}] = K K_p^2 [\mathrm{T}_{(oil)}]^2 [\mathrm{Fe^{2+}}]. \tag{14}$$

Equation 14 shows that in order to form $(\mathrm{FeT_2})^{2+}$ in water, there is an unfavorable step of transferring terpyridine from oil to water ($K_p << 1$), and a favorable step of binding iron

to terpyridine ($K >> 1$). We find the fraction of terpyridine in oil as a function of (free) iron concentration in water through the mass balance $[\mathrm{T}_{(tot)}] = [\mathrm{T}_{(oil)}](1+K_p)+2[(\mathrm{FeT}_2)^{2+}]$ (based on equal volumes of the oil and aqueous phases). Substitution of Equation 14 and solving for $[\mathrm{T}_{(oil)}]$ leads to:

$$f_H = \frac{[\mathrm{T}_{(oil)}]}{[\mathrm{T}_{(tot)}]} = \frac{-1 + \sqrt{1 + 8KK_p^2[\mathrm{T}_{(tot)}][\mathrm{Fe}^{2+}]}}{4KK_p^2[\mathrm{T}_{(tot)}][\mathrm{Fe}^{2+}]}, \tag{15}$$

where we assumed that $K_p << 1$.

Anticipating the experimental results (which point to a significantly steeper dependence of the terpyridine fraction in the oil phase on the free iron (II) ions concentration), we consider the situation that terpyridine is in the form of dimers in the oil phase. In this case the equilibrium follows:

$$\mathrm{T}_{2(\mathrm{oil})} \rightleftharpoons \mathrm{T}_{2(\mathrm{aq})}$$

with the partition coefficient defined as $K'_P = [\mathrm{T}_{2(aq)}]/[\mathrm{T}_{2(oil)}] = \exp(-2\beta g_H) = K_P^2$. The bis (terpyridine) iron (II) complex will then form based on the reaction:

$$\mathrm{T}_{2(\mathrm{aq})} + \mathrm{Fe}^{2+} \rightleftharpoons (\,\mathrm{FeT}_2)^{2+}.$$

Thus, the equilibrium concentration of $(\mathrm{FeT}_2)^{2+}$ is given by

$$[(\mathrm{FeT}_2)^{2+}] = KK_p^2[\mathrm{T}_{2(oil)}][\mathrm{Fe}^{2+}]. \tag{16}$$

Now the fraction of terpyridine in oil, or in its hydrophobic conformation, becomes

$$f_H = \frac{[\mathrm{T}_{(oil)}]}{[\mathrm{T}_{(tot)}]} = \frac{1}{1 + KK_p^2[\mathrm{Fe}^{2+}]}. \tag{17}$$

Here we have used the mass balance $[\mathrm{T}_{2(tot)}] = [\mathrm{T}_{2(oil)}] + [(\mathrm{FeT}_2)^{2+}]$ where $[\mathrm{T}_{2(tot)}] = [\mathrm{T}_{(tot)}]/2$.

It will become clear in the results and discussion section that Equation 17 describes the experimental data much better than Equation 15. Furthermore, Equation 17 is analogous to the Langmuir adsorption equation as can be seen by writing the fraction of occupied sites (θ) of terpyridine dimers (T_2) by iron ions:

$$\theta = \frac{[(\mathrm{FeT}_2)^{2+}]}{[\mathrm{T}_{2(tot)}]} = \frac{KK_p^2[\mathrm{Fe}^{2+}]}{1 + KK_p^2[\mathrm{Fe}^{2+}]} = 1 - f_H. \tag{18}$$

Statistical thermodynamics Here we apply the grand canonical ensemble to obtain the same results as discussed in the chemical equilibrium approach, as it should. The terpyridine molecule (monomer) can be in two states; dissolved in oil (with subscript oil) which is an energetically favorable state for the terpyridine molecule, and an aqueous state (with subscript aq) which is an unfavorable state for the terpyridine molecule (due to its hydrophobicity) in the absence of iron ions. However, the aqueous state can be stabilized due to the higher affinity of the terpyridine molecule in this state to iron ions. It is assumed that terpyridine is in thermodynamic equilibrium between the oil and aqueous phases (states) and that iron ions can be exchanged between terpyridine and the reservoir. Therefore, by using the grand canonical ensemble, the grand partition function of the terpyridine dimers is the summation over all states and occupancy numbers (occupancy

with iron). Therefore, the grand partition function reads

$$\Xi = \sum_{state\ i} \Xi_i = \Xi_{aq} + \Xi_H. \tag{19}$$

Terpyridine in the aqueous state can be either occupied or unoccupied by iron ions. Thus,

$$\begin{aligned}\Xi_{aq} = \exp(-\beta G_H) \sum_{N=0}^{1} \lambda_F^N z(T,N) &= \exp(-2\beta g_H) \sum_{N=0}^{1} \lambda_F^N \exp(-N\beta g_2) \\ &= \exp(-2\beta g_H)(1 + [Fe^{2+}] \exp(-\beta g_2)),\end{aligned} \tag{20}$$

in which $G_H(= 2g_H)$ is the free energy difference between terpyridine dimers in water and in oil phases (states). Here the fugacity of iron (with subscript F) is $\lambda_F = \exp(\beta\mu_F)$ and μ_F is the chemical potential of iron ions. $z(T,N) = \exp(-N\beta g_2)$ is the relevant part of the molecular partition function of terpyridine with N bound iron ions (N = [0,1]). In the second step in Equation 20, λ_F is written as the iron concentration, and at the same time the appropriate standard states for terpyridine and iron ions are applied, consistent with the chemical equilibrium approach. We assume that terpyridine in the oil state does not bind iron and thus $\Xi_H = 1$. From that the fraction of terpyridine in oil, f_H, is given by:

$$f_H = \frac{\Xi_H}{\Xi} = \frac{1}{1 + [Fe^{2+}] \exp(-2\beta g_H) \exp(-\beta g_2)} \tag{21}$$

and the average fraction of terpyridine occupied by iron (in the aqueous state) reads

$$\theta = \langle N \rangle = \frac{\lambda_F}{\Xi} \frac{\partial \Xi}{\partial \lambda_F} = \frac{[Fe^{2+}] \exp(-2\beta g_H) \exp(-\beta g_2)}{1 + [Fe^{2+}] \exp(-2\beta g_H) \exp(-\beta g_2)}. \tag{22}$$

Under the assumption of $\exp(-\beta g_H) = K_p << 1$, it is easily verified that Equations 21 and 22 are equal to Equations 17 and 18, respectively.

1.2.2 Terpyridine-functionalized oligomers (OMC).

In the case of oligomeric terpyridine templates, because of the formation of bis (terpyridine) iron (II) complexes by binding of iron ions onto two terpyridine groups, gel-like networks are expected to form in the aqueous phase where the oligomers are effectively 'bridged' (equivalently crosslinked) by iron ions. Furthermore, it should be noted that we assume that the binding affinity for all terpyridine repeating units (regardless of their local chemical surroundings) are the same. The calculation of the multiplicity $\Omega(N, M)$ in this system is a nontrivial problem. In the unrestricted case, that is, if each terpyridine residue is allowed to bind (via an iron ion) to any other terpyridine residue, that is, residing on the same oligomer or onto any other of the n_p oligomers, there are on the order of $n_p M^2$ possible bonds per oligomer.

However, in a cross linked, gel-like network, the overwhelming majority of these bonds will be geometrically inaccessible. Therefore we approximate $\Omega(N, M) \approx \binom{M}{N}$, just as in the situation for HPE. In this case, the value of M can be expected to be on the order of the number of terpyridine groups per oligomer. Numeric calculations of the oligomer fractions and occupied terpyridine residues via an extreme (and presumably unphysical) scenario $\sum_{N=0}^{M} \binom{nM^2}{N} x^N$, with $x = [Fe^{2+}K]$ as a function of iron ion concentration, reveal significant shifts of the hydrophobic to aqueous transition to lower free iron concentration, but essentially the same value for the cooperativity parameter M compared to the situation with $\Omega(N, M) = \binom{M}{N}$. In the following, we will take the latter approximation for the multiplicity and compare the (effective) values of g_H and g_2 with the situation for terpyridine monomers. Large deviations between these values may point to a significant error in the expression we use for $\Omega(M, N)$. However, the results summarized in Fig.3 in the main paper point to a value of $2g_H + g_2$ for the oligomers that are consistent with independent measurements and reports, as well as with the values obtained for the monomers. The value of $M \approx 16$ is consistent with the existence of on average dimeric clusters of

oligomers, in DCM, where the 16 terpyridine dimers act as binding sites with multiplicity $\Omega(N,M) = \binom{16}{N}$.

Getting the analogues of Eqs. (3, 5), with the definitions of K_p and K from the previous section, the fraction of poly (terpyridine) in the oil phase $f_H = \Xi_H/\Xi$ is given by

$$f_H = \frac{1}{1 + K_p^M(1 + [\mathrm{Fe}^{2+}]K)^M}, \tag{23}$$

and for the total fraction of the occupied binding sites we find

$$\theta = \frac{\langle N \rangle}{M} = \frac{K[\mathrm{Fe}^{2+}]}{1 + K[\mathrm{Fe}^{2+}]}(1 - f_H). \tag{24}$$

So, as long as $[\mathrm{Fe}^{2+}] >> K^{-1}$, (with concentrations in M) we have $\theta = 1 - f_H$, even at very low iron concentrations.

1.3 Composition dispersity in HPE copolymers

Within HPE we may identify two distinct classes. Homopolymers, composed of a single repeat unit, where the ionizable and hydrophobic group are on the same unit, and copolymers, where there are multiple types of repeating units with potentially different ionization and hydrophobic penalty energies. If the copolymerization of the units is statistical or random there will be a dispersity in the ratio of the repeating units on each chain over the whole polymer sample. In the following we will examine a (compositionally monodisperse) homopolymer, poly(6-(acryloyl) aminohexanoic acid) (PAHA), and compare its pH-dependent partitioning to that of a (compositionally polydisperse) random copolymer, poly(n-butyl acrylate-*stat*-acrylic acid) (PBA-AA). The ability to predict the effect of this dispersity on the transitions of HPE is an important test case of our theoretical framework, which includes specific polymer composition information such as the

number of repeating groups and the total hydrophobicity of a chain.

Within copolymers, we may identify two groups of relevance for our analysis: those whose repeating units always contain ionizable groups and those which contain some monomers without ionizable groups. This is an important distinction as the broadening in the latter group is expected to be much more severe and it is our focus here. This is the case for the PBA-AA used in our experiments and whose pH-dependent partitioning is shown in Fig.4.

The origin of the expected broadening lies within the large difference in both ionization and hydrophobic energy between the two monomers. An acrylic acid group is weakly acidic and weakly hydrophobic in its protonated form, while the butyl acrylate group is not ionizable and fairly hydrophobic. The varying ratio of each of the groups within the compositionally disperse chains, leads to a large difference in the balance of ionization and hydrophobic energy. Subsequently the transition-pH of each of the chains will differ, leading to an overall spread-out transition.

To apply our theoretical framework to this class of HPE it is necessary to define the total number of repeat units on the polymer, M_t. For a binary copolymer we then have $M_t = N_1' + N_2'$, where N_i' is the number of units for the ith monomer, and if we denote the monomer type 1 as the one with ionizable groups then $M = N_1'$.

We will also include length dispersity in this model and therefore we have two distributions for the make-up of the polymer, a length and composition distribution. The inclusion of a small amount of length dispersity, which will always be present in conventionally synthesized polymers, increases the smoothness of the curves and improves the match with the experimental data. We illustrate this effect in Fig. S2(a).

The usual experimental measure of the length dispersity of a polymer extracted from, for example, size exclusion chromatography (SEC), is a length dispersity value, Đ. It is possible to relate Đ to the mean and variance of a distribution through the following

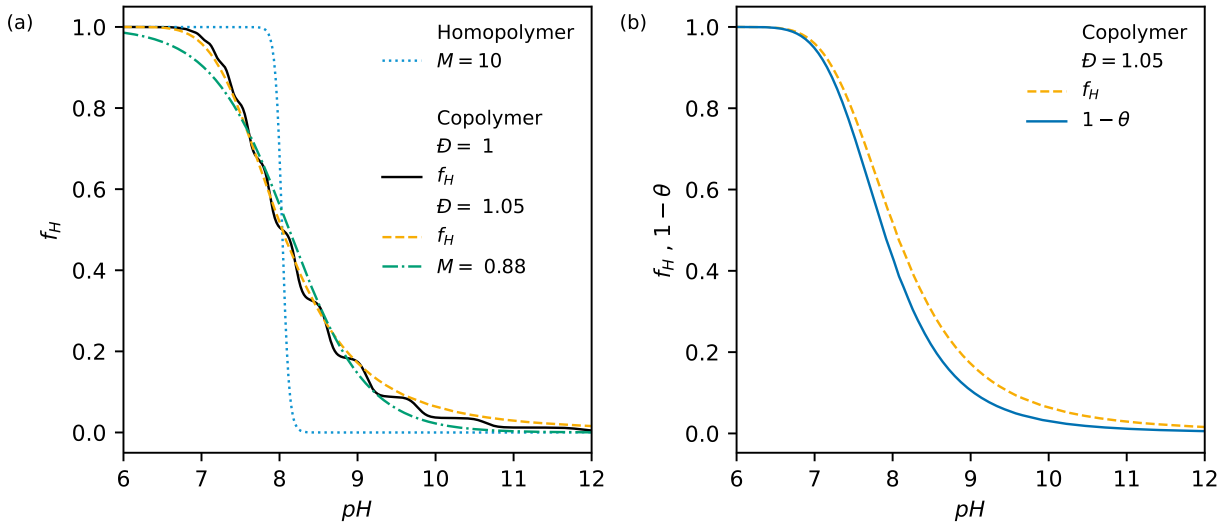

Figure S2: **Compositional dispersity leads to a spread in the ionization transitions of hydrophobic polyelectrolytes. (a)** Calculated plots (Eq. (28)) of the fraction of HPE in the hydrophobic state for a homopolymeric (blue dotted line) and compositionally disperse polymers with (dashed yellow line) and without length dispersity (solid black line). Equation (9) was used in Eq. (28) for the f'_H curve of an individual chain. The hydrophobic penalty is set as $\beta G = g_H M = g_{H,1}N'_1 + g_{H,2}N'_2$. For the above curves the global variables used are: $\langle M_t \rangle = 20$, $g_1 = g_2 = 4.0$, $\langle M \rangle = 10$. The dashed green line is a fit of the calculated curve for the length and compositionally disperse system with the general Eq. (9). **(b)** f_H (Eq. (28)) and $1 - \theta$ (Eq. (29)) curves for a compositionally and length disperse copolymer. Note the lack of full overlap between the curves. The same global variables are used as in (a).

expression[S8]:

$$Đ = 1 + \frac{\sigma^2}{\mu^2}. \tag{25}$$

Where the mean, μ, and variance, σ^2, are sample variables in this case.

Throughout this work we will be considering short ($<$ 100 repeat units) polymers and therefore we have opted for a log-normal distribution of the polymer lengths, when treating the length dispersity in these systems. This distribution has been shown to be suitable for polymers synthesized using controlled radical polymerization techniques[S9]. The weight of each chain length, M_t, can be expressed as:

$$\omega(M_t) = \frac{1}{M_t\sigma'\sqrt{2\pi}} \exp\left(-\frac{1}{2}\left(\frac{\ln M_t - \mu'}{\sigma'}\right)^2\right). \tag{26}$$

The variables μ' and σ' are related to the actual expectation value and variance of M through the following equations: $\mu = \exp(\mu' + \frac{\sigma'^2}{2})$ and $\sigma^2 = (\exp(\sigma'^2) - 1)\exp(2\mu' + \sigma^2)$. $\omega(M)$ then becomes a function of the mean, μ, and variance , σ^2, of this log-normal distribution and consequently of the Đ of the polymer.

There are a variety of different ways to take into account the compositional dispersity of copolymers. For our analysis here we will take the simplest approach which is a binomial distribution, leading to an expression for the weight as follows:

$$\omega'(M_t, N_1', p) = \binom{M_t}{N_1'} p^{N_1'}(1-p)^{M_t - N_1'}. \tag{27}$$

Where p is the probability of adding a monomer of type 1 to the end of a chain. A binomial distribution does not take into account the fact that the reactivity for a particular monomer, with respect to the chain end, usually depends on the last monomer which was attached to the chain. This can be taken into account by using a terminal model (or even a penultimate model[S10]), which leads to the well known reactivity ratios[S11]. Knowing these ratios, it would be possible to find the weight $\omega'(M_t, N_1')$ for a particular copolymer. The polymers we deal with in this work, however, present mostly very similar chemical reactivities with respect to either of the monomers being at the chain end and therefore a binomial distribution should suffice. The probability, p, is therefore simply a ratio of the concentration of each of the monomers in the initial reaction vessel.

The expression for the fraction of chains in the hydrophobic (or aqueous) state follows from Eq. (9) where we then apply weights for each of the chain lengths and compositions:

$$f'_H = \sum_{M_t=1}^{\infty} \omega(M_t) \sum_{N'_1=0}^{M_t} \omega'(M_t, N'_1) f_H(M_t, N'_1). \tag{28}$$

The hydrophobic penalty is set as a linear combination of each of the groups ($\beta G = g_{H,1}N'_1 + g_{H,2}N'_2$) in Eq. (9). As before the fraction of polymer chains in the aqueous state is $f'_{aq} = 1 - f'_H$.

As in the previous section, an expression for the total fraction of ionized sites for the polymer sample can be found using the following equation:

$$\theta = \frac{1}{\langle M \rangle} \sum_{M_t=1}^{\infty} \omega(M_t) \sum_{N'_1=0}^{M_t} N_1 \omega'(M_t, N'_1) \theta'(M_t, N'_1) \tag{29}$$

Where $\langle M \rangle$ is the mean number of ionizable groups per chain over the whole polymer sample ($\langle M \rangle = \sum_{M_t=1}^{\infty} \omega(M_t) \sum_{N'_1=0}^{M_t} N'_1 \omega'(M_t, N'_1)$).

Figure S2(a) shows an example of the broadening expected for a length and compositionally disperse binary copolymer, where in this case only one type of copolymer has ionizable groups. Equation (9) was used in Eq. (28) for the f_H curve of an individual chain. The extensive broadening is clearly apparent compared to the homopolymer situation. It is worth noting that in this particular figure the hydrophobic penalty per group is identical for both monomers and it would be expected that the broadening is even more significant if we allowed the values for the penalties to differ, which will usually be likely due to the difference in the chemical nature of these groups.

Finally, an interesting feature of these transitions is the asymmetry around the $f_H = 0.5$ point. This occurs due to the simultaneous change in transition-pH and sharpness for the different chains in these disperse polymers. Chains with a higher M/M_t fraction will transition at lower pH values and will present sharper transitions. Conversely, chains with a lower M/M_t ratio will transition later and less sharply. This leads to the "tail" seen for

the latter half of the transition as the pH increases.

As might be expected from a polymer sample where the number of ionizable groups varies drastically between polymers with similar lengths, the polymers described in this section present deviations between the ionization fraction and hydrophobic fraction curves. Figure S2(b), plots the two curves for an example polymer sample. The ionization fraction curves has an earlier transition-pH, again due to chains with a higher proportion of ionizable groups transitioning earlier and these chains having a larger weight in the ionization fraction calculation.

1.4 Fractionation calculation

The numerical procedure to calculate the predicted result of an experimental fractionation of a chemically disperse copolymer HPE follows from the equations shown in the previous section. They detail the set of weights of each of the different chains in a chemically disperse polymer, which in the case of the calculation shown in Fig. 4 (main text), is a polymer with a disperse ionizable group number to total length ratio.

We start from the set of weights $\omega(M_t, M)$ for the initial disperse polymer, where M and M_t are the number of ionizable groups and the total number of groups on the chain, respectively. This information could be found from an understanding of the polymerization mechanics (e.g. random polymerization) and a measurement of the length dispersity using size exclusion chromatography. In the case of the calculation for PBA-AA in the main text, M corresponds to the number of acrylic acid (AA) groups on a chain. For this system, where we consider the polymerization of the precursor to PBA-AA, PnBA-tBA, between n-butyl acrylate (nBA) and t-butyl acrylate as a random process, $\omega(M, M_t)$ is the combination of Eq. (27) and Eq. (26).

To carry out the numerical fraction procedure we create a set a set of weights for a wide range of potential (M_t, M) values. We then calculate the fraction of the chains in

the aqueous phase (f_{aq}) for each (M_t, M) couple at the first fractionation pH in Fig. 4. A general equation for this is as follows:

$$f_{aq}(M_t, M, pH_1) = \frac{\exp(-\beta G(M_t, M)))(1 + 10^{pH_1 - pK_a})^M}{1 + \exp(-\beta G(M, M_t)))(1 + 10^{pH_1 - pK_a})^M} \tag{30}$$

The expression for the conformational penalty is $\beta G = g_{H,1} N_1' + g_{H,2} N_2'$, where N_1' is the number of hydrophobic groups defined as $N_1' = M_t - M$. The values of the hydrophobic penalties per group, $g_{H,1}$ and $g_{H,2}$, can be found, for a particular polymer sample, from a fit of the initial partitioning transition of the polymer in the two-phase oil and water system. See Fig. 4(a) for fits of the PBA-AA transition the fractionation calculation is based on.

The product of $f_{aq}(M_t, M, pH_1)$ and $\omega(M_t, M)$ now gives the weight of each chain configuration in the aqueous phase. We denote this set of weights $\omega'(M_t, M)$. In the fractionation scheme in Fig. 4(c), this aqueous phase is then exposed to a new, empty oil phase and its pH is modified. We now carry out an analogous calculation to calculate the weights of the chains that partition into the oil phase. We use the expression for the fraction of chains in the hydrophobic state this time, given by:

$$f_H(M_t, M, pH_2) = \frac{1}{1 + \exp(-\beta G_H(M_t, M))(1 + 10^{pH_2 - pK_a})^M} \tag{31}$$

The product of $\omega'(M, M_t)$ and $f_H(M_t, M, pH_2)$ now gives the final set of weights in the oil phase $\omega''(M_t, M)$. The full operation to find the set of weights for the fractionated polymer is therefore:

$$\omega''(M_t, M) = \sum_{M_t=1}^{\infty} \sum_{M=0}^{M_t} \omega(M_t, M) f_{aq}(M_t, M, pH_1) f_H(M_t, M, pH_2) \tag{32}$$

The polymer sample at this step in the fractionation procedure is now extracted from the two-phase system and isolated, affording us a sample with a distribution of chains

described by $\omega''(M_t, M)$.

The calculation for the chain efficiency (fraction of chains in the final sample with respect to the initial sample) can be found from the sum of all the values in the final weight matrix before normalization is carried out.

If this fractionated sample is now introduced into a separate two-phase system, as shown in Fig. 4(c), its partitioning behavior can be described by introducing this matrix of weights $\omega''(M_t, M)$ (after normalization) into Eq. (28). This is identical to calculating f_H from Eq. (31) for every (M_t, M) couple and doing a summation of the product of this value and $\omega''(M_t, M)$, for every pH value.

2 Hydrophobic polyelectrolytes (HPE)

2.1 Monoprotic acids

2.2 Materials and instrumentation

2.2.1 Materials

1-pentanol ($\geq$ 99%, ACS reagent), *N,N*-dimethylformamide (DMF, $\geq$ 99.8%, ACS reagent), potassium chloride (KCl, Analysis grade), potassium carbonate (K_2CO_3, anhydrous, 99%, ACS reagent), sodium azide (NaN_3, 99.5%), boric acid (99.5%, ACS reagent), phosphoric acid (99%), copper(II) bromide ($CuBr_2$, 99%), citric acid (99.5%), *n*-butyl acrylate (nBuA, stabilized, synthesis grade), silica gel (Davisil grade 633, 60Å pore size, 200-425 mesh particle size), ethyl α-bromoisobutyrate (EBIB, 98%), propargyl bromide (80 wt.% in toluene, stabilized), *N,N,N',N",N"*-pentamethyldiethylenetriamine (PMDETA, 99%), tris[2-(dimethylamino)ethyl]amine (Me_6TREN, 98%), acryloyl chloride (97%, stabilized) and 2,2'-azobis(2-methylpropionitrile) (98%) were purchased from Sigma-Aldrich.

Potassium hydroxide (KOH, Analysis grade), trisodium citrate dihydrate (analysis

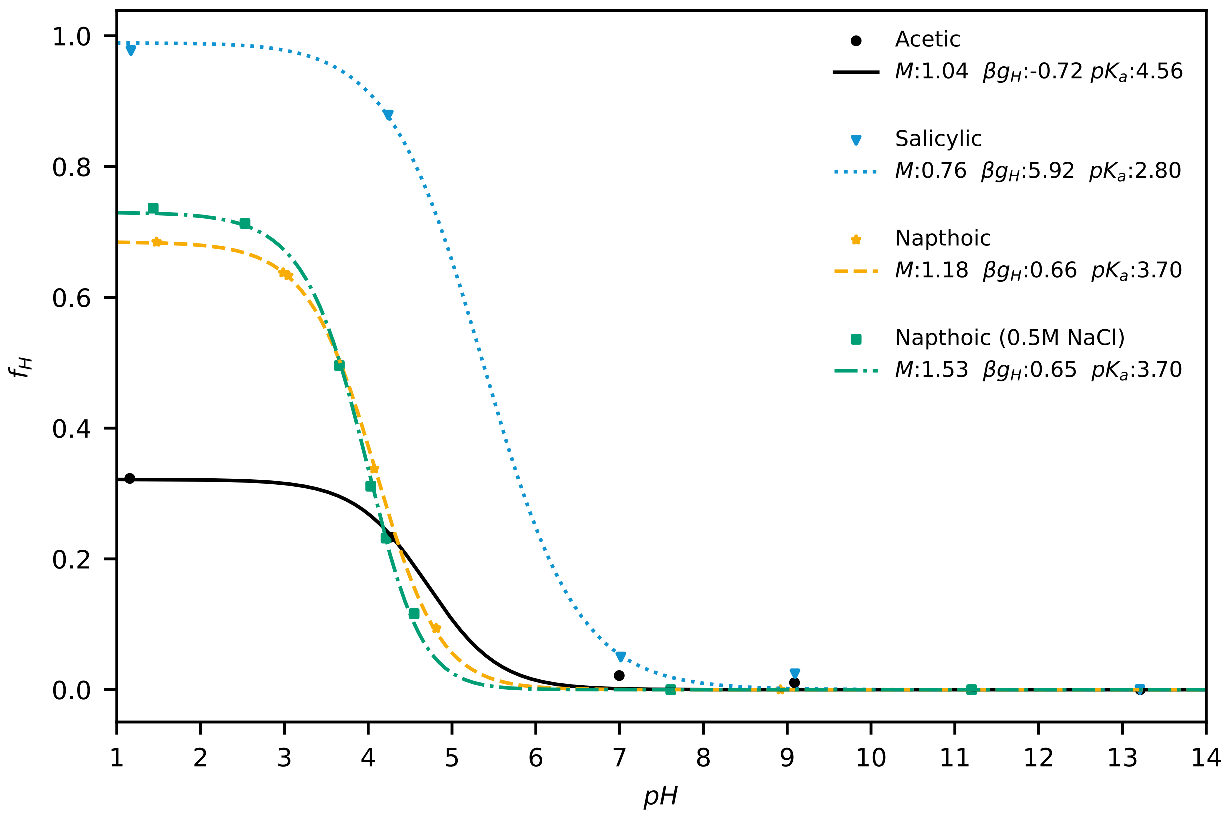

Figure S3: **Monoprotic acids show broad partitioning behavior in oil-water systems.** Fraction of monoprotic acids in the oil-phase (f_H) of two-phase oil and water systems. Acetic and salicylic acid octanol-water partitioning data is taken from reference[S12]. The partitioning data at the highest pH value was scaled to reach 0 for these acids. Napthoic acid isooctane-water partitioning data is taken from reference[S13] for two different salt concentrations. The data series have been fitted (plotted curves) with Eq. (9) and using pK_a values from[S12] and[S14] for the two sets of data, respectively. Fit parameters are shown in the legend.

grade, ACS reagent), ethanol (absolute for analysis, ACS reagent), acetic acid (glacial 100%, ACS reagent), potassium iodide (KI, analysis grade), trifluoroethanol (TFE, 99%) and sodium hydroxide (NaOH, Analysis grade) were purchased from Merck.

Copper wire (0.25mm diameter, 99.98%), *t*-butyl acrylate(99%,stabilized), copper(I) bromide (CuBr,98.1%) and dichloromethane (DCM, anhydrous, 99.7%,stabilized) were purchased from Alfa Aesar.

7-hydroxy-4-methylcoumarin (4MU, 97%), basic alumina (Brockmann 1, 60Åpore size,

40-300 mesh particle size) and fuming hydrochloric acid (37% solution in water) were purchased from Acros Organics.

Methanol (HPLC grade), n-hexane (HPLC grade), chloroform (HPLC grade, stabilized), diethyl ether (HPLC grade, stabilized) and ethyl acetate (analytical reagent grade) were purchased from Biosolve B.V.

6-aminohexanoic acid (99%), magnesium sulfate ($MgSO_4$,anhydrous, $\geq$ 99.5%) and tetrahydrofuran (THF, anhydrous, 99.9%, stabilized) were purchased from Thermo Fischer Scientific.

Acetone ($\geq$ 99.5%) was purchased from VWR chemicals.

Trifluoracetic acid (TFA, 99%) was purchased from Honeywell.

Deuterated dimethylsulfoxide-d6 (DMSO-d6, 99.8 atom%D) and deuterated chloroform-d1 ($CDCl_3$, 99.8 atom%D, stabilized) were purchased from Carl Roth.

4-cyano-4-[(ethylsulfanylthiocarbonyl)sulfanyl]pentanoic acid (CECTP, $\geq$ 98%) was purchased from Polymer Source inc.

Milli-Q water (deionized, 18.2MΩcm, 21 °C) was used throughout synthetic procedures when referring to (deionized) water.

2.2.2 Instrumentation

UV-Vis absorbance spectroscopy was carried out on the CLARIOstar microplate reader (BMG LABTECH) using UV-STAR 96-well plates (Greiner).

pH measurements were carried out on a SevenExcellence *pH*-meter (METTLER TOLEDO) using an InLab Micro probe (METTLER TOLEDO). Standard calibration solutions (Hanna Instruments), *pH* 4.01 and 10.01, were used to calibrate the instrument before the measurements runs. Measurements of the solutions after the experimental run were used to correct for instrumental drift.

During the process of polymer precipitations an Allegra X-12R (Beckman Coulter)

centrifuge was used at 3273g in 50 mL plastic centrifuge tubes. When centrifuging two-phase pentanol and water solutions 10 mL glass centrifuge tubes were centrifuged at 931 g.

NMR measurements were carried out on a 400 MHz spectrometer (Agilent) and integrated using MestReNova (Mestelab Research) software.

SEC measurements of the polymer samples were carried out on an Alliance HPLC e2695 (Waters) connected to a Waters 2414 refractive index detector and a Waters 2489 UV-Vis detector. A 300 mM ammonium acetate buffer (pH 9) was made in Milli-Q water and then filtered using a Whatman RC55 0.45 μm pore filter. Samples were dissolved at a concentration of 1 mg/ml in the eluent and then filtered through a Phenex RC 0.2 μm syringe filter. The separation column employed was an Agilent PL-aquagel 30 and a flow rate of 1 ml/min was used. PEG standards were chosen for the calibration of the instrument.

2.3 Experimental procedure

Two types of experiments were performed to analyze the partitioning transitions of HPE:

The first uses a buffered aqueous solution in contact with the oil phase. This allows for a series of samples at different pH values to be easily made. The effect of possible pH drift over time is reduced and long-term observation of the samples is possible. From this experiment it is possible to extract the fraction of the polymer chains in either of the phases but ionization data is inaccessible. The second experiment gives access to the ionization fraction of the polymer. This requires unbuffered solutions which are carefully titrated to measure the pH response of the aqueous phase of the two-phase system. It is also possible to extract the fraction of the chains in either of the phases using this method.

The choice of method will depend on the desired application. An assay style screenings of a series of polymers may benefit from the simplicity and speed of the buffered method

even if ionization data is not measurable. Before describing the particular setup used to yield the experimental results described in this study it is worth stating some general design principles for the phases used and for the polymer being investigated.

The two liquids chosen must be at least partially immiscible as to allow for two phases to form. One must be aqueous, as its pH will be varied and the other must be hydrophobic enough for there to be an important offset in the hydrophobic penalty for the polymer between both phases and for ionization of the acid or basic groups to be suppressed. In the experiments presented here pentanol was chosen as the hydrophobic organic phase.

The HPE chosen must, by definition, contain weakly ionizable groups which afford it its pH sensitivity. It must contain a certain amount of hydrophobic groups for the hydrophobic penalty to be large enough to shift the transition-pH a few pH units away from the pK_a of the ionizable groups. The HPE investigated in this work are shown in Table S1. Being able to attain well-defined polymers, with predictable composition, length and dispersity, is of great importance in our investigation of the properties of hydrophobic polyelectrolytes. We have therefore employed "controlled" radical polymerization techniques to synthesize the polymers used in this study. See, for example[S15–S17], for an overview of the RAFT and ATRP techniques used and their underlying mechanisms.

Finally, a consideration for the measurement methods must be made. UV-Visible (UV-Vis) spectrometry is the method of choice used in this study to measure the fraction of the polymer in the oil phase. The use of a plate reader spectrometer, allowing for the parallel measurement of dozens of samples, simplifies the measurement of large series of samples and therefore this was the method chosen in this study. This requires there to be a chemical moiety on the polymer that has a high absorbance in this spectral range and for the liquid in the phase being measured not to absorb in a similar range. It is worth noting that many dyes are pH sensitive and therefore measurement in the aqueous phase might be unsuitable. It is also possible to use methods that do not rely on the absorption of light.

Table S1: Chemical structures of the hydrophobic polyelectrolytes used in this study. Hydrophobic groups are depicted in red and ionizable groups are depicted in blue.

Name	Chemical structure	Degree of polymerization (DP)
poly(6-(acryloyl) aminohexanoic acid) (PAHA)	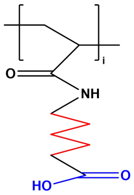	$18 - 65$
poly(butyl acrylate - acrylic acid)) (PBA-AA)	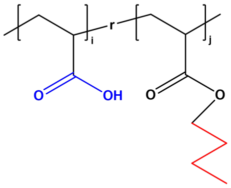	20

This is the case for refractometry which is a commonly used method to quantitatively measure concentrations of dissolved molecules.

2.3.1 Buffered system

Due to the wide range of pH values (pH $3 - 12$) of interest a series of pentanol-saturated Britton-Robinson buffers (equimolar acetic, phosphoric and boric acid, 40 mM in Milli-Q water) with an added 0.7 M KCl were made and titrated using 0.7 M KOH to the desired pH values.. 0.1 w/v% sodium azide (NaN3) was added to the buffer series to avoid any bacterial growth followed by an excess amount of pentanol as to saturate them.

The HPE being analyzed was then dissolved in water-saturated pentanol. The concentration of these solutions is usually around 1 mg/mL. The exact concentration chosen for this stock solution will depend on the absorption value of the specific wavelength we have chosen to analyze. A value of 2.0 was aimed for.

Equal volumes ($1.5 - 2$ mL) of a particular buffer solution and of the pentanol-polymer

solution were added together, and they were then thoroughly shaken and/or left on a roller. The solutions were allowed to equilibrate over a couple of days and were periodically shaken throughout or left on a roller.

Solutions made with the chemically disperse polymer, poly(butyl acrylate - acrylic acid) (PBA-AA), presented persistent emulsions. Therefore when using this polymer, these solutions were centrifuged at 931 g for 30 min.

For all polymers the two phases were then separated by pipetting off the top pentanol phase. The fraction of polymer in the oil phase was then measured using UV-Vis spectrometry and the pH of the aqueous phases was measured. Standard calibration solutions (Hannah instruments, pH 4.01 and 10.01) measured at the end of the measurement run were used to calibrate the pH-meter [1].

2.3.2 Unbuffered system (titration)

A 0.7 M KOH solution and a 0.7 M KCl solution were made with degassed Milli-Q water. The 0.7 M KCl solution was then acidified using a degassed 1 M HCl solution to a pH of around 3. A stock water-saturated pentanol-polymer solution with a concentration of around 1 mg/mL was made and then degassed.

Under a flow of N_2 gas, 2 mL each of the pentanol-polymer and 0.7 M KCl acidified solution were added to a series of glass centrifuge tubes. A known amount of the 0.7 M KOH solution was then added to each sample in the series. The amount of each solution added was tracked by measuring the added weight at each step.

The two-phase samples were then thoroughly shaken over the course of a few hours and allowed to equilibrate overnight. They are then centrifuged at 931 g for 30 min.

[1] A comment can be made at this point on the interpretation of the pH measurements of a pentanol-saturated solution (~2.2 %w/w). Following from [S18,S19] we might expect a (small) offset of the measurement with respect to a fully aqueous phase, therefore the magnitude (ΔpH) of the transition region is expected to be unaffected. The same pH-meter was used for all experiments so we can consider all experiments directly comparable.

To avoid possible CO_2 dissolution into the aqueous phase, the pH of the aqueous solutions was measured before separation of the oil and aqueous phases. The pH-meter was inserted through the oil phase into the aqueous phase.

The solutions were then separated and the fraction of polymer in the oil phase is measured using UV-Vis spectrometry.

2.4 Polymer synthesis and characterization

Being able to attain well-defined polymers, with predictable composition, length and dispersity, is of great importance in our investigation of the properties of hydrophobic polyelectrolytes. We have therefore employed "controlled" radical polymerization techniques to synthesize the polymers used in this study. See, for example[S15–S17], for an overview of the RAFT and ATRP techniques used and the underlying mechanisms. Each polymer's synthesis will now be described in turn including the synthesis of any monomers or dyes used to synthesize these polymers.

2.4.1 poly(6-(acryloyl)aminohexanoic acid) (PAHA)

The synthesis of the homopolymeric poly(6-(acryloyl)aminohexanoic acid) (PAHA) was carried out using RAFT polymerization.

2.4.2 Synthesis of 6-(acryloyl)aminohexanoic acid

The monomer polymerized into the PAHA polymer was synthesized following the description of Hetzer et al.[S20].

To a 100 mL round bottom flask NaOH (4.5 g, 0.1 mol, 2.5 eq.) and 6-aminohexanoic acid (5 g, 0.04 mol, 1 eq.) were added and then dissolved using 30 ml of deionized water. After cooling down the mixture in an ice bath, acryloyl chloride (3.8 ml, 0.05 mol, 1.25 eq.) was added dropwise at a rate of 0.12 mL/min (using a syringe pump and a teflon tube).

The solution was stirred while in the ice bath for 2 h. After removing the solution from the ice bath, it was acidified using an HCl solution (1 : 1 fuming to deionized water) until *pH* 1 was reached. A white precipitate formed which subsequently dissolved when 40 mL of ethyl acetate was added to the flask. The mixture was then transferred to a separatory funnel and after removal of the ethyl acetate phase, the water phase was washed three times with 30 mL of ethyl acetate. The combined organic phases were then washed with 100 mL of acidified (pH 1) water and dried over excess $MgSO_4$. After filtration of the mixture it was concentrated *in vacuo* to return a white powder. To further purify the product, it was dissolved in a minimum amount of ethanol ($\sim$ 2 mL) and precipitate into 40 mL of cold diethyl ether. This was repeated three times, after which the precipitate was dried under a stream of dry N_2. Average yields for this procedure were approximately 30%.

^{1}H NMR (400 MHz, DMSO-d6), shown in Fig. S4(a), was carried out to confirm the presence of the desired compound.

2.4.3 RAFT polymerization of 6-(acryloyl)aminohexanoic acid

To yield the PAHA polymer, with good control over its length dispersity, RAFT polymerization was employed. The specific procedure employed is based on the polymerization described in Brodszkij et al.[S21].

Three different degrees of polymerization (DP) were targeted, which are achieved by varying the ratio of reagents during the polymerization. Note that only the shortest polymer was used in the partitioning experiments. Data for all three is shown to illustrate the ability to target different DPs. The degree of polymerization of a RAFT reaction can be approximated to the ratio of the molar concentration of the monomer with respect to the CTA, if we assume full conversion[S15]. Table S2 summarizes the different amounts of reactants used to yield the polymers. The following general procedure was used to carry

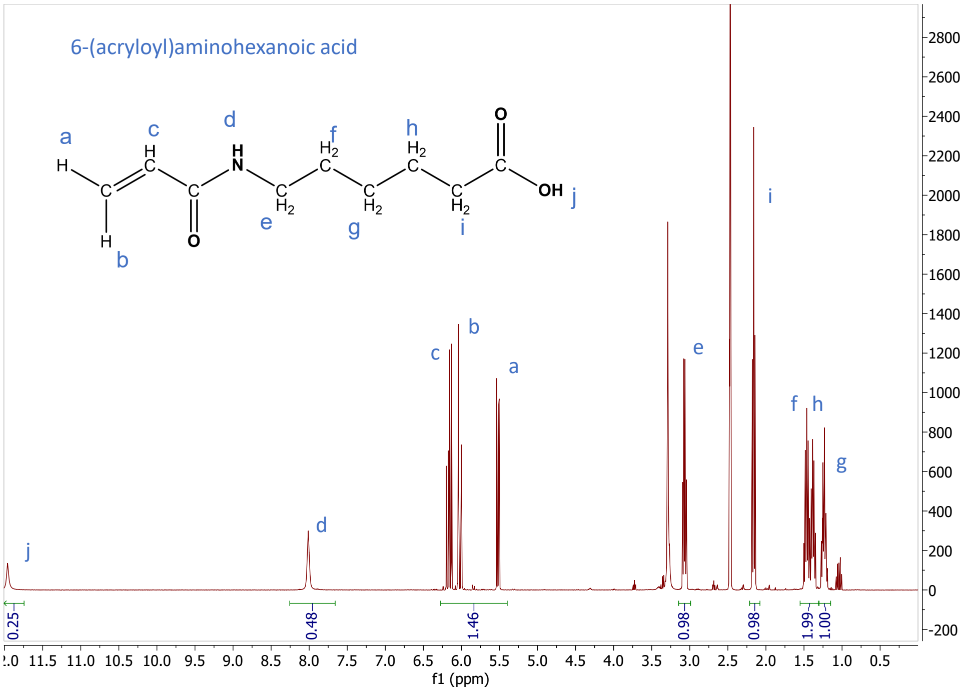

(a) ^{1}H NMR (400 MHz, DMSO-d6) of the final product of the 6-(acryloyl)aminohexanoic acid synthesis

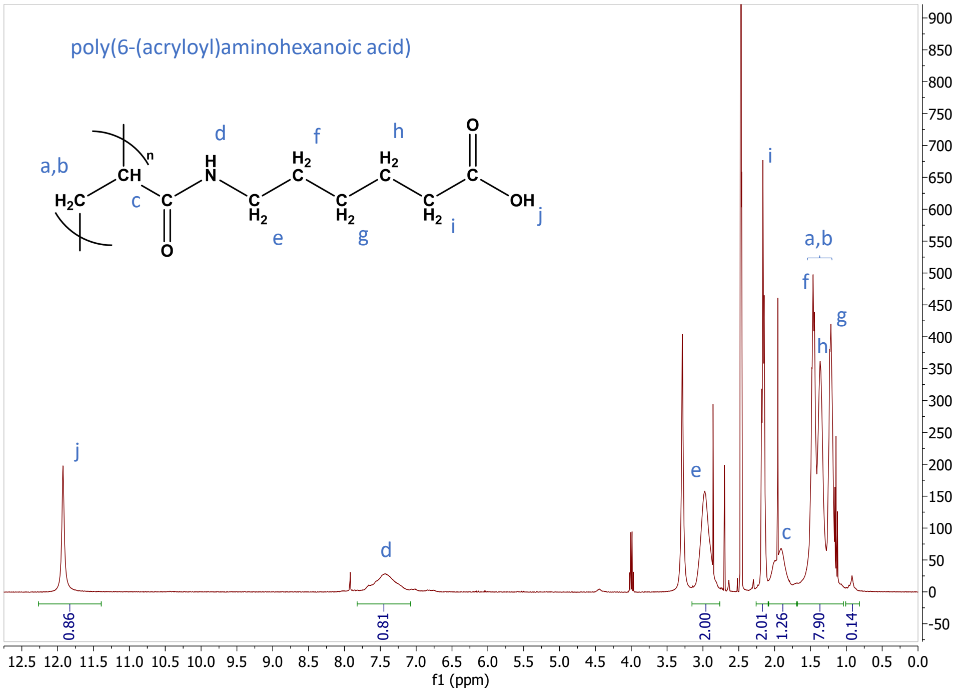

(b) ^{1}H NMR (400 MHz, DMSO-d6) of the final product of a DP=20 PAHA polymer synthesis.

Figure S4: ^{1}**H-NMR spectra** of the PAHA synthesis.

out the polymerization.

Table S2: Amounts of reactants used to yield different degrees of polymerization (DP) of PAHA using RAFT polymerization. Equivalents are normalized with respect to the CTA for each reaction run.

Target DP	monomer (mg/eq.)	AIBN (mg/eq.)	CTA (mg/eq.)	DMF (mL)
20	255.3/20	2.3/0.2	19.7/1	1.8
40	243.6/40	1.1/0.2	8.8/1	1.8
80	251.1/80	0.6/0.2	5.4/1	1.8

In a 4 mL vial the chosen amounts of 6-(acryloyl)aminohexanoic acid monomer, AIBN and the CTA, CECTP, were dissolved in DMF. The solution was then degassed by bubbling dry N_2 through it for 1 h. The vial was placed in an oil bath set at 75 °C and left stirring overnight. The whole reaction mixture is then pipetted into 40 mL of cold diethyl ether. The precipitate formed is reprecipitated a further two times by dissolving it in a minimum amount of ethanol ($\sim$ 3 mL) and pipetting the solution into cold ethyl acetate. The bulk of the solvent is removed under a stream of dry N_2 and then the polymer is dried *in vacuo* overnight. Average yields for this procedure were approximately 60%.

2.4.4 PAHA polymer characterization

^{1}H nuclear magnetic resonance (NMR) (400 MHz, DMSO-d6), shown in Fig. S4(b), was carried out to confirm the presence of the desired compound. Note the broadening of the peaks with respect to the monomer and the disappearance of any vinyl peaks between 5.5 and 6.5 ppm. The NMR spectrum for all three samples with a different target degree of polymerization were very similar.

Further, size exclusion chromatography (SEC) was used to analyze the hydrodynamic volume distribution, as a proxy for the length distribution, of the synthesized polymers. The SEC was run using an ammonium acetate buffer (300 mM, *pH* 9) to ensure the full solubilization of the polymer chains in the solvent. Both refractive index (RI) and UV-VIS

absorption measurements were employed to detect the polymer eluted from the column. Table S3 summarizes the results of the SEC analysis of the target DP=20, 40 and 80 polymers.

Table S3: Summary of SEC analysis (derived from PEG standards) of PAHA polymers with different target degrees of polymerization. Only data derived from RI data is shown here.

Target DP	Mw (kDa)	Mn (Da)	Đ
20	5.8	5.5	1.06
40	9.0	7.8	1.15
80	13.4	10.2	1.31

As expected the molecular weights of the three different polymers do increase with an increase in the targeted DP. However, due to the large difference in the chemical structure of the PAHA polymer and the PEG standard used, the molecular weights derived from SEC will not be accurate. The length dispersity values (Đ) on the other hand, remain a good estimate of the molecular weight distribution of the sample. There is a marked increase in the dispersity (Đ) of the polymer as we increase the target DP which coincides with a tail appearing in the RI chromatograms for the polymers shown in Fig. S5.

The RI traces indicate the general mass concentration over time of the polymer eluting from the column, conversely the UV (308 nm) traces, shown in Fig. S6, show predominantly the elution of the UV active moiety on the polymer. In this case, the UV active group will be the trithiocarbonyl end group, which originates from the CTA used in the polymerization. It is interesting to note that the shapes of the RI and UV traces do not match well. The UV traces do not present tails in the distribution, but actual bimodal character. This indicates a correlation between the trithiocarbonyl group retention at the end of a polymer and the chain length.

Although, in principle, we can derive quantitative degrees of polymerization from both the NMR and SEC analysis of the final products, the data presented here does not allow

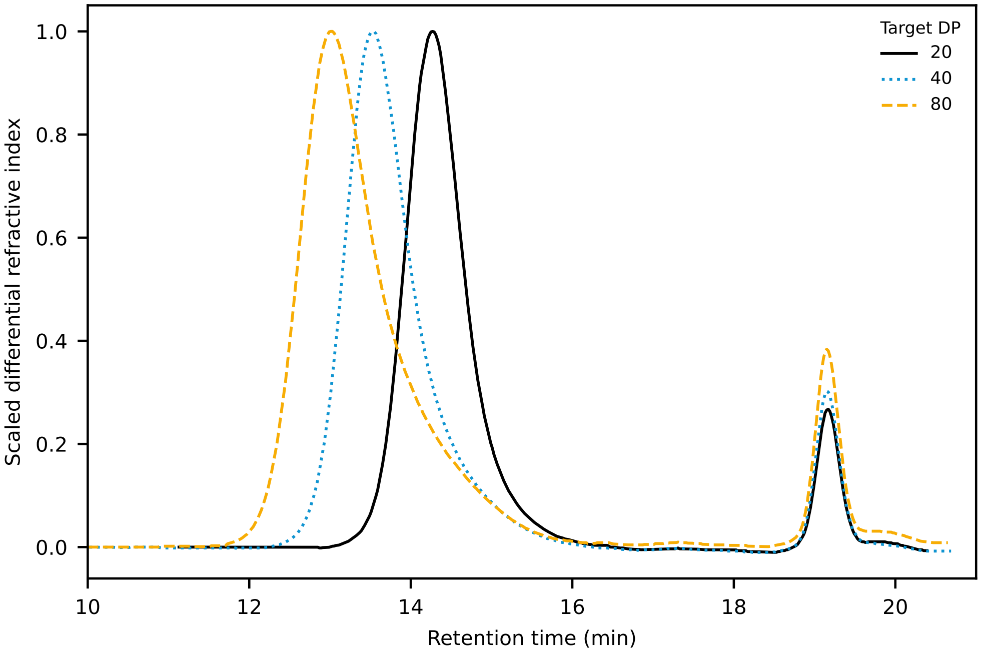

Figure S5: **RI SEC chromatograms** of the different PAHA polymers.

for this. The NMR spectra does not have an easily identifiable reference peak from either the initiator or the CTA, and the use of a very structurally distinct reference for the SEC, namely PEG, only allows for qualitative length comparison. To ascertain an, at least, rough measure of the average length of the polymers we can use the monomer conversion calculated from the NMR measurements of the reaction mixture after it is precipitated in cold diethyl ether (Table S4).

An estimate for the concentration of total chains that are initiated in the system is given by the initial molar concentration of the CTA, which can then be compared to the estimated number of equivalents of monomer which have reacted.

Monomer conversion data was not available for the target DP=20 sample. Instead we can estimate a value for the degree of polymerization of this polymer using the SEC data of the other samples. This analysis includes the target DP=20^e, which was synthesized with the same conditions are the other target DP=20 sample but with the addition of 1 equivalent of eosin-Y acrylate (polymer not used in this work). We created a calibration

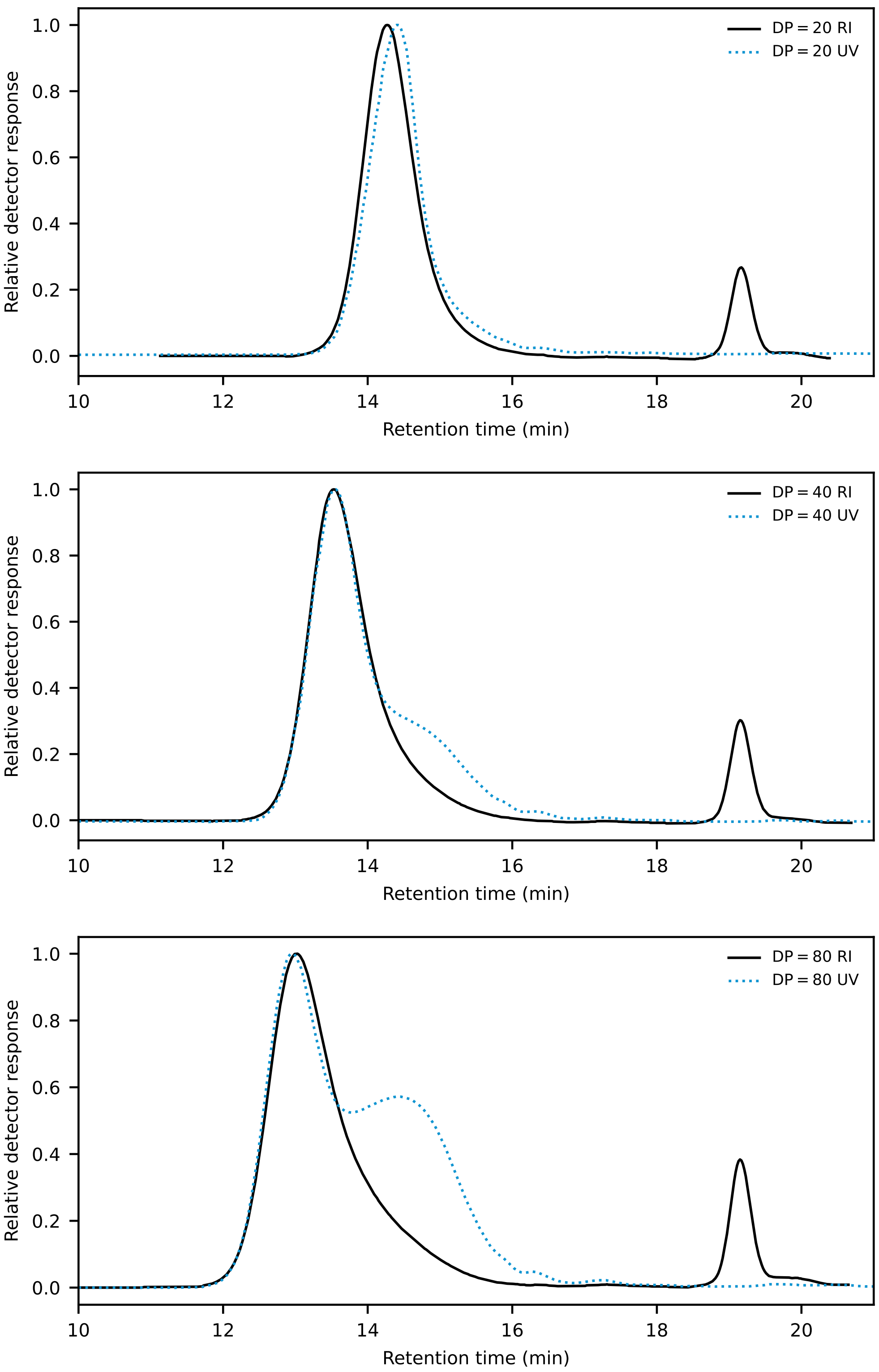

Figure S6: **Overlay of RI and UV (308 nm) SEC chromatograms** of the PAHA polymers with different target degrees of polymerization.

Table S4: Summary of the estimation of the degree of polymerization of PAHA polymers using a combination of monomer conversion data and SEC measurements.

Sample target DP	Monomer conversion	Estimated DP (NMR)	Elution time (main peak RI, min)	Estimated DP (SEC)
DP=20	–	–	14.494	17.9
DP=40	88%	35.2	13.778	–
DP=80	81%	64.8	13.255	–
DP=20^e	72%	14.4	14.72	–

curve using the estimated DP values for the other three samples shown in Table S4. A log(DP) vs elution time graph for these samples yields a straight line ($R^2 = 0.99$) and then a value can be read off for the target DP=20 sample. Further analysis could be carried out by performing, for example, MALDI-TOF (matrix-assisted laser desorption/ionization time of flight mass spectrometry) to measure absolute molar masses for the different polymers.

2.4.5 poly(n-butyl acrylate-*s*-acrylic acid) (PBA-AA)

Unlike the PAHA polymer, whose synthesis is described above, PBA-AA samples were synthesized using a variant of ATRP, namely SET-LRP[S16]. The initiator chosen was not UV active, therefore the PBA-AA^c polymer was modified after synthesis using *click* chemistry to add a coumarin dye. The synthesis begins with the synthesis of poly(n-butyl acrylate-*s*-t-butyl acrylate), followed by the azidification of the polymer chain ends and the addition of the coumarin dye to it. Finally the t-butyl acrylate groups are deprotected to yield the desired HPE. A non-tagged variant of the polymer PBA-AA^0 was also synthesized in identical fashion omitting the chain-end modification steps. The synthesis of all the components is detailed below.

2.4.6 Synthesis of a *clickable* 4MU dye

An alkyne modified 7-hydroxy-4-methylcoumarin (4MU) was synthesized following the procedure described by Chen et al[S22].

To a 250 mL round bottom flask with two-necks 120 mL of acetone was added. Then 7-hydroxy-4-methylcoumarin (5 g, 25.75 mmol, 1 eq.) was added, followed by KI (0.43 g, 2.57 mmol, 0.1 eq.) and K_2CO_3 (7.12 g, 51.5 mmol, 2 eq.) A condenser and an addition funnel were added to the flask and then heated in an oil bath at 60 °C for 45 min. The propargyl bromide (2.9 mL, 25.7 mmol, 1 eq.) was subsequently added dropwise over the period of 1 hour. The reaction mixture was allowed to stir overnight at 60 °C. The reaction mixture was transferred into another flask and 50 mL of DCM was added. This caused a precipitate to form and the supernatant was removed using vacuum filtration. This precipitate was washed two times with a further 50 mL of DCM. The combined DCM supernatants, a gold colored solution, were then extracted two times with an equal volume of deionized water. The organic phase was dried with an excess of $MgSO_4$ and then filtered. The resulting solution was concentrated *in vacuo* and then purified via a recrystallization in anhydrous methanol. Finally the crystals were reprecipitated in cold *n*-hexane, after dissolution in a minimal amount of DCM, to give a white powder. The yield of the reaction was 41%

^{1}H NMR (400 MHz, $CDCl_3$), shown in Fig. S7(a), was carried out to confirm the presence of the desired compound.

2.4.7 SET-LRP synthesis of poly(n-butyl acrylate-*s*-t-butyl acrylate)

The polymerization synthesis to form our initial protected polymer follows from van Ravensteijn et al.[S23].

To a 20 mL vial $CuBr_2$ (17.2 mg, 0.077 mmol, 0.05 eq.) and Me_6TREN (74 µL, 0.28 mmol, 0.18 eq.) were added and then dissolved, with the aid of sonication, in 4.5 mL of TFE. Then n-butyl acrylate (2.79 mL, 19.2 mmol, 12.5 eq.), *t*-butyl acrylate (2.79 mL,

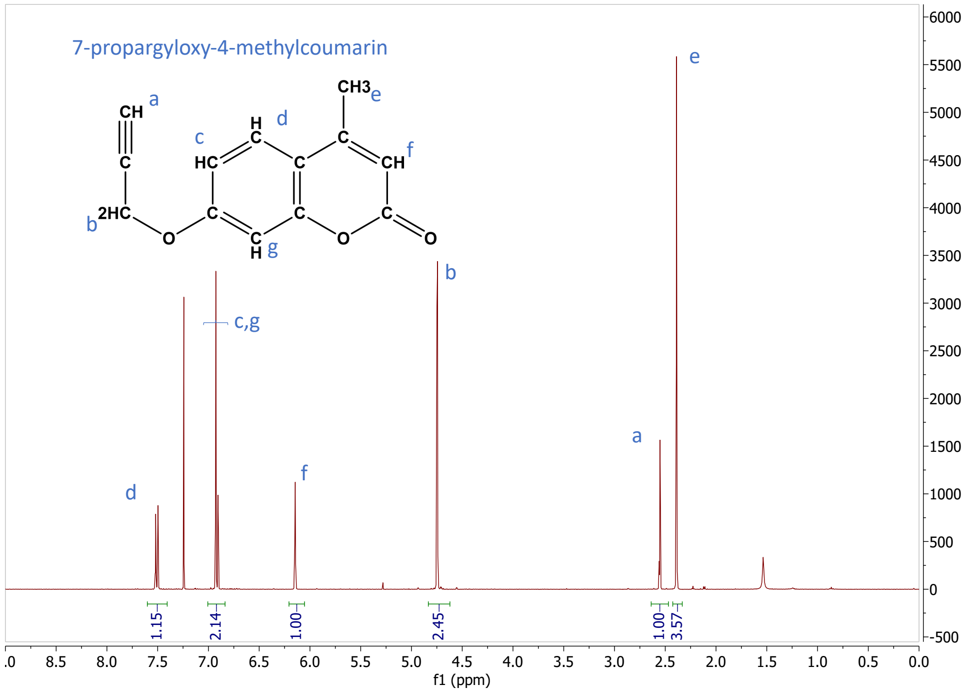

(a) ^{1}H NMR (400 MHz, $CDCl_3$) of the alkyne modified 7-hydroxy-4-methylcoumarin.

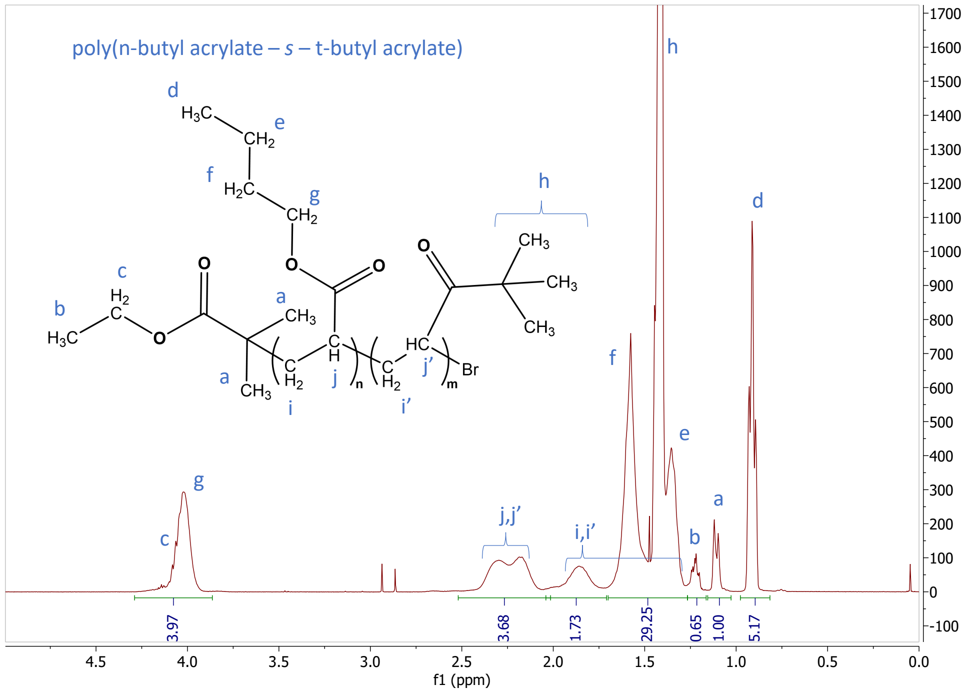

(b) ^{1}H NMR (400 MHz, $CDCl_3$) of the final product of a poly(n-butyl acrylate-*s*-t-butyl acrylate) synthesis (first step of PBA-AA^c synthesis).

Figure S7: ^{1}**H-NMR spectra** of **(a)** the clickable coumarin dye and **(b)** poly(n-butyl acrylate-*s*-t-butyl acrylate).

19.2 mmol, 12.5 eq.) and EBIB (0.023 mL, 1.54 mmol, 1 eq.) were added to the vial. A 10cm length of copper wire, which was previously etched in concentrated HCl, washed with acetone and dried, was wrapped around a stirrer bar and added to the vial. The solution was sealed with a rubber septum and then purged by bubbling dry N_2 through it for 15 min. The reaction was allowed to stir at RT for 5 hours and 40 min. To quench the reaction the mixture was exposed to air and the copper wire laden stirrer bar was removed. The reaction mixture was diluted with DCM and then run through a short basic alumina column to remove any copper salts. The now colorless solution was concentrated *in vacuo*. The polymer was then dissolved in a minimal amount of diethyl ether (approx. 4 mL) and precipitated into a 4 : 1 methanol to water mixture. This process was repeated a further two times. Finally the polymer was dissolved in DCM, dried with excess $MgSO_4$ and concentrated *in vacuo*. Average conversions for this polymerization were 80% and average final yields were 60%.

^{1}H NMR (400 MHz, CDCl3), shown in Fig. S7(b) for PBA-AA^c, was carried out to confirm the presence of the desired compound. Note the broadness of the peaks indicating a polymeric compound.

2.4.8 Azidification of poly(n-butyl acrylate-*s*-t-butyl acrylate)

We can take advantage of the good end group retention of SET-LRP to add an azide moeity to the end of the polymer chains[S16]. This will allow us to *click* on the previously synthesized alkyne-coumarin dye. A procedure from Anastasaki et al.[S24] was the basis of the following procedure.

poly(n-butyl acrylate-*s*-t-butyl acrylate) (3.2 g, 1.25 mmol, 1 eq.) was dissolved in 30 mL of DMF. Then NaN_3 (0.813 g, 12.5 mmol, 10 eq.) was added to the solution and the whole reaction mixture was stirred at RT overnight. The now cloudy solution was diluted with 60 mL of $CHCl_3$ and extracted with 400 mL of water. The organic phase

was separated and the water phase was washed two times with 100 mL of $CHCl_3$. The combined organic phases were then washed two times with 100 mL of water. Finally the organic phase was dried using $MgSO_4$ and concentrated *in vacuo*. The average yields for this procedure were approx. 60%.

^{1}H NMR (400 MHz, $CDCl_3$) for PBA-AA^c, was carried out to attempt to confirm the presence of the desired compound. Only a small change in the NMR spectra is expected, namely the shift in the proton resonance of the terminal C-H group. Small peaks are found at 3.6-3.8 ppm which were not present before the azidification. We may attribute this to the azidification of the chain-end but the spectra is too noisy to qualitatively look at coupling efficiencies.

2.4.9 Coumarin dye - polymer chain end *click* reaction

The previously synthesized alkyne-coumarin dye can now be coupled to the end of the polymer. The following procedure was adapted from Honda[S25].

CuBr (0.252 g, 1.76 mmol, 1.5 eq.) and PMDETA (0.368 mL, 1.76 mmol, 1.5 eq.) were dissolved in 30 mL of DMF with the aid of sonication. This resulted in a dark green solution. Then, both of the previously synthesized 7-propargyloxy-4-methylcoumarin (0.502 g, 2.34 mmol, 2 eq.) and azidified poly(n-butyl acrylate-*s*-t-butyl acrylate) (3.0 g, 1.17 mmol, 1 eq.) were dissolved in the solution. The solution was sealed with a rubber septum and then purged by bubbling dry N_2 through it for 45 min. The solution was allowed to stir overnight. The whole reaction mixture was diluted in acetone and then passed through a silica column to remove the copper salts. The solution was then concentrated *in vacuo*. The remaining sample was dissolved in 15 mL of THF and then precipitated into a 4 : 1 methanol to water mixture, with a ratio of 7.5mL of THF solution to 40 mL of methanol-water. This precipitation was repeated a further two times dissolving the precipitated polymer in a minimal amount of THF (approx. 2 mL). The polymer was then dissolved

in 20 mL of DCM and the solution was dried over excess $MgSO_4$. Finally the solution was concentrated *in vacuo* to yield a clear polymer. The average yields for this procedure were around 65% with coupling efficiencies (calculated using ^{1}H NMR) of the dye to the end of the polymer of approximately 60%[2].

^{1}H NMR (400 MHz, $CDCl_3$) for PBA-AA^c, shown in Fig. S8(a), was carried out to confirm the presence of the desired compound.

2.4.10 poly(n-butyl acrylate-*s*-t-butyl acrylate) deprotection

The final step to yield a hydrophobic polyelectrolyte is to deprotect the t-butyl ester groups on the polymer chain into carboxylic acid groups. The procedure described below was used to synthesize the final PBA-AA^c. Synthesis of PBA-AA^0 followed the same procedure at a 2/3 scale.

The, still protected, coumarin-modified polymer (2.1 g, 0.82 mmol, 1 eq.) was dissolved in 15mL of DCM in a round-bottom flask. Then trifluoroacetic acid (6.3 mL, 82 mmol, 100 eq.)[3] was added and the solution was allowed to stir overnight. The solution was then concentrated *in vacuo*. The remaining sample was dissolved in 20 mL of THF and then precipitated into a cold hexane, with a ratio of 5 mL of THF solution to 40 mL of hexane. This was repeated a further three times using a minimal amount of THF to dissolve the precipitated polymer each time. Finally, the precipitated polymer was dissolved in excess ethanol and concentrated *in vacuo*. The average yields for this procedure were approx. 65% with deprotection efficiencies (calculated using ^{1}H NMR) being close to 100%. Removal of any excess trifluoroacetic acid was qualitatively confirmed using ^{19}F NMR.

[2]This efficiency is calculated with respect to the chain end, therefore assuming there is full azide coverage of it.

[3]The amount of trifluoroacetic acid to be added was calculated as 10x the amount of t-butyl acrylate groups in solution. There were approximately 10 *t*-butyl acrylate groups per polymer chain, therefore leading to an approximately 100x equivalency. This could also simply be stated as excess trifluoroacetic acid.

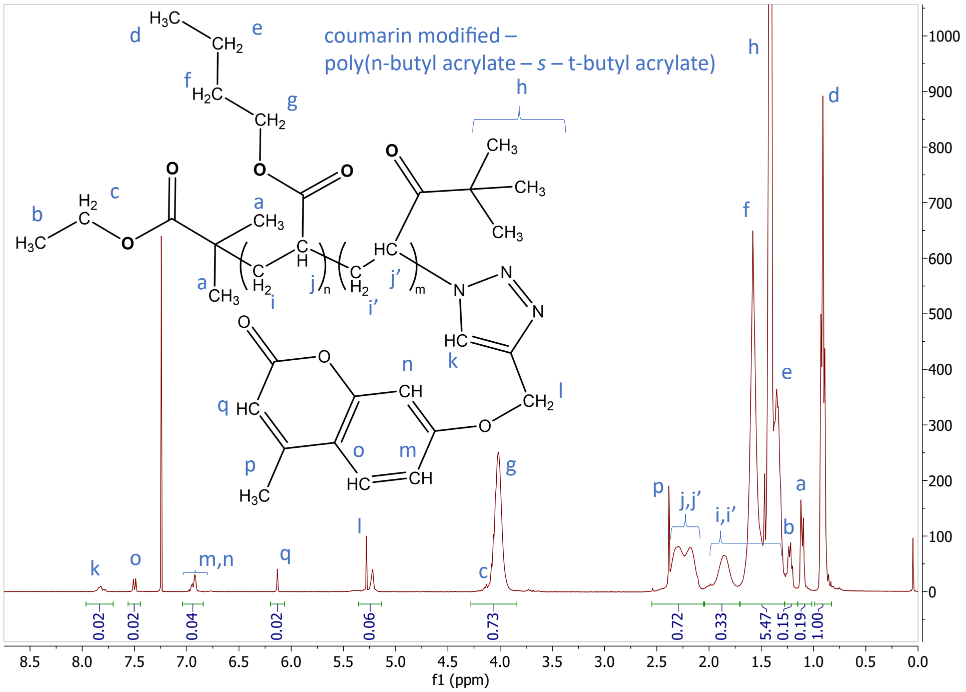

(a) ^{1}H NMR (400 MHz, $CDCl_3$) of the coumarin-modified poly(n-butyl acrylate-*s*-t-butyl acrylate)

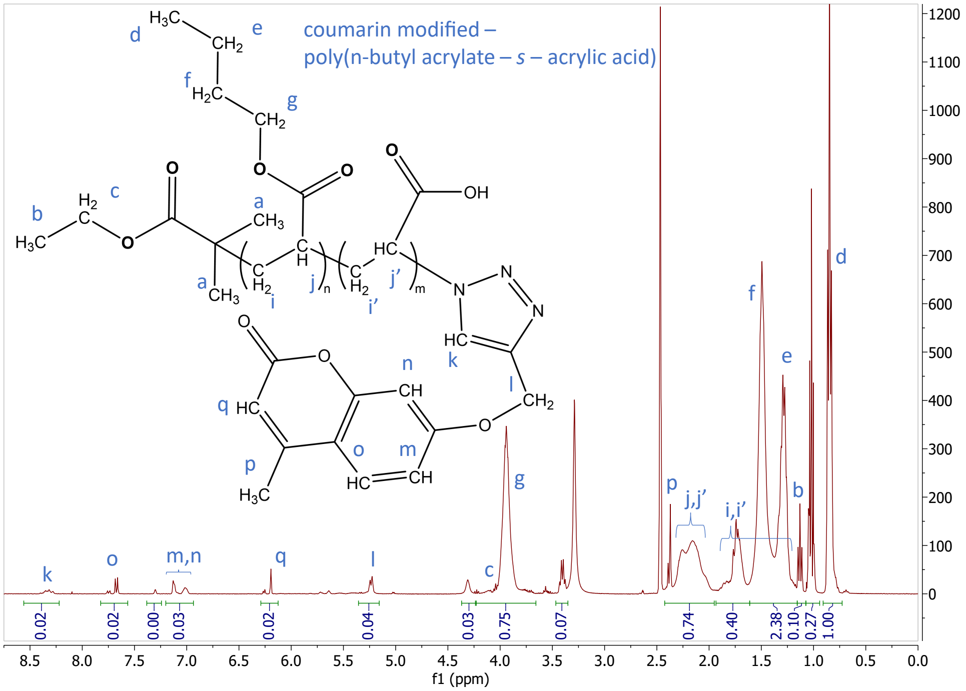

(b) ^{1}H NMR (400 MHz, DMSO-d6) of the coumarin-modified poly(n-butyl acrylate-*s*-acrylic acid)

Figure S8: ^{1}**H-NMR spectra** of the protected and deprotected coumarin-modified random copolymer HPE.

2.4.11 PBA-AA polymer characterization

^{1}H NMR (400 MHz, DMSO-d6), shown in Fig. S8(b) for PBA-AA^c, was carried out to confirm the presence of the desired compound and SEC was used to investigate the length distribution.

The NMR spectra shows the disappearance of the t-butyl group resonance at around 1.4 ppm, indicating the full deprotection of the polymer. Overall, the appearance of the spectra is similar to the protected polymer indicating that the rest of the structure of the molecule has remained unchanged.

Unlike with the PAHA polymer discussed earlier in this section it is possible to extract the degree of polymerization of the polymer directly from the NMR spectrum of the initial protected polymer (Fig. S7(b)). Using the reference peak at 1.1 ppm, which we attribute to the two methyl groups on the initiator moiety, and comparing this integration to the integrated peak of the backbone $C-H_2$ protons on the polymer allows us to calculate an estimated degree of polymerization for the whole polymer. Further, using the resonance peak of the terminal $C-H_3$ group at around 0.9 ppm on the n-butyl monomer allows us to calculate the ratio of t-butyl to n-butyl in the initial protected polymer. It is expected that this ratio will be retained for any subsequent polymer modifications. The calculated values for the total degree of polymerization, and the individual n- and t-butyl degrees of polymerization for both polymer variants synthesized are summarized in Table S5

Table S5: Summary of the estimation of the degree of polymerization and dispersity of the PBA-AA polymer samples.

Sample	Total DP (NMR)	n-butyl acrylate DP (NMR)	t-butyl acrylate / acrylic acid DP (NMR)	Đ (SEC)
PBA-AA^c	22.1	10.3	11.8	1.09
PBA-AA^0	20.7	9.9	10.6	1.11

Analysis of the length dispersity (Đ) of this polymer is essential for the discussions we

carry out in the results section of this study. The SEC of the PBA-AA^c sample carried out in ammonium acetate buffer (300 mM, pH 9) shows a dispersity value of around 1.09, with a marked tail. It is worth noting however that the chemical dispersity of this polymer, due to the two similarly reactive monomers which compose it, might lead to a spread in the elution times during SEC. This is a result of the fact that the two randomly arranged groups are quite chemically distinct and therefore their interaction with the aqueous buffer will differ, leading to different apparent weights. If we assume that an identical polymerization of the homopolymer poly(n-butyl acrylate) has the same length dispersity, previous experiments not detailed here show a dispersity value of 1.04 and a SEC trace without a tail.[4]

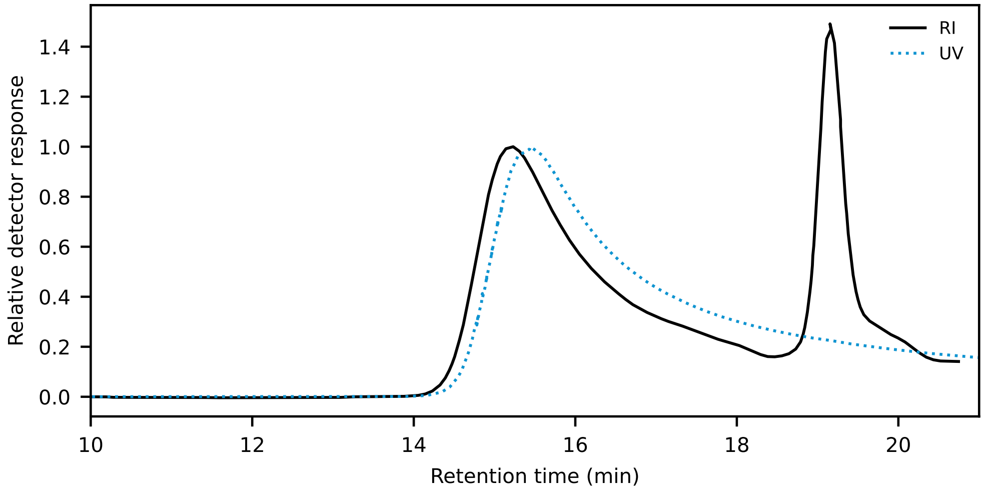

Figure S9: **SEC chromatograms** of the final product of the PBA-AA^c synthesis.

2.5 Measurement of HPE partitioning and ionization behavior

The individual data treatment for each type of experiment was kept as consistent as possible to allow for a fair comparison. The individual raw data is now presented for each of the

[4]It is worth calculating the difference in the standard deviation of the distributions that this, apparently small, difference in $Đ$ actually represents. The standard deviations for $Đ$ values of 1.04 and 1.09 are approx. 3.46 and 6 respectively, where have assumed an average length of 20 units for the polymer. Therefore the change in the implied distribution is not negligible.

experiments shown in the main text accompanied by a general explanation of the data analysis carried out.

2.5.1 Measuring the fraction of chains in the hydrophobic phase

Data treatment for the extraction of data from UV-Vis absorbance measurements is straightforward. First a blank measurement of the solvent is taken, which in this case is water-saturated pentanol. Then measurements of all the samples are carried out in triplo (or duplo). After subtraction of the blank signal and a further background subtraction is carried out, the relative intensity of the characteristic wavelength of the particular absorbing moiety of each polymer type is normalized with respect to the stock solution of the polymer used for each experiment. This allows for the identification of any anomalous data. The data is then rescaled to set the highest absorption value, which will be at the lowest pH values, to 1 as we expect all of the chains to reside in the hydrophobic phase at these pH values. The lowest fraction value, if there is a plateau in the data, are set to 0. A linear relationship between concentration and absorbance (Beer-Lambert law) has been assumed throughout. An example of this procedure is shown for one of the sets of raw data following from this introduction.

2.5.1.1 PAHA (DP=18): f_H from titration experiment

Figure S10 (a) shows the raw UV-Vis absorbance data for the pentanol phase of a two-phase pentanol and water system which is titrated as described in the last section. The experimental data is shown in 2(b).

Part (b) of the figure shows the absorbance spectrum for the water saturated pentanol used as the hydrophobic phase in this experiment. Part (c) shows the spectra after the solvent spectrum is removed and part (d) after the background signal found between 375 and 400 nm is removed. The vertical dashed line indicates the wavelength (308 nm) which

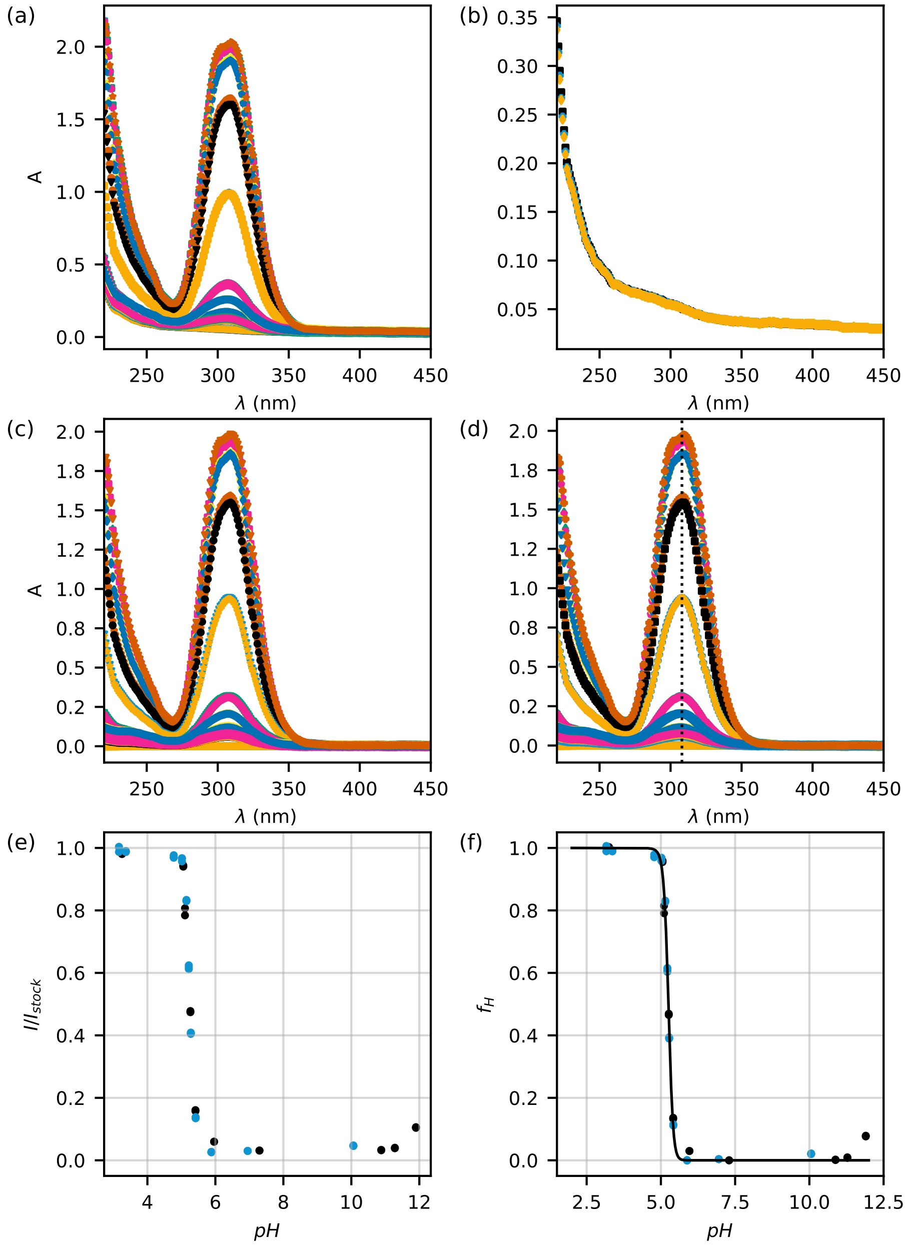

Figure S10: **Example data treatment for UV-Vis absorption data.** **(a)** Raw UV-Vis data from the hydrophobic phase from a single experimental run.**(b)** Spectrum of the solvent used as hydrophobic phase. **(c)** Spectra of the hydrophobic phases with the solvent signal removed. **(d)** Spectra after background removal. **(e)** Normalized intensity of the chosen wavelength with respect to the stock solution of polymer for both experimental runs. **(f)** Final data after rescaling.

is used as the reference wavelength to calculate relative concentrations with respect to the stock solution. These relative concentrations are plotted in part (e). Finally we subtract the minimum absorbance and renormalize to the highest one is part (f).

2.5.1.2 PBA-AA^c: f_H from buffered experiment

Figure S12, presents in the top four panels the raw UV-Vis spectra, the blank solvent spectrum, the raw data and the scaled data for the buffered partitioning experiment of PBA-AA^c detailed in Fig. 4. It is worth noting here that the high baseline at high pH values. It is possible that there is residual dye, not coupled to the polymer chain end, that leads to this high baseline. In this case a series of refractive index measurements could determine if the baseline is due to dye impurities or the polymer itself.

2.5.2 Measuring the HPE ionization fraction from titration experiments

The procedure to extract the ionization fraction of a HPE from the titration of hydrophobic polyelectrolytes relies on the necessary electroneutrality of the solution. This condition allows for the following equality to be set up:

$$[\text{ionized acidic groups}]=[\mathrm{H}^+] - [\mathrm{OH}^-] + [\mathrm{K}^+]_{\text{added}} - [\mathrm{Cl}^-]_{\text{initial}} \tag{33}$$

where the initial concentration of Cl^- originates from the initial acidification of the aqueous phase of the two-phase system using HCl and the added concentration of K^+ is the concentration of potassium ions added when titrating using KOH. The concentration of H^+ and OH^- is found from the pH of the aqueous solution and follows from $[\mathrm{H}^+] = 10^{-pH}$ and $pK_w = pH + pOH$.

Similarly to the procedure to find the fraction of chains in the hydrophobic environment, we rely on scaling of the data to eliminate artifacts that arise throughout the experiments. In this case it is assumed that at the starting point of the titration (pH 3) all of the

polymer resides in the oil phase (following from the UV-Vis absorption data) and therefore no ionization should be present. Although, a priori, it is possible to estimate the number of ionizable groups added to the experiment system, the accuracy of this prediction is not very high. This is due to the potential residual solvent and/or water swelling the polymer samples used, which skew any potential calculation relying on masses. Therefore the values of the ionization fraction at high pH values are also set to 1 as to reflect the expect full ionization.

2.5.2.1 PAHA (DP= 18): θ from titration data

Figure S11 illustrates the procedure described above hor the PAHA (DP= 18) titration presented in Fig. 2.

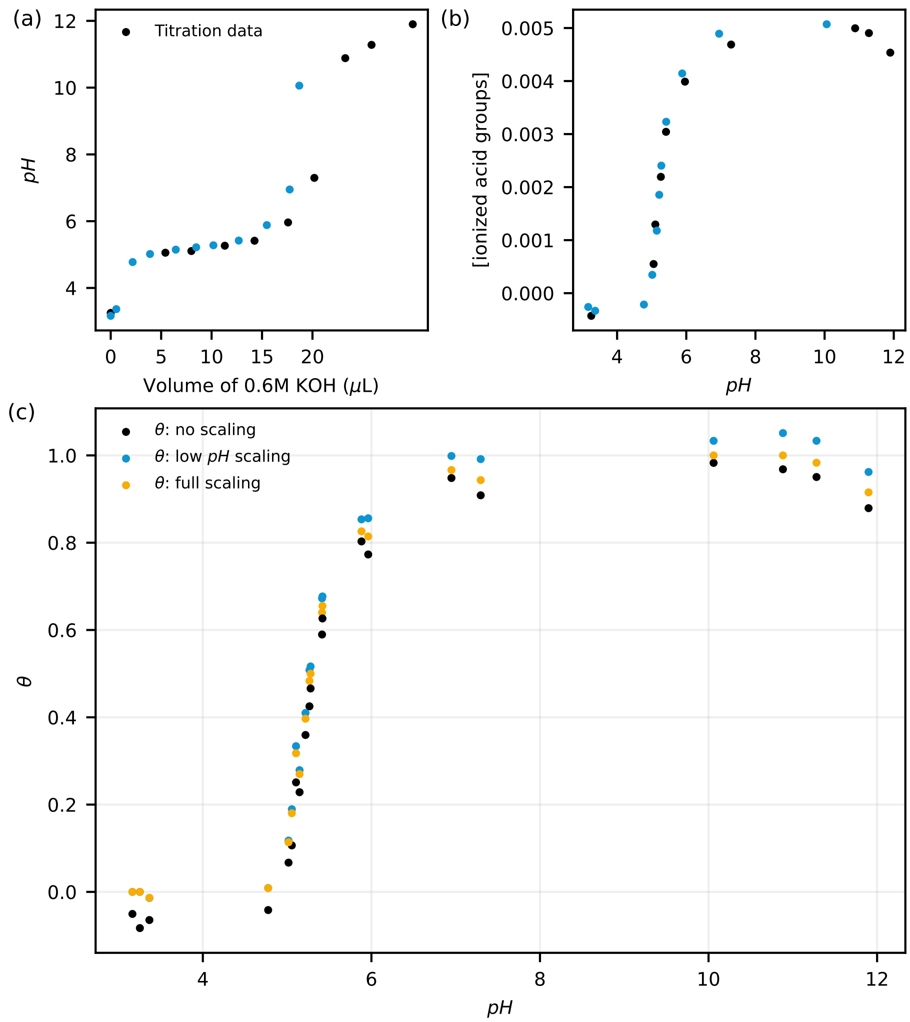

Figure S11: **Example data treatment for PAHA titration data. a)** Raw titration data for both experimental repeats. **b)** Concentration of ionized sites calculated from Eq. (33). **c)** Ionization fraction data calculated with three different scaling methods: no scaling, scaling to force no ionization at the lowest pH value and scaling that additionally sets the ionization to 1 for the highest value of the ionization.

2.5.2.2 PBA-AA^0: titration and buffered experiments

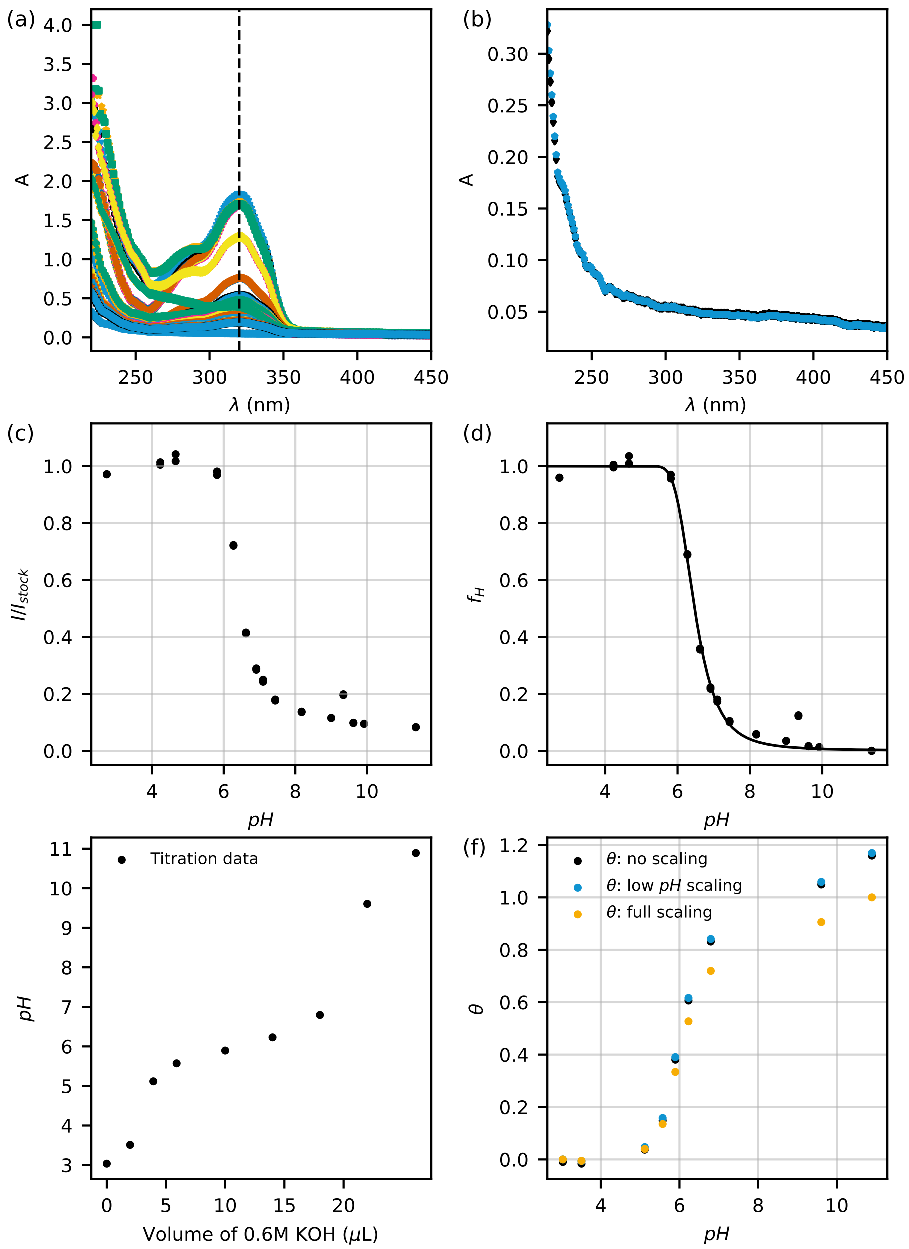

Figure S12: **Raw data for PBA-AA partitioning. a)** Raw UV-Vis data of PBA-AA^c from the hydrophobic phase of a buffered two-phase oil and water system. **b)** Spectrum of the solvent used as hydrophobic phase. **c)** Normalized intensity of the chosen wavelength with respect to the stock solution of polymer. **d)** Final f_H data after rescaling. **e)** Raw titration data of PBA-AA^0 in a two-phase oil and water system. **f)** Ionization fraction data calculated with three different scaling methods: no scaling, scaling to force no ionization at the lowest pH value and scaling that additionally sets the ionization to 1 for the highest value of the ionization.

Figure S12 presents, in the bottom two panels, the data treatment for the titration experiments carried out on PBA-AA^0 detailed in Fig. 4. Of note is this case is the lack of plateau at high values of ionization, as well as the values over unity for the unscaled ionization. There are no repeats carried out for this set of data, and therefore it is possible there is an erroneous experimental offset for the measurement of the volume of KOH added which we are not aware of. The transition-pH value does match the independent buffered experiment so there is a qualitative agreement with the buffered system but repeats of the experimental runs are necessary to confidently describe the system.

3 Oligomeric metal chelates (OMC)

3.1 Materials

opper (II) bromide ($CuBr_2$, 99%), tris[2-(dimethylamino)ethyl] amine (Me_6TREN, 97%), ethyl α-bromoisobutyrate (EBiB, 98%), silica gel (high-purity grade (Davisil grade 633), pore size 60Å, 200-425 mesh particle size), 2,2':6',2"-terpyridine (T, 98%), 4'-chloro-2,2':6',2"-terpyridine (Cl-T, 99%), methanol-d_4 (99.8%), dimethyl sulfoxide (DMSO, anhydrous 99.9%), iron (II) chloride tetrahydrate ($FeCl_2$, 99%), L-ascorbic acid (L-AA), dimethyl sulfoxide-d_6 (DMSO-d_6, 99.9%), ethanolamine (99.5%), N-(3-dimethylaminopropyl)-N'-ethylcarbodi-imide hydrochloride (EDC-HCl, 98%), N,N-dimethyl- formamide (DMF, 99.8%), and N-hydroxysuccinimide (NHS, 98%) were purchased from Sigma Aldrich (St. Louis, MO, USA). 2,2,2-trifluoroethanol (TFE, 99%), potassium hydroxide powder (KOH, $\sim$ 85%), sodium chloride (NaCl) and sodium hydroxide pellets (99%) were purchased from Merck (Burlington, MA, USA).

Dichloromethane (DCM, 99%), methanol absolute HPLC (MeOH, 99.9%), tetrahydrofuran (THF, 99.8%), diethyl ether (Et_2O, anhydrous 99.5%), chloroform HPLC (99.9%) and acetonitrile (ACN, HPLC-R) were purchased from Biosolve B.V. (Valkenswaard, The

Netherlands). Copper wire (Cu(0), d = 0.25mm, 99.99%) was purchased from Alfa Aesar (Haverhill, MA, USA). Hydrochloric acid (HCl, 37%) was purchased from Acros Organics (Geel, Belgium). Ethanol absolute (100%), acetone (100%), n-hexane (99%) and triethylamine (TEA, high purity grade) were purchased from VWR International (Radnor, PA, USA). *tert*-butyl acrylate (*t*BA) and n-butyl acrylate (nBA) were purified (using a 1:1 Al_2O_3:Silica column) and stored at 4 °C. Trifluoroacetic acid (TFA, 99%), aluminum oxide (98%) and sodium sulfate (Na_2SO_4, 99%) were purchased from Honeywell (Charlotte, NC, USA). Chloroform-d1 (99%) was purchased from Carl Roth (Karlsruhe, Germany). Dimethyl sulfoxide (DMSO, 99.7%) was purchased from Thermo Fisher Scientific (Waltham, MA, USA). The Milli-Q (MQ) water used was deionized by a Millipore Synergy water purification system (Merck Millipore, Billerica, MA, USA).

3.2 Synthesis of the terpyridine-functionalized polymer (PT)

The synthesis of the terpyridine-functionalized polymers was done in multiple steps. As an overview, illustrated in Figure S13, the synthesis starts with synthesizing poly (*tert* - butyl acrylate) and deprotecting it into poly(acrylic acid) (PAA).

Subsequently, EDC/NHS activation was done on the carboxylic acid groups of PAA in order to synthesize poly (N-hydroxysuccinimide) (PNHS). To functionalize (PNHS) with terpyridine, Cl-T was synthesized with ethanolamine to yield an amine functionalized terpyridine, 2-(2,2':6',2"-terpyridine-4'-yloxy) ethylamine (ET), which was necessary in order to substitute the NHS groups on the polymer via nucleophilic substitution. Eventually, the terpyridine-functionalized polymers were synthesized and coded as PT16 with structure (5), shown in Figure S13. Number 16 shows the number of monomer units. After each synthesis step, the product was characterized using the same instruments as described the Instrumentation Section for the HPE study.

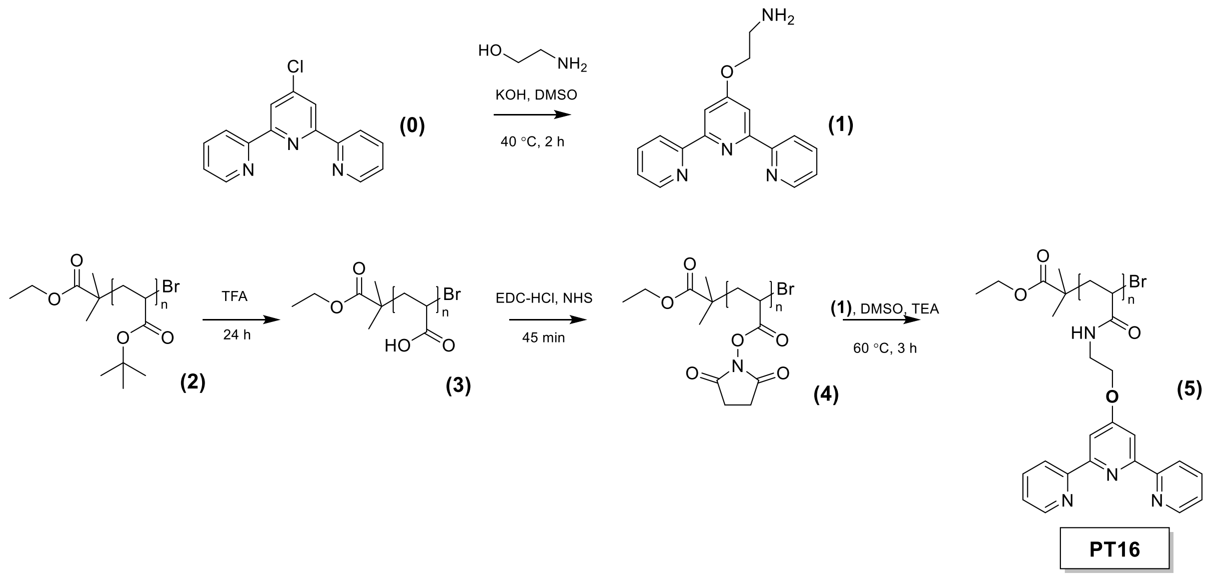

Figure S13: **Synthesis overview.** Steps of synthesizing the terpyridine-functionalized polymer with 16 repeating units (PT16).

3.2.1 Synthesis of 2-(2,2':6',2"-terpyridine-4'-yloxy) ethylamine (ET) (1)

Cl-T (267.71 mg, 1 mmol, ^{1}H-NMR is shown for comparison in FigureS14) and ethanolamine (67.17 μl, 1.1 mmol) were added to a suspension of powdered KOH (280.55 mg, 5 mmol) in DMSO (5 ml) and stirred at 40 °C for 2 h. The reaction mixture was then added to 40 ml of DCM and washed with MQ water (3 $\times$ 40 ml) by liquid-liquid extraction. The DCM solution was dried over Na_2SO_4 and the solvent was removed. 2-(2,2':6',2"-terpyridine-4'-yloxy) ethylamine (ET) was obtained as a light yellow solid and used subsequently without further purification (252.60 mg, 86.5% yield).^{1}H-NMR (400 MHz in Chloroform-d_1) is shown in Figure S15).

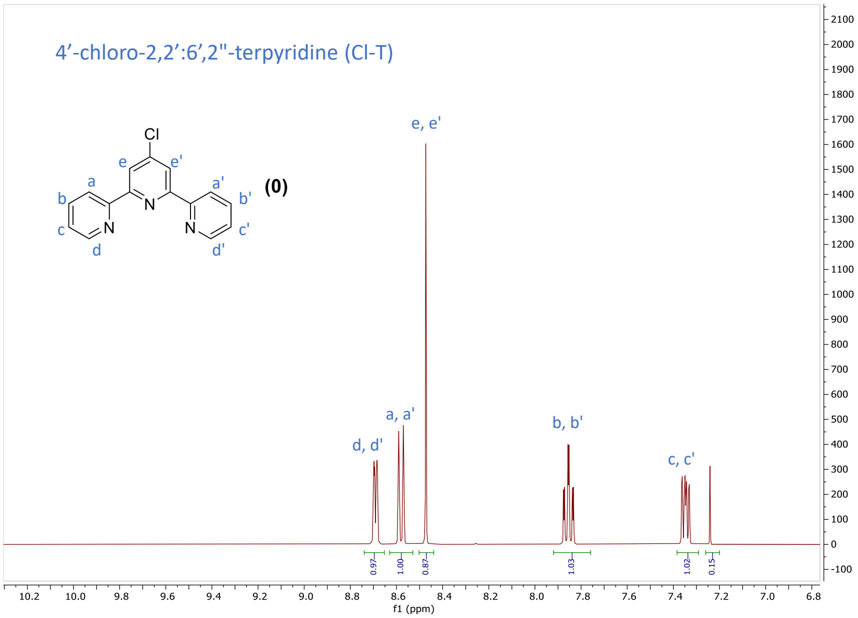

Figure S14: **^{1}H-NMR spectrum** of Cl-T (0).

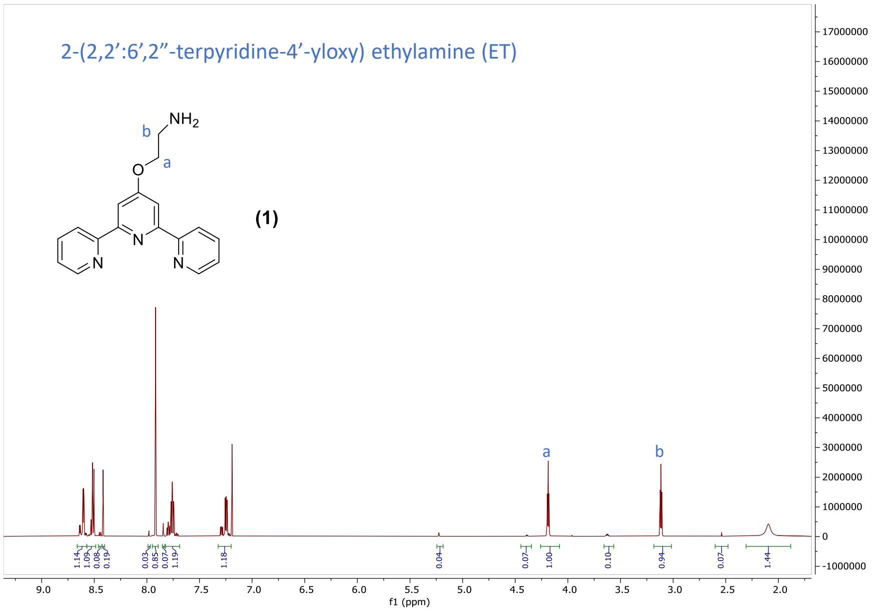

Figure S15: **^{1}H-NMR spectrum** of ET (1).

3.2.2 SET-LRP Synthesis of poly(*tert*-butyl acrylate) (P*t*BA) (2)

$CuBr_2$ (7.59 mg, 0.034 mmol, 0.05 equiv. per initiating sites), TFE (1 ml) and Me_6TREN (33 μl, 0.123 mmol, 0.18 equiv. per initiating sites) were added in a 4-ml vial and the resulting solution was treated with ultrasonication for 30 minutes using a CPX8800H ultrasonic cleaning bath (Branson Ultrasonics™, Brookfield, CT, USA). For Cu(0) source, 5cm of 0.25 mm thick Cu copper wire was cut and etched in HCl for 30-45 minutes. Afterwards, the wire was washed with acetone and dried with dust-free paper (three times). *t*BA (2ml, 13.7mmol, 20 equiv.) was added, together with the initiator, EBiB (101 μl, 0.685 mmol, 1 equiv.) to the mixture and the reaction was activated by adding a magnetic stir bar wrapped by the copper wire. The reaction was degassed under N_2 flow for the first 20 min was left for 4 hours (in total). The product was then diluted with Et_2O, and purified by passing through a 1:1 Al_2O_3:Silica column. Consequently, the product was dissolved in minimal amount of DCM and washed 4 times by a 4:1 MeOH:water solution by centrifugation at 3273 $\times$ g for 15 minutes using an Allegra X-12R Centrifuge (Beckman Coulter, Brea, CA, USA). Finally, the product was dried under N_2 flow and vacuum. The resulting material was a sticky viscous gel (80% conversion, DPn $\approx$ 16).^{1}H-NMR (400 MHz in Chloroform-d_1) and SEC chromatogram of the final product are shown in Figure S16 and S17, respectively.

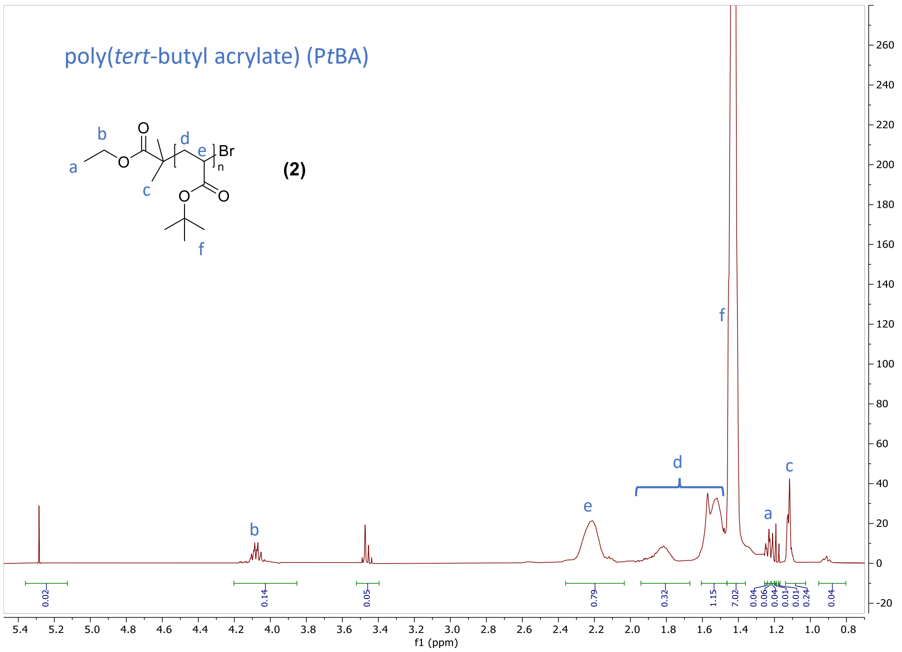

Figure S16: **^{1}H-NMR spectrum** of P(tBA) (2).

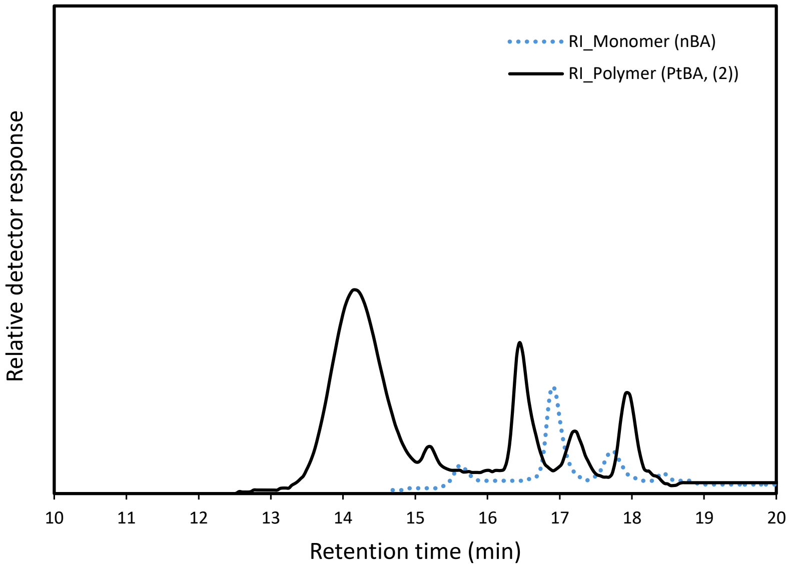

Figure S17: **SEC chromatograms** of PtBA (2, solid black line), compared to BA (monomer, dotted blue line).

3.2.3 Deprotection of (2) into poly(acrylic acid) (PAA) (3)

In a 4-ml vial, compound (2) (256.64 mg), TFA (2.6 ml) and a magnetic stir bar were added and left under stirring for 24 hours. Next, the mixture was diluted with minimal amount of DCM, added to an excess amount of Et_2O and centrifuged at 3273 × g for 15 minutes. This was repeated three times. After centrifugation, the product was dried overnight and a white powder was obtained (≈ 80% yield). ^{1}H-NMR (400 MHz, methanol-d_4) is shown in Figure S18.

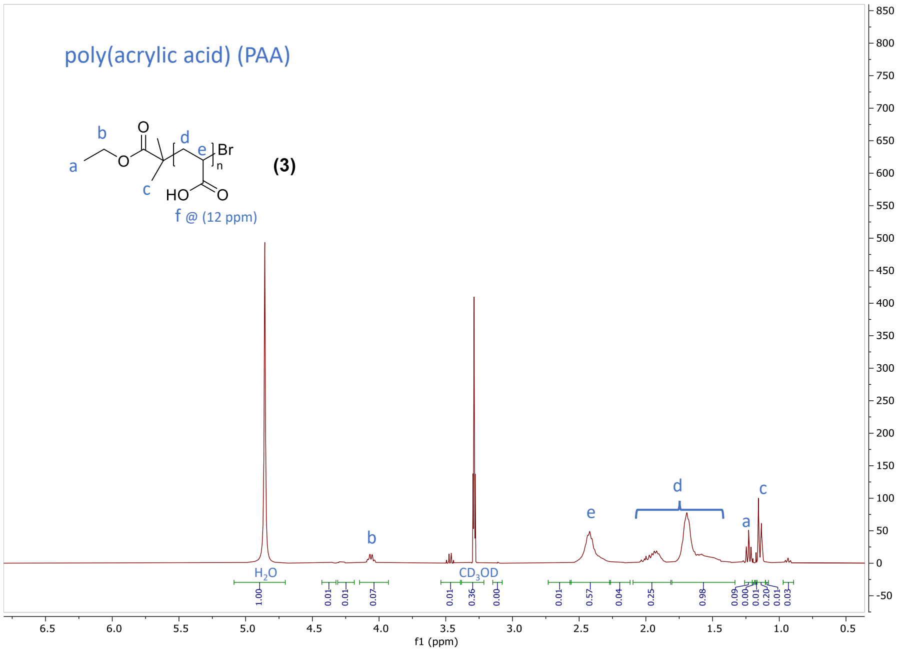

Figure S18: **^{1}H-NMR spectrum** of PAA (3).

3.2.4 EDC/NHS Activation of (3) into PNHS (4)

Compound (3) (47.55 mg, 0.057 mmol) was added together with EDC-HCl (164.24 mg, 0.857 mmol, 1.5 equiv. per carboxylic acid groups) and NHS (98.60 mg, 0.857 mmol, 1.5 equiv. per carboxylic acid groups) in a 20-ml vial. Afterwards, 8.57 ml MQ water

was added to have 0.1 M EDC-HCl / NHS in water. The mixture was stirred at room temperature for 45 minutes. Consequently, the mixture was added to 35ml MQ water, vortexed vigorously and centrifuged at 3273 × g for 20 minutes. This was repeated 3 times. The precipitate was dried overnight under a flow of N_2. The outcome was a white powder (51.73 mg, 49.5% yield). ^{1}H-NMR (400 MHz, acetone-d_6) is shown in Figure S19

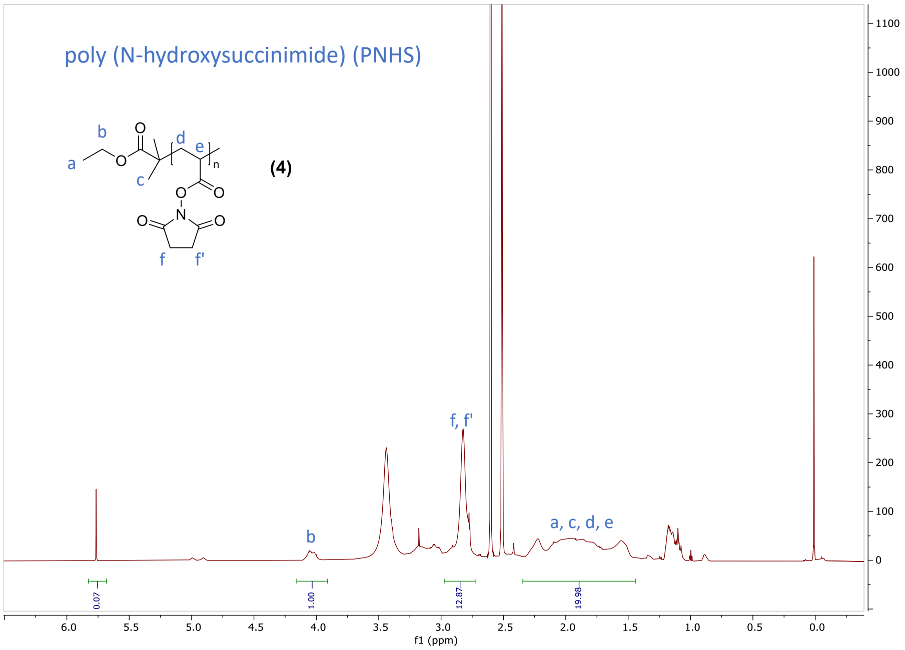

Figure S19: **^{1}H-NMR spectrum** of PNHS (4).

3.2.5 Functionalization of PNHS (4) with ET (1) to get PT16 (5)

In a 4-ml vial, compound (4) (30 mg, 0.010 mmol) and compound (1) (72.51 mg, 0.25 mmol, 1.5 equiv. per NHS group) were added, following by addition of a magnetic stir bar. The vial was sealed and degassed under N_2 flow. Next, anhydrous DMSO (0.3 ml) was added, and the mixture was stirred until it became homogeneous. Afterwards, 1 drop of TEA was added and the vial was kept in an oil bath for 3 hours at 60 °C. During the reaction the solution became red. Consequently, the product was precipitated in 20 ml ice

cold acetone, and then centrifuged at 3273 × g for 30 minutes. The precipitate red gel was left overnight under N_2. The final product was a red gel-like solid (17.3 mg, ≈ 30% $Đ \approx 1.04$). ^{1}H-NMR (400 MHz, acetone-d_6) is shown in Figure S20.

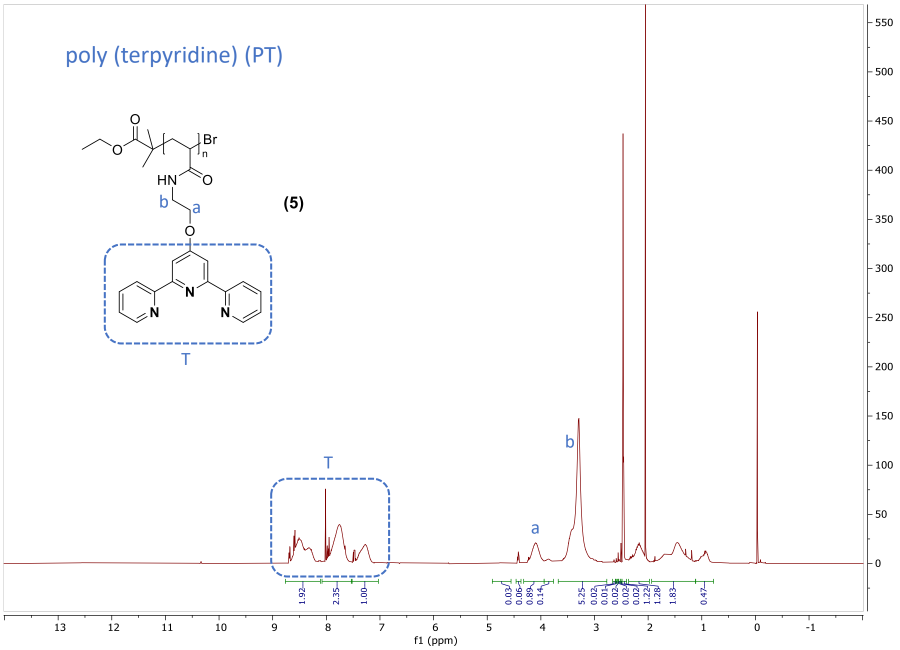

Figure S20: **^{1}H-NMR spectrum** of PT (5).

3.3 Monitoring the binding behavior of iron onto terpyridine in a two-phase water and oil set-up

In order to monitor the binding behavior of the iron ions onto terpyridine monomer and the terpyridine-functionalized polymers, a two-phase water/oil set-up was designed, Figure 1. The oil phase was chosen to be dichloromethane (DCM) due to its immiscibility with water. Solutions of terpyridine monomer or terpyridine-functionalized polymers in DCM with the same concentration of terpyridine groups (200 μM) were prepared. In addition, the aqueous phase contained various concentrations of $FeCl_2$ ranging between 10 and 200

μM. To ensure the reduction of the iron ions to Fe^{2+}, an excess amount of ascorbic acid (AA) was added to the water solutions. The described water and oil solutions were then exposed to each other in sealed vials and kept for 10 days after which the color of the aqueous phase in the vials turned pink. In the case of terpyridine-functionalized polymers, gel-like polymer networks were observed in the aqueous phase which is explained by the formation of bis (terpyridine) iron (II) complexes and therefore formation of crosslinked polymer chains with iron bridges. Finally, the water and oil phases were isolated and analyzed for iron and terpyridine concentrations, respectively (See Figure 3 for summary of the results).

3.3.1 Quantification of the free iron concentration in the water phase by inductively coupled plasma - atomic emission spectroscopy (ICP-AES) and ultraviolet-visible light spectroscopy (UV-Vis)

The free iron concentration was obtained by subtraction of the iron in chelate complexes with terpyridine groups (i.e., bound iron ions) from the total iron in the water phase (i.e., bound + free iron ions). Therefore, free iron (i.e., not chelated by terpyridine) concentration in the water phase was measured by the difference between the measured iron concentrations with ICP-AES and UV-Vis spectroscopies. ICP-AES was performed on the solutions using an Optima 8300 instrument (PerkinElmer, Waltham, MA, USA) to obtain the total iron concentration in the water phase. Samples were dissolved in 10 ml of a 2% HNO_3 solution to achieve optimal measurement concentration ranges. The measurements were performed in triplicate and the results were reported by an average and a standard deviation from duplicate measurements. UV-Vis measurements were done on the solutions at $\lambda_{max} = 552$ nm at room temperature by a Lambda-35 spectrophotometer (PerkinElmer, Waltham, MA, USA), using quartz cuvettes. All measurements were performed in separate duplicates and quantification of the bound iron was performed with a calibration curve on

a similar system (0.0125 – 0.3 mM, $R^2 > 0.99$), see Figure S21.

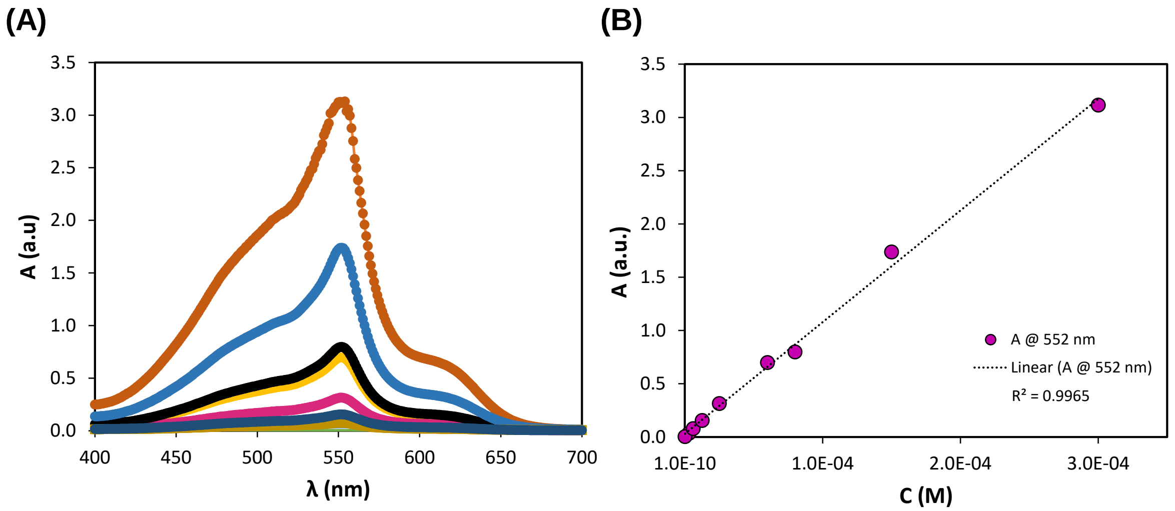

Figure S21: **Calibration used for quantification of the bis iron (II) terpyridine complexes in the aqueous state.** (A) Raw UV-Vis data of bis iron (II) terpyridine, $(FeT_2)^{2+}$ complexes in the water phase. (B) Calibration curve of the $(FeT_2)^{2+}$ complexes used for quantification of bound iron in the water phase (aqueous state).

3.3.2 Quantification of terpyridine/terpyridine-functionalized polymers in the oil phase by ultraviolet-visible light spectroscopy (UV-Vis)

The concentration of terpyridine groups (i.e., also indicative of the concentration of the polymers) in the oil phase was quantified by UV-Vis with $\lambda_{max} = 279$ nm at room temperature. The oil solutions were mixed with acetonitrile (ACN) (1:1 volume ratio) prior to measurement, following a previously-reported procedure[S26,S27]. UV-Vis spectra of the solutions were then recorded on a Lambda-35 spectrophotometer (PerkinElmer, Waltham, MA, USA), using quartz cuvettes. All the measurements were done in separate duplicates and quantification of the remained terpyridine fraction in the oil phase was performed with a calibration curve of terpyridine solutions in DCM/ACN with 1:1 volume ratio (0.00625 – 0.2 mM, $R^2 > 0.99$), see Figure S22.

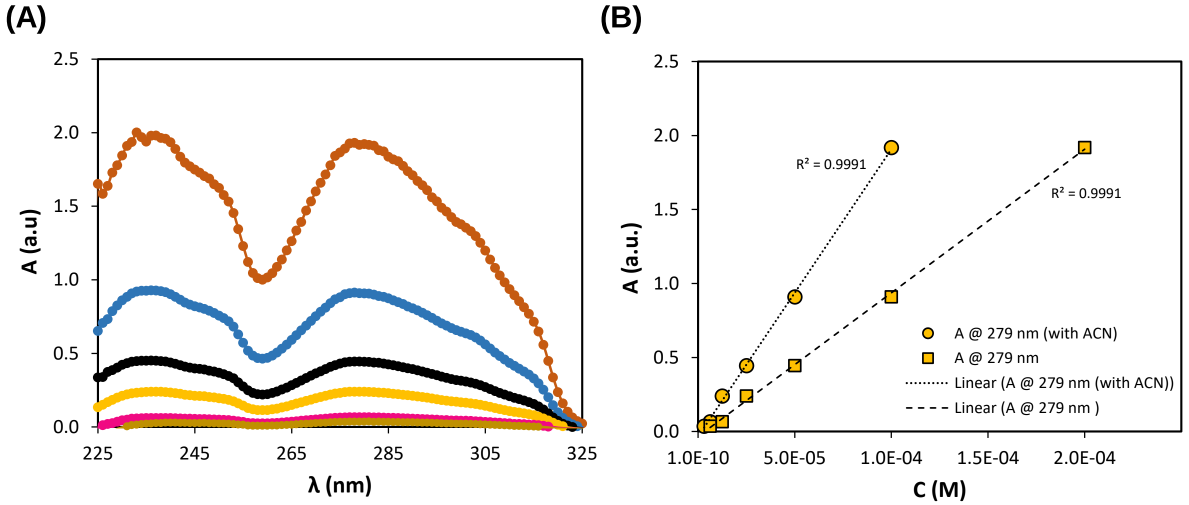

Figure S22: **Calibration used for quantification of the terpyridine groups in the hydrophobic state.** (A) Raw UV-Vis data of terpyridine groups in the oil (DCM/ACN with 1:1 volume ratio) phase. (B) Calibration curve of terpyridine in DCM/ACN (square markers) used for quantification of remained terpyridine in the oil phase (hydrophobic state). Addition of ACN helped with tracing lower concentration of terpyridine in the hydrophobic state, compared to pure DCM (circle markers).

3.4 Determination of the hydrophobic contribution g_H of terpyridine by its solubility in DCM and water

The solubilities of monomeric terpyridine in water (with ascorbic acid, pH $\approx$ 3, saturated with DCM) and in DCM (saturated with water) are measured to be $c_T^{aq} = 3.00 \pm 0.06$ mM and $c_T^{DCM} = 2.80 \pm 0.03$ M, respectively. Taking the chemical potential of terpyridine in the solid as μ^*, we have $\mu_T^{0,aq} + k_B T \ln(c_T^{aq}/c^0) = \mu^*$. Likewise, in DCM, we have $\mu_T^{0,DCM} + k_B T \ln(c_T^{DCM}/c^0) = \mu^*$. Here the $\mu_T^{0,i}$ are the standard chemical potentials of terpyridine in i = aq, DCM at concentration c^0. The reversible work to transfer a terpyridine molecule from DCM to the aqueous phase is given by $g_H = \mu_T^{0,aq} - \mu_T^{0,DCM} = k_B T \ln(c_T^{DCM}/c_T^{aq}) \approx 6.8 k_B T$.

3.5 Partitioning of terpyridine monomer in the two-phase system

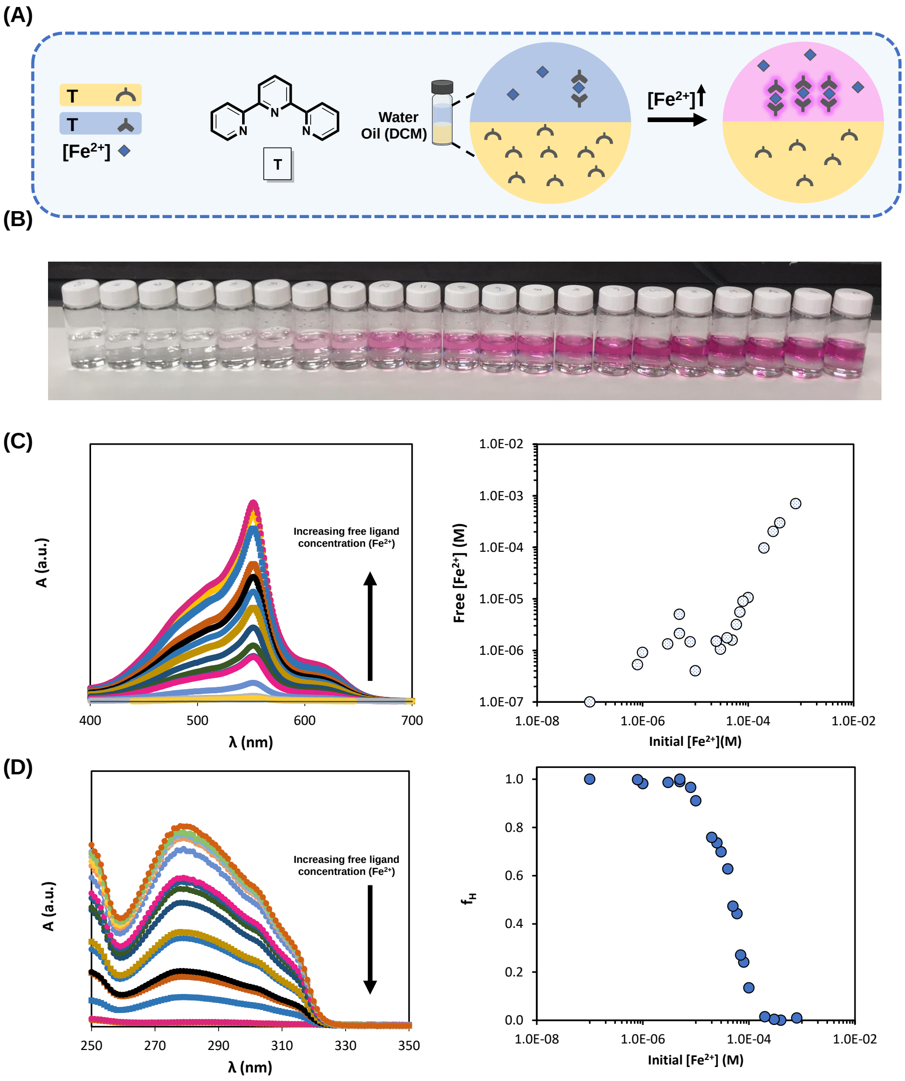

Figure S23: **Partitioning of terpyridine monomer in the two-phase water/DCM system.** (A) Schematic representation of the experimental setup. (B) Visualization of the experiment. Top and bottom phases are water and DCM, respectively. Complexation of terpyridine with iron ions in the water (top) phase upon increasing iron concentration in the water phase (from left to right) is evident by the pink color of the top phase. (C) UV-Vis spectroscopy of terpyridine iron (II) complexes in the water phase (left) and extraction of free iron ion concentration after subtracting the obtained Fe^{2+} concentrations (by UV-Vis) from the values obtained by ICP-AES (right). (D) UV-Vis spectroscopy of terpyridine in the hydrophobic phase (left) and extraction of f_H; fraction of terpyridine in DCM phase (right).

3.6 Partitioning of the terpyridine-functionalized polymer in the two-phase system

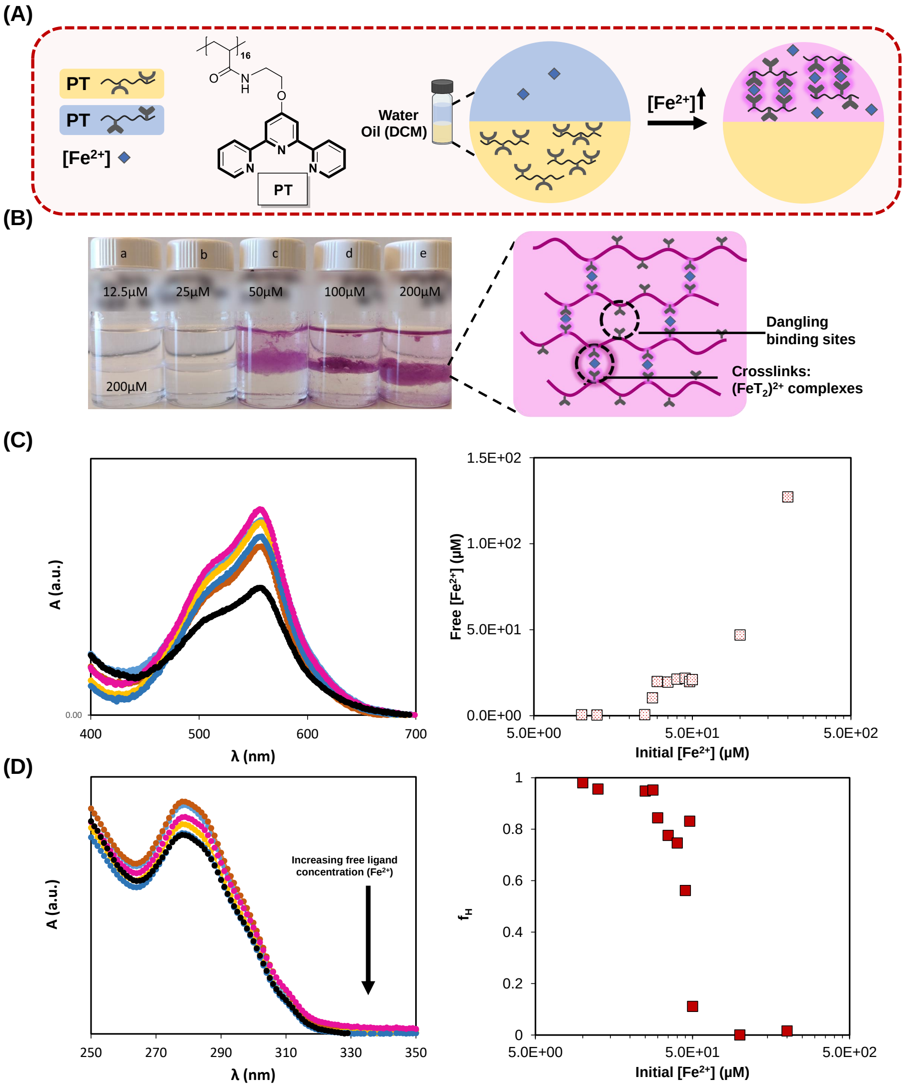

Figure S24: **Partitioning of terpyridine-functionalized polymer in the two-phase water/DCM system.** (A) Schematic representation of the experimental setup. (B) Visualization of the experiment. Top and bottom phases are water and DCM, respectively. Complexation of terpyridine functional groups with iron ions in the water (top) phase upon increasing iron concentration in the water phase (from left to right) is evident by the pink color of the top phase. Clusters of polymer chains bridged by iron ions in the bis terpyridine iron (II) complexes are visible to the eye. (C) UV-Vis spectroscopy of terpyridine iron (II) complexes in the water phase (left) and extraction of free iron ion concentration after subtracting the obtained Fe^{2+} concentrations (by UV-Vis) from the values obtained by ICP-AES (right). (D) UV-Vis spectroscopy of terpyridine in the hydrophobic phase (left) and extraction of f_H; fraction of terpyridine groups (equivalent to the fraction of the polymers) in DCM phase (right).

3.7 Terpyridine monomer partitioning between DCM and water

The experimental results showed a gradual decrease in fraction of terpyridine in the hydrophobic state (f_H) from 1 to 0 upon increasing and over a broad range of free iron concentration in the aqueous phase, see the markers in Figure S25. As it was mentioned in the section 1.2.1, the obtained fraction of terpyridine in the oil (DCM) phase experimentally showed clear deviation from Equation 15. The blue dashed line in Figure S25 is Equation 15 with $[T_{tot}] = 2 \times 10^{-4} M$ (based on the experiments). The best fit based on Equation 15 results in linear combination of binding and hydrophobic energies for each bis (terpyridine) iron (II) complex, $\beta g = -21.8$. However, our results of binding iron ions onto terpyridine pointed to a significantly steeper dependence of the terpyridine fraction in the oil phase on the free iron (II) concentration. To resolve the mentioned discrepancy, we assumed that terpyridine is not in monomeric form in the hydrophobic state, but in the form of pairs. The blue solid line in Figure S25 is the best fit of Equation 17 to the experimental data of terpyridine (blue circles) with $\beta g = -12.8$ and gives a better match for the experiment results, compared to Equation 15.

Overall, it is not clear to us why the experimental data are best described by taking the assumption of formation of terpyridine dimers in the solution in the presented model. Furthermore, it should be noted as well that the partitioning of terpyridine monomer in water in the presence of the oil (DCM) was not measurable due to the detection limit of the UV-Vis spectroscopy.

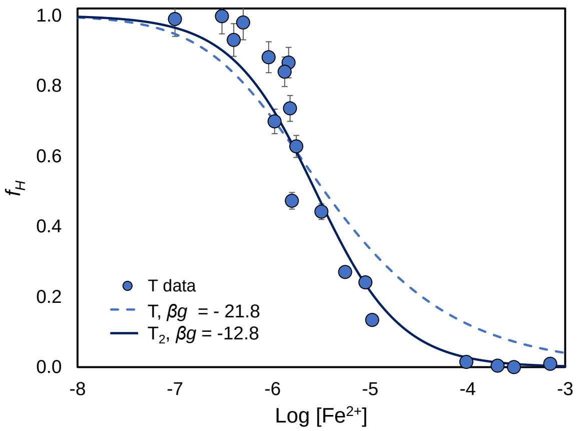

Figure S25: **Comparison between experiments and theory for partitioning of terpyridine monomer between oil (DCM) and water phases**. Fraction of terpyridine (blue markers, T data) in the oil phase (f_H) as a function of the free iron (II) concentration in the aqueous phase. Blue dashed line (T) is Equation 15 which gives the best fit with $\beta g = -21.8$. The blue solid line (T_2)which gives a better description of the data on terpyridine is Equation 17 with $\beta g = -12.8$.

References

(S1) Hill, A. V. The possible effects of the aggregation of the molecules of haemoglobin on its dissociation curves. *Proceedings of the Physiological Society* **1910**, *40*, i–vii.

(S2) Kerr, D. H.; Suwatthee, T.; Maltseva, S.; Turke, M. J.; Lee, K. Y. C. Beyond the hill equation: Reconceptualizing cooperativity for peripheral membrane-binding proteins. *Biophysical Journal* **2024**, *123*, 452a.

(S3) Gesztelyi, R.; Zsuga, J.; Kemeny-Beke, A.; Varga, B.; Juhasz, B.; Tosaki, A. The Hill equation and the origin of quantitative pharmacology. *Archive for history of exact sciences* **2012**, *66*, 427–438.

(S4) Martin Robinson, J. L.; Kegel, W. K. Cooperative transitions involving hydropho-

bic polyelectrolytes. *Proceedings of the National Academy of Sciences* **2023**, *120*, e2211088120.

(S5) Holyer, R.; Hubbard, C.; Kettle, S.; Wilkins, R. The kinetics of replacement reactions of complexes of the transition metals with 2, 2', 2"-terpyridine. *Inorganic Chemistry* **1966**, *5*, 622–625.

(S6) ChemicalBook 2,2':6',2"-Terpyridine. `https://www.chemicalbook.com/ChemicalProductProperty_EN_CB1407532.htm`.

(S7) Bretti, C.; Crea, F.; De Stefano, C.; Sammartano, S. Solubility and activity coefficients of 2, 2'-bipyridyl, 1, 10-phenanthroline and 2, 2', 6', 2"-terpyridine in NaCl (aq) at different ionic strengths and T= 298.15 K. *Fluid phase equilibria* **2008**, *272*, 47–52.

(S8) Harrisson, S. The downside of dispersity: Why the standard deviation is a better measure of dispersion in precision polymerization. *Polymer Chemistry* **2018**, *9*, 1366–1370.

(S9) Monteiro, M. J. Fitting molecular weight distributions using a log-normal distribution model. *European Polymer Journal* **2015**, *65*, 197–201.

(S10) Klumperman, B.; Vonk, G. Solvent effects on the copolymerization of styrene with maleic anhydride: determination of apparent reactivity ratios from the penultimate unit model. *European Polymer Journal* **1994**, *30*, 955–960.

(S11) Brandrup, J.; Immergut, E. H.; Grulke, E. A. *Polymer handbook*, 4th ed.; Wiley: New York, 1999.

(S12) Disdier, Z.; Savoye, S.; Dagnelie, R. V. H. Effect of solutes structure and pH on the

n-octanol/water partition coefficient of ionizable organic compounds. *Chemosphere* **2022**, *304*, 135155.

(S13) Standal, S. H.; Blokhus, A. M.; Haavik, J.; Skauge, A.; Barth, T. Partition Coefficients and Interfacial Activity for Polar Components in Oil/Water Model Systems. *Journal of Colloid and Interface Science* **1999**, *212*, 33–41.

(S14) Harris, D. C. *Quantitative chemical analysis*, 7th ed.; W.H. Freeman and Co.: New York, NY, 2007.

(S15) Perrier, S. 50th Anniversary Perspective: RAFT Polymerization—A User Guide. *Macromolecules* **2017**, *50*, 7433–7447.

(S16) Anastasaki, A.; Nikolaou, V.; Nurumbetov, G.; Wilson, P.; Kempe, K.; Quinn, J. F.; Davis, T. P.; Whittaker, M. R.; Haddleton, D. M. Cu(0)-Mediated Living Radical Polymerization: A Versatile Tool for Materials Synthesis. *Chemical Reviews* **2016**, *116*, 835–877.

(S17) Ribelli, T. G.; Lorandi, F.; Fantin, M.; Matyjaszewski, K. Atom Transfer Radical Polymerization: Billion Times More Active Catalysts and New Initiation Systems. *Macromolecular Rapid Communications* **2019**, *40*, 1800616.

(S18) Gelsema, W. J.; de Ligny, C. L.; Remijnse, A. G.; Blijleven, H. A. pH-Measurements in alcohol-water mixtures, using aqueous standard buffer solutions for calibration. *Recueil des Travaux Chimiques des Pays-Bas* **1966**, *85*, 647–660.

(S19) Bates, R. G.; Paabo, M.; Robinson, R. A. Interpretation of pH measurements in alocohol—water solvents. *The Journal of Physical Chemistry* **1963**, *67*, 1833–1838.

(S20) Hetzer, M.; Schmidt, B. V. K. J.; Barner-Kowollik, C.; Ritter, H. Limitations of

cyclodextrin-mediated RAFT homopolymerization and block copolymer formation. *Journal of Polymer Science Part A: Polymer Chemistry* **2013**, *51*, 2504–2517.

(S21) Brodszkij, E.; Hviid, M. J.; Ade, C.; Schattling, P. S.; Burmeister, M.; Szilagyi, S.; Gal, N.; Zhu, C.; Han, X.; Städler, B. Interaction of pH-responsive polyanions with phospholipid membranes. *Polym. Chem.* **2019**, *10*, 5992–5997.

(S22) Chen, F.; Cheng, Z.; Zhu, J.; Zhang, W.; Zhu, X. Synthesis of poly(vinyl acetate) with fluorescence via a combination of RAFT/MADIX and "click" chemistry. *European Polymer Journal* **2008**, *44*, 1789–1795.

(S23) van Ravensteijn, B. G. P.; Bou Zerdan, R.; Helgeson, M. E.; Hawker, C. J. Minimizing Star–Star Coupling in Cu(0)-Mediated Controlled Radical Polymerizations. *Macromolecules* **2019**, *52*, 601–609.

(S24) Anastasaki, A.; Willenbacher, J.; Fleischmann, C.; Gutekunst, W. R.; Hawker, C. J. End group modification of poly(acrylates) obtained via ATRP: a user guide. *Polym. Chem.* **2017**, *8*, 689–697.

(S25) Honda, S.; Tanaka, N.; Toyota, T. Synthesis of star-shaped poly(n-butyl acrylate) oligomers with coumarin end groups and their networks for a UV-tunable viscoelastic material. *Journal of Polymer Science Part A: Polymer Chemistry* **2018**, *56*, 9–15.

(S26) Hu, L.; Liu, W.; Li, C. H.; Zhou, X. H.; Zuo, J. L. Iron(II) complexes based on π-conjugated terpyridine ligands with tetrathiafulvalene or its radical analogue. *European Journal of Inorganic Chemistry* **2013**, 6037–6048.

(S27) Belhadj, E.; El-Ghayoury, A.; Ripaud, E.; Zorina, L.; Allain, M.; Batail, P.; Mazari, M.; Sallé, M. Terpyridine – tetrathiafulvalene hybrid ligands and their electroactive metal complexes. *New Journal of Chemistry* **2013**, *37*, 1427–1436.
